# Clinical Practice Guideline for Rehabilitation Nutrition 2026: Executive Summary of the Japanese Association of Rehabilitation Nutrition Guideline 2026—A Secondary Publication

**DOI:** 10.31662/jmaj.2026-0293

**Published:** 2026-08-14

**Authors:** Yoshihiro Yoshimura, Yusuke Yasumoto, Tomohiro Matsumoto, Ayano Nagano, Shinta Nishioka, Shinsuke Nagami, Fumihiko Nagano, Mai Tanaka, Yuki Iida, Youngjae Hong, Hideki Aragane, Atsuo Kitaoka, Kazuharu Nakagawa, Kanako Yoshimi, Hideaki Takahata, Ai Shiraishi, Yoko Saino, Hitomi Ichishima, Ayaka Matsumoto, Keiichiro Higashi, Naoko Kumagae, Dai Fujiwara, Kenji Omura, Hidetaka Wakabayashi

**Affiliations:** 1 Center for Sarcopenia and Malnutrition Research, Kumamoto Rehabilitation Hospital, Kumamoto, Japan; 2 Department of General Medicine, Itabashi Chuo Medical Center, Tokyo, Japan; 3 Department of Internal Medicine, Uenohara City Hospital, Yamanashi, Japan; 4 Department of Nursing, Nishinomiya Kyoritsu Neurosurgical Hospital, Hyogo, Japan; 5 Graduate School of Regional Design and Creation, University of Nagasaki, Nagasaki, Japan; 6 Department of Communication Disorders, School of Rehabilitation Sciences, Health Sciences University of Hokkaido, Hokkaido, Japan; 7 Department of Rehabilitation, Tonami General Hospital, Toyama, Japan; 8 Faculty of Health Sciences, Aichi Shukutoku University, Aichi, Japan; 9 Department of Social Welfare, Nihon Fukushi University, Aichi, Japan; 10 Department of Surgery, Aiseikai Yamashina Hospital, Kyoto, Japan; 11 Division of Clinical Nutrition and Food Services, Kagawa University Hospital, Kagawa, Japan; 12 Department of Dysphagia Rehabilitation, Graduate School of Medical and Dental Sciences, Institute of Science Tokyo, Tokyo, Japan; 13 Department of Rehabilitation Medicine, Nagasaki University Hospital, Nagasaki, Japan; 14 Department of Oral Surgery, Tokyo Women’s Medical University Hospital, Tokyo, Japan; 15 Department of Clinical Nutrition, Cancer Institute Hospital, Japanese Foundation for Cancer Research, Tokyo, Japan; 16 Department of Clinical Nutrition and Food Service, Saiseikai Otaru Hospital, Hokkaido, Japan; 17 Department of Pharmacy, Asanogawa General Hospital, Ishikawa, Japan; 18 Department of Nutritional Service, Yokohama City University Medical Center, Kanagawa, Japan; 19 Department of Rehabilitation Medicine, Saka General Hospital, Miyagi, Japan; 20 Department of Surgery, Ageo Central General Hospital, Saitama, Japan; 21 Department of Rehabilitation Medicine, Tokyo Women’s Medical University Hospital, Tokyo, Japan

**Keywords:** cerebrovascular disease, proximal femur fracture, chronic obstructive pulmonary disease, cancer, dysphagia, pharmaceutical intervention, hospital-associated sarcopenia, social determinants of health

## Abstract

The Japanese Association of Rehabilitation Nutrition (JARN) published its first Japanese-language clinical practice guideline on rehabilitation nutrition in 2018, followed by an English-language 2020 update covering cerebrovascular disease, hip fracture, cancer, and acute illness. The JARN Clinical Practice Guideline 2026 (JARN GL 2026) expands the framework to 11 domains and incorporates updated evidence on malnutrition diagnosis using the Global Leadership Initiative on Malnutrition criteria, sarcopenia assessment, and interactions between nutritional therapy and rehabilitation. This secondary publication summarizes the scope, methods, and all statements.

JARN GL 2026 was developed in accordance with the Minds Clinical Practice Guideline Development Manual 2020 (version 3.0). Clinical questions were formulated in Population, Intervention, Comparison, and Outcome format and classified as clinical questions (CQs) with graded recommendations, background questions (BQs) providing evidence-based context, or future research questions (FRQs) identifying topics with insufficient evidence. Recommendations were graded for strength (1 = strong; 2 = weak/conditional) and certainty of evidence (A-D). Patient and public involvement and external peer review by related academic societies were incorporated.

Across 11 domains—cerebrovascular disease, proximal femur fracture, chronic obstructive pulmonary disease (COPD), cancer, dysphagia, intensive exercise therapy, enhanced nutritional care, oral management, pharmaceutical intervention, hospital-associated sarcopenia, and social determinants of health—12 CQs, five BQs, and three FRQs are presented. Enhanced nutritional care combined with rehabilitation was strongly recommended for patients with proximal femur fracture and weakly recommended for patients with acute- and convalescent-phase cerebrovascular disease, COPD, cancer, and dysphagia. Registered dietitian-led nutritional assessment and counseling and professional oral management concurrent with rehabilitation were each weakly recommended. Three topics were classified as FRQs because of insufficient evidence.

JARN GL 2026 provides a systematically developed, evidence-graded framework to support patient-centered, multidisciplinary rehabilitation nutrition across care settings.

## Background

JARN published its first clinical practice guideline on rehabilitation nutrition in 2018 ^(1), (2), (3), (4)^, covering four clinical domains and establishing a systematic framework that integrates nutritional therapy with rehabilitation medicine. An English-language 2020 update ^(5)^ was subsequently published, providing evidence-based recommendations for rehabilitation nutrition in four domains: cerebrovascular disease, hip fracture, cancer, and acute illness. Rehabilitation nutrition is a patient-centered, multidisciplinary clinical approach that integrates nutritional management with rehabilitation to simultaneously address nutritional disorders—malnutrition, sarcopenia, frailty, and cachexia—and functional impairment. Its core process comprises assessment, diagnosis, goal setting, intervention, and monitoring, with individualized decisions based on disease status, comorbidities, living circumstances, patient values, and available resources.

Since then, population aging, community-integrated care, Global Leadership Initiative on Malnutrition -based malnutrition diagnosis, revised sarcopenia criteria, and accumulating evidence on exercise–nutrition interactions have increased the need for updated rehabilitation nutrition guidance.

JARN GL 2026 ^(6)^ reassesses the evidence for domains covered in the 2018 guideline and extends coverage to 11 domains in total. The guideline aims to (1) promote patient-centered care that supports functional recovery and improvements in activities of daily living (ADL), quality of life (QOL), and prognosis; (2) provide a practicable framework for multidisciplinary collaboration; and (3) identify priorities for future research.

## Scope and Intended Users

JARN GL 2026 addresses the rehabilitation nutrition process—assessment, diagnosis, goal-setting, intervention, and monitoring—across acute, recovery, home, and community settings.

Three of the four disease domains from the 2018 edition (cerebrovascular disease, proximal femur fracture, and cancer; acute illness was not carried forward) were retained and updated. Eight additional domains were incorporated: dysphagia, COPD, enhanced nutritional care, intensive exercise therapy, oral management, pharmaceutical intervention, hospital-associated sarcopenia, and social determinants of health (SDH). Interventions include multidisciplinary nutritional therapy and rehabilitation, with pharmacotherapy and social resource coordination as appropriate. Primary outcomes of interest include physical function, ADL, QOL, complications, prognosis, and patient-reported outcomes.

The guideline is intended primarily for multiprofessional rehabilitation nutrition providers, including physicians, dentists, nurses, registered dietitians, pharmacists, physical therapists, occupational therapists, speech-language-hearing therapists, and dental hygienists. It also informs care managers, social workers, patients, families, and individualized decision-making.

### Guideline development methods

JARN GL 2026 was developed by JARN in accordance with the Minds Clinical Practice Guideline Development Manual 2020 (ver. 3.0). The scope was prespecified, and CQs were formulated in Population, Intervention, Comparison, and Outcome format and prioritized by clinical importance, feasibility, and evidence availability. Questions without sufficient evidence for CQ-based recommendations were classified as BQs or FRQs.

The development structure comprised a supervisory committee, a multidisciplinary guideline development group, a systematic review team, and a secretariat. The systematic review team screened and appraised evidence independently from the recommendation panel. For each CQ, the panel deliberated on evidence certainty, the balance of benefits and harms, patient values and preferences, feasibility, and resource implications, and reached consensus on the statement, recommendation strength, and certainty rating. BQs were addressed through narrative evidence synthesis, and FRQs were formulated to guide future research agendas without constituting recommendations. Supplementary Figures and Supplementary Tables provide PRISMA flow diagrams and structured evidence summaries for each relevant question.

Patient and public involvement was embedded in the development process: a patient and family representative participated in the guideline panel. External peer review was conducted by four evaluators representing related academic societies, and opinions were solicited from relevant professional organizations. JARN provided all funding, and COIs were declared, managed, and disclosed in accordance with Minds and JARN regulations.

### Ethics approval

Institutional review board approval was not obtained because it was not required. This manuscript is an English executive summary and secondary publication of a previously published Japanese clinical practice guideline based on systematic literature review, evidence synthesis, guideline panel deliberation, patient and public involvement, and external peer review. No new human participants were recruited, no intervention was performed, and no individual-level or identifiable patient data were collected, analyzed, or reported.

### Summary of statements

Statements are organized by the 11 domains. For CQs, recommendation strength and certainty of evidence are shown; BQs provide contextual evidence, and FRQs indicate insufficient evidence for recommendation. Table 1 summarizes all 20 items (Table 1).

**Table 1.** Clinical Questions, Background Questions, and Future Research Questions Addressed in this Guideline.

Domain	No.	Question type	Question
Cerebrovascular Disease	1	CQ1	Should enhanced nutritional care be provided to patients with acute cerebrovascular disease undergoing rehabilitation?
Cerebrovascular Disease	2	CQ2	Should enhanced nutritional care be provided to patients with cerebrovascular disease in the convalescent phase undergoing rehabilitation?
Proximal Femoral Fracture	3	CQ3	Should enhanced nutritional care be provided to patients with proximal femoral fracture undergoing rehabilitation?
Chronic Obstructive Pulmonary Disease (COPD)	4	CQ4	Should enhanced nutritional care be provided to outpatients and inpatients with chronic obstructive pulmonary disease undergoing rehabilitation?
Cancer	5	CQ5	Should a combined rehabilitation and nutritional therapy program be provided to adult patients with solid tumors before or after treatment or with advanced or recurrent disease, excluding refractory cachexia?
Dysphagia	6	BQ1	What exercise therapies are available for patients with dysphagia?
Dysphagia	7	BQ2	What nutritional therapies are available for patients with dysphagia?
Dysphagia	8	BQ3	What clinical problems are associated with malnutrition in patients with dysphagia?
Dysphagia	9	CQ6	Should exercise therapy be provided to patients with dysphagia?
Dysphagia	10	CQ7	Should exercise therapy and nutritional therapy be combined to improve swallowing function in patients with dysphagia?
Dysphagia	11	CQ8	Should nutritional therapy, including enteral nutrition, parenteral nutrition, oral nutritional supplements, and nutritional counseling, be provided to patients with dysphagia?
Intensive Exercise Therapy	12	FRQ1	Should intensive exercise therapy be provided to patients with malnutrition receiving nutritional therapy?
Enhanced Nutritional Care	13	BQ4	What is the range of energy expenditure in older adults aged 65 years or older undergoing rehabilitation, and are there simple methods for estimating energy expenditure?
Enhanced Nutritional Care	14	CQ9	Should registered dietitians perform nutritional assessment for older adults undergoing rehabilitation in outpatient and inpatient settings, older adults requiring long-term care, and frail older adults?
Enhanced Nutritional Care	15	CQ10	Should registered dietitians provide nutritional counseling to older adults undergoing rehabilitation in outpatient and inpatient settings, older adults requiring long-term care, and frail older adults?
Oral Management	16	CQ11	Does combining professional oral management with rehabilitation improve outcomes compared with rehabilitation alone in rehabilitation inpatients?
Pharmaceutical Intervention	17	CQ12	Is pharmacist-led pharmaceutical intervention effective in patients undergoing rehabilitation?
Hospital-Associated Sarcopenia	18	BQ5	What is the incidence of hospital-associated sarcopenia, and what are its causes and risk factors?
Social Determinants of Health	19	FRQ2	Is screening for social determinants of health useful for improving outcomes in adults receiving rehabilitation treatment or exercise therapy?
Social Determinants of Health	20	FRQ3	Do interventions that take social determinants of health into account improve outcomes, including treatment continuation, activities of daily living, and healthcare costs, in adults receiving rehabilitation treatment or exercise therapy?

BQ: background question; COPD: chronic obstructive pulmonary disease; CQ: clinical question; FRQ: future research question.

In this summary, “enhanced nutritional care” refers to nutritional interventions implemented with the aim of increasing energy or nutrient intake in addition to a regular diet or standard nutritional management. This includes ONS, nutrient fortification, increased enteral or parenteral delivery, and individualized management by registered dietitians or equivalent professionals.

### Domain 1: Cerebrovascular disease

#### CQ1. Should enhanced nutritional care be provided to patients with acute cerebrovascular disease undergoing rehabilitation?

**Statement:** In patients with acute cerebrovascular disease undergoing rehabilitation, enhanced nutritional care using oral nutritional supplements or early enteral nutrition is weakly recommended, with careful attention to blood glucose levels.

#### Recommendation strength: 2 / Certainty of evidence: C

**Summary of rationale:** Stroke is a leading cause of death and disability worldwide, and because malnutrition from the acute phase is associated with poor outcomes, maintaining adequate nutrition alongside exercise-based interventions is essential ^(7), (8), (9), (10), (11)^. In 34 randomized controlled trials (RCTs) involving patients with acute cerebrovascular disease, enhanced nutritional care showed trends toward improvement in ADL, body composition, body weight, and mortality; however, consistent statistically significant effects were not observed across most outcomes, and the certainty of evidence was low ^(12), (13), (14), (15), (16), (17), (18), (19), (20), (21), (22), (23), (24), (25), (26), (27), (28), (29), (30), (31), (32), (33), (34), (35), (36), (37), (38), (39), (40), (41), (42), (43), (44), (45)^. Oral nutritional supplements and early enteral nutrition may be useful, but adverse events, including diarrhea and hyperglycemia or hypoglycemia, have been reported; therefore, careful monitoring, including glycemic management, is required.

#### CQ2. Should enhanced nutritional care be provided to patients with cerebrovascular disease in the convalescent phase undergoing rehabilitation?

**Statement:** In patients with cerebrovascular disease in the convalescent phase undergoing rehabilitation, enhanced nutritional therapy, such as additional protein provision based on adequate energy intake, is weakly recommended.

#### Recommendation strength: 2 / Certainty of evidence: C

**Summary of rationale:** In recovery-phase cerebrovascular disease, malnutrition is highly prevalent in rehabilitation wards and may adversely affect ADL outcomes and home discharge, underscoring the need for proactive nutritional therapy to support maximal recovery ^(10), (46)^. In three RCTs involving mixed acute/convalescent populations and 10 RCTs in the convalescent phase, enhanced nutritional care using oral nutritional supplements—particularly energy- and protein-enriched formulations—showed trends toward improvement in ADL, physical function, and muscle strength, but effects were inconsistent across nutrient types and interventions, and clear statistically significant differences were not observed for many outcomes ^(47), (48), (49), (50), (51), (52), (53), (54), (55), (56), (57), (58), (59)^. Few adverse events were reported, and oral nutritional supplements are relatively low-cost and feasible. Implementation should be individualized.

### Domain 2: Proximal femoral fracture

#### CQ3. Should enhanced nutritional care be provided to patients with proximal femoral fracture undergoing rehabilitation?

**Statement:** In patients with proximal femoral fracture undergoing rehabilitation, enhanced nutritional care improves ADL and physical function, shortens length of hospital stay, and reduces the incidence of complications, with an expected improvement in mortality. Considering the economic burden on healthcare facilities and patient preferences, its feasibility in clinical practice is considered high. Therefore, enhanced nutritional care is strongly recommended.

#### Recommendation strength: 1 / Certainty of evidence: C

Summary of rationale: In patients with proximal femoral fracture undergoing rehabilitation, pooled analyses of RCTs showed significant improvement in ADL, grip strength, and body mass index (BMI), shortening of length of hospital stay, and reduction in complications ^(60), (61), (62), (63), (64), (65), (66), (67), (68), (69), (70), (71), (72), (73), (74), (75), (76), (77), (78), (79)^. Mortality also tended to decrease. No clear adverse events were reported, and the benefits were judged to outweigh the harms. Although the certainty of evidence was low, outcomes related to functional recovery and prognosis are important to patients, and the therapy is considered acceptable in many cases. Uncertainty remains regarding cost-effectiveness; however, feasibility is generally high, and implementation should take into account the continuity of nutritional intake and patient motivation.

### Domain 3: Chronic obstructive pulmonary disease (COPD)

#### CQ4. Should enhanced nutritional care be provided to outpatients and inpatients with chronic obstructive pulmonary disease undergoing rehabilitation?

**Statement:** In outpatients and inpatients with COPD undergoing rehabilitation, enhanced nutritional care is weakly recommended.

#### Recommendation strength: 2 / Certainty of evidence: C

**Summary of rationale:** COPD imposes a substantial disease burden in Japan ^(80)^, and pulmonary rehabilitation is a comprehensive intervention that includes exercise, education, and nutritional management ^(81), (82), (83), (84)^. The present review built on the previous systematic review by Aldhahir et al. ^(85)^. In 10 RCTs involving patients with COPD ^(86), (87), (88), (89), (90), (91), (92), (93), (94), (95)^, enhanced nutritional care improved muscle strength, body weight, and BMI; however, consistent effects were not observed for exercise tolerance, QOL, or pulmonary function. Because of heterogeneity and risk-of-bias concerns, certainty of evidence was rated as C. Safety data were limited, so indications should be individualized.

### Domain 4: Cancer

#### CQ5. Should a combined rehabilitation and nutritional therapy program be provided to adult patients with solid tumors before or after treatment or with advanced or recurrent disease, excluding refractory cachexia?

**Statement:** In adult patients with solid tumors before or after treatment or with advanced or recurrent disease, excluding the refractory cachexia stage, no serious adverse events have been reported for programs combining rehabilitation and nutritional therapy. These programs have been suggested to be useful for increasing protein intake, improving body composition, and alleviating cancer-related symptoms, such as fatigue, nausea, and vomiting. Therefore, because improvements in beneficial outcomes, including muscle strength, body composition, and fatigue, can be expected, a combined rehabilitation and nutritional therapy program is weakly recommended.

#### Recommendation strength: 2 / Certainty of evidence: C

**Summary of rationale:** Patients with cancer face a high risk of malnutrition and sarcopenia induced by the disease and by treatment-related factors; integration of rehabilitation and nutritional therapy is therefore recognized as a vital strategy to preserve ADL and QOL, even from the pre-cachexia stage. In an assessment of five RCTs, combined rehabilitation and nutritional therapy significantly improved muscle strength, body composition, and fatigue. Consistent results were not obtained for overall QOL, survival, or general nutritional status, and certainty was low. No serious adverse events were reported. Many positive studies were limited to patients with prostate cancer receiving androgen deprivation therapy, who are predisposed to sarcopenic obesity, limiting generalizability ^(96), (97), (98), (99), (100), (101), (102), (103), (104)^.

### Domain 5: Dysphagia

#### BQ1. What exercise therapies are available for patients with dysphagia?

**Statement:** Exercise therapies for patients with dysphagia include suprahyoid muscle-strengthening exercises, such as head-lift exercise and chin-tuck against resistance, tongue resistance training, and expiratory resistance training. Studies involving patients after surgery for head and neck cancer, post-stroke patients, patients with Parkinson disease, and institutionalized older adults have reported effects such as increased hyoid excursion, increased suprahyoid muscle mass, and reduced aspiration risk. Improvements in swallowing function have also been reported with comprehensive exercise therapy, including whole-body exercise.

**Summary of rationale:** Weakness of swallowing-related muscles contributes to impairment across the phases of swallowing, and exercise therapies targeting the suprahyoid muscles and tongue are expected to improve swallowing function. Comprehensive approaches that include interventions for respiratory function and whole-body function may also be effective. Exercise load should be individualized ^(105), (106), (107), (108), (109), (110), (111), (112), (113), (114), (115), (116), (117), (118), (119), (120), (121), (122), (123), (124), (125), (126), (127), (128), (129), (130), (131), (132)^.

#### BQ2. What nutritional therapies are available for patients with dysphagia?

**Statement:** For patients with dysphagia who are able to take food orally, nutritional therapy includes modification of food texture according to swallowing function, provision of nutrient-dense foods, and oral nutritional supplements; these approaches have been reported to increase body weight, improve nutritional status, and improve physical function. For patients with dysphagia in whom oral intake is difficult, alternative nutritional routes, such as nasogastric tube feeding and percutaneous endoscopic gastrostomy (PEG), are options and may reduce mortality risk and improve nutritional status in the acute phase. However, in older patients with dysphagia, aspiration pneumonia and mortality have been reported to be higher in those receiving alternative nutrition; therefore, transition to oral intake should also be considered when appropriate.

**Summary of rationale:** When oral intake is possible, texture modification and nutritional fortification are expected to improve nutritional status and function. When oral intake is difficult, alternative nutrition should be used appropriately as a safe means of securing nutritional intake. However, it should not be assumed that patients will remain dependent on alternative nutrition over the long term. It is important to concurrently provide dysphagia rehabilitation while repeatedly assessing swallowing function over time, taking into account the patient’s overall condition, prognosis, and living circumstances ^(36), (133), (134), (135), (136), (137), (138), (139), (140), (141), (142), (143), (144), (145), (146), (147), (148)^.

#### BQ3. What clinical problems are associated with malnutrition in patients with dysphagia?

**Statement:** Malnutrition in patients with dysphagia is associated with deterioration in nutritional indicators, such as weight loss and low BMI. It is also associated with poor prognostic factors, including reduced swallowing function and increased mortality. Therefore, early nutritional assessment and appropriate nutritional intervention are required for patients with dysphagia.

**Summary of rationale:** The evidence review showed a certain prevalence of malnutrition among patients with dysphagia ^(149)^, as well as low BMI and reduced energy and protein intake ^(150), (151), (152), (153), (154)^. In addition, dysphagia severity, duration of dysphagia, and physical function measures were associated with malnutrition ^(153)^. The evidence also suggested that nutritional supplementation alone may be insufficient to improve body composition ^(150)^.

#### CQ6. Should exercise therapy be provided to patients with dysphagia?

**Statement:** Exercise therapy is weakly recommended for patients with dysphagia undergoing rehabilitation in outpatient and inpatient settings, older adults requiring long-term care, and frail older adults, because improvement in swallowing function can be expected.

#### Recommendation strength: 2 / Certainty of evidence: D

**Summary of rationale:** The evidence review included 34 RCTs. Exercise therapies, including head-lift exercise, chin-tuck against resistance, tongue resistance training, and respiratory muscle training, showed improvements in tongue pressure, the Videofluoroscopic Dysphagia Scale, and the Functional Oral Intake Scale. Although the target populations and intervention methods varied, the effects were small but consistently indicated improvement in swallowing function. The Penetration–Aspiration Scale showed only a trend toward improvement; nevertheless, many studies reported improvements in swallowing-related outcomes. Because of heterogeneity in populations, interventions, and outcome measures, as well as risk of bias, the certainty of evidence was rated as D ^(105), (106), (108), (109), (110), (111), (112), (113), (114), (115), (116), (117), (118), (120), (121), (122), (123), (124), (127), (128), (129), (155), (156), (157), (158), (159), (160), (161), (162), (163), (164), (165), (166), (167), (168), (169), (170)^. Exercise selection and load should be individualized.

#### CQ7. Should exercise therapy and nutritional therapy be combined to improve swallowing function in patients with dysphagia?

**Statement:** For patients with dysphagia undergoing rehabilitation in outpatient and inpatient settings, older adults requiring long-term care, and frail older adults, the combined use of exercise therapy and nutritional therapy is weakly recommended because improvements in swallowing function and the rate of transition to oral intake can be expected.

#### Recommendation strength: 2 / Certainty of evidence: D

**Summary of rationale:** In older adults requiring long-term care, malnutrition is directly and indirectly associated with dysphagia, and sarcopenia itself is an important risk factor ^(171), (172), (173)^. The evidence review included 3 RCTs. The combined use of exercise therapy and nutritional therapy improved oral and swallowing function, increased the rate of transition to oral intake, and improved QOL ^(174), (175), (176)^. However, quantitative synthesis was difficult because of heterogeneity in target populations and intervention components, as well as the small number of studies. Therefore, the certainty of evidence was rated as D. When implementing combined therapy, individualized intervention according to the patient’s condition and nutritional status, as well as appropriate timing and intensity, is important.

#### CQ8. Should nutritional therapy, including enteral nutrition, parenteral nutrition, oral nutritional supplements, and nutritional counseling, be provided to patients with dysphagia?

**Statement:** For patients with dysphagia undergoing rehabilitation in outpatient and inpatient settings, older adults requiring long-term care, and frail older adults, nutritional therapy, including enteral nutrition, parenteral nutrition, oral nutritional supplements, and nutritional counseling, is weakly recommended because improvement in swallowing function and nutritional status can be expected.

#### Recommendation strength: 2 / Certainty of evidence: D

**Summary of rationale:** The evidence review included 8 RCTs. Nutritional therapy improved swallowing function ^(175), (177)^, nutritional intake ^(133), (140)^, nutritional indicators ^(40), (133), (177)^, and QOL ^(175)^. However, because outcome measures and intervention components varied across studies, quantitative synthesis was limited. No clear effects were demonstrated for mortality ^(36), (40)^ or length of hospital stay ^(36)^. No adverse events were reported, and undesirable effects were considered small. Content and timing should be individualized by a multidisciplinary team.

### Domain 6: Intensive Exercise Therapy

#### FRQ1. Should intensive exercise therapy be provided to patients with malnutrition receiving nutritional therapy?

**Statement:** There is insufficient evidence to support the efficacy and safety of intensive exercise therapy for patients with malnutrition receiving nutritional therapy. At present, the definition of intensive exercise therapy is unclear, and no conclusion can be drawn regarding the conditions, timing, or exercise components under which it should be implemented. Therefore, no guideline recommendation is made for this item, and it is designated as a Future Research Question.

**Summary of rationale:** Evidence on intensive exercise therapy in patients with malnutrition is extremely limited, and definitions and implementation conditions have not been established ^(178), (179), (180)^. Future high-quality RCTs are needed to examine appropriate exercise intensity and timing according to disease category and nutritional status, as well as safety and effects on ADL and QOL.

### Domain 7: Enhanced Nutritional Care

#### BQ4. What is the range of energy expenditure in older adults aged 65 years or older undergoing rehabilitation, and are there simple methods for estimating energy expenditure?

**Statement:** Resting energy expenditure estimated using indirect calorimetry in older adults aged 65 years or older ranges from 967.5 to 1,703 kcal. Simple methods for estimating energy expenditure in populations have been reported. However, no estimation method can be recommended for use at the individual level.

**Summary of rationale:** Studies using indirect calorimetry have reported that energy expenditure in older adults aged 65 years or older ranges from 967.5 to 1,703 kcal. The validity of estimation methods, including the Harris–Benedict equation, Nachmani equation, Owen equation, and recommended energy requirements proposed by the World Health Organization and the Food and Agriculture Organization of the United Nations, has been examined. However, these methods have been evaluated at the population level, and their validity at the individual level has not been established ^(181), (182), (183), (184), (185), (186), (187), (188)^.

#### CQ9. Should registered dietitians perform nutritional assessment for older adults undergoing rehabilitation in outpatient and inpatient settings, older adults requiring long-term care, and frail older adults?

**Statement:** In older adults undergoing rehabilitation in outpatient and inpatient settings, older adults requiring long-term care, and frail older adults, although consistent effects have not been demonstrated, improvements in skeletal muscle mass, physical and mental function, ADL, and QOL have been suggested. Therefore, it is suggested that registered dietitians perform nutritional assessment before providing nutritional support.

#### Recommendation strength: 2 / Certainty of evidence: C

**Summary of rationale:** The literature search identified six eligible studies, of which two were included in the meta-analysis. Quantitative synthesis was possible only for 180-day mortality, and no statistically significant improvement in mortality was observed with nutritional assessment followed by nutritional support (risk ratio, 0.48; 95% confidence interval, 0.14-1.70). Some studies reported favorable effects on skeletal muscle mass, 6-minute walk distance, the proportion of patients with decline in the Barthel Index, Functional Independence Measure scores, and EuroQol 5-Dimension. Based on these findings, nutritional assessment by registered dietitians is suggested. However, it should be noted that the eligible studies evaluated nutritional assessment together with subsequent nutritional intervention, rather than nutritional assessment alone ^(43), (189), (190), (191), (192), (193), (194), (195), (196), (197), (198), (199), (200), (201), (202), (203)^.

#### CQ10. Should registered dietitians provide nutritional counseling to older adults undergoing rehabilitation in outpatient and inpatient settings, older adults requiring long-term care, and frail older adults?

**Statement:** In older adults undergoing rehabilitation in outpatient and inpatient settings, older adults requiring long-term care, and frail older adults, nutritional counseling by registered dietitians is suggested because improvement in physical function can be expected.

#### Recommendation strength: 2 / Certainty of evidence: D

**Summary of rationale:** The literature search identified five eligible studies, of which four were included in the meta-analysis. No statistically significant intervention effect was observed for mid-upper arm circumference, for which quantitative synthesis was possible (mean between-group difference, 0 cm; 95% confidence interval, −0.94 to 0.94), or for grip strength (mean between-group difference, 0.02 kg; 95% confidence interval, −2.14 to 2.18). However, all four studies that assessed physical function reported improvement. Based on these findings, nutritional counseling by registered dietitians is suggested. It should be noted, however, that many of the eligible studies evaluated multicomponent interventions, including exercise intervention and provision of oral nutritional supplements, rather than nutritional counseling alone ^(202), (204), (205), (206), (207), (208)^.

### Domain 8: Oral Management

#### CQ11. Does combining professional oral management with rehabilitation improve outcomes compared with rehabilitation alone in rehabilitation inpatients?

**Statement:** In hospitalized patients undergoing rehabilitation, compared with rehabilitation alone, the combined use of specialized oral management is expected to improve outcomes such as swallowing function, nutritional status, oral intake rate, activities of daily living, shortened length of hospital stay, higher home discharge rate, and lower mortality. Therefore, oral care intervention in parallel with rehabilitation is weakly recommended for hospitalized rehabilitation patients.

#### Recommendation strength: 2 / Certainty of evidence: D

**Summary of rationale:** Oral management may improve ADL and help prevent aspiration pneumonia. A retrospective cohort study of professional oral management by dental hygienists at least twice weekly reported significantly higher ADL at discharge versus a propensity score–matched control group, with shorter stay and higher home discharge rate; another report found no short-term ADL improvement. Most evidence is observational, so causal inference is limited ^(209), (210), (211), (212), (213), (214)^.

### Domain 9: Pharmaceutical Intervention

#### CQ12. Is pharmacist-led pharmaceutical intervention effective in patients undergoing rehabilitation?

**Statement:** For patients undergoing rehabilitation in outpatient and inpatient settings, pharmacist-led pharmaceutical intervention may contribute to improvement in QOL and may be effective in reducing adverse drug reactions. Therefore, its implementation is weakly recommended.

#### Recommendation strength: 2 / Certainty of evidence: D

**Summary of rationale:** In 11 studies, including eight RCTs and before-and-after studies involving patients undergoing rehabilitation, pharmacist-led interventions—including prescription review, identification of drug-related problems, prescription change proposals, and patient education—were suggested to contribute to improvement in QOL and reduction of adverse drug reactions. Interpretation was limited by heterogeneity in outcome measures and study designs, and the certainty of evidence was rated as D ^(215), (216), (217), (218), (219), (220), (221), (222), (223), (224), (225)^.

### Domain 10: Hospital-Associated Sarcopenia

#### BQ5. What is the incidence of hospital-associated sarcopenia, and what are its causes and risk factors?

**Statement:** The proportion of patients who developed sarcopenia during hospitalization or within several months after discharge ranged from 12.0% to 41.9%, depending on the target population and timing of assessment. Risk factors for developing sarcopenia included prolonged bed rest, low muscle mass, low BMI, low ADL, and prolonged length of hospital stay.

**Summary of rationale:** Hospital-associated sarcopenia develops in association with prolonged bed rest, inactivity, and malnutrition during hospitalization and has been observed in acute, perioperative, and convalescent settings. Estimates vary by population and diagnostic criteria, but early assessment and preventive intervention are important ^(226), (227), (228), (229), (230), (231), (232), (233), (234), (235), (236)^.

### Domain 11: Social Determinants of Health

#### FRQ2. Is screening for social determinants of health useful for improving outcomes in adults receiving rehabilitation treatment or exercise therapy?

**Statement:** Screening tools for assessing SDH are available. However, no evidence was identified regarding the usefulness of practices in which such tools were applied to adults receiving rehabilitation treatment or exercise therapy and linked to subsequent interventions. Further studies are needed to examine the usefulness of SDH screening. Therefore, no guideline recommendation is made for this item, and it is designated as a Future Research Question.

**Summary of rationale:** Validated SDH screening tools exist, but no evidence was identified evaluating their use linked to subsequent interventions in adults receiving rehabilitation treatment or exercise therapy. Future studies should evaluate outcomes and cost-effectiveness of interventions based on screening results ^(237), (238), (239), (240), (241), (242), (243)^.

#### FRQ3. Do interventions that take social determinants of health into account improve outcomes, including treatment continuation, activities of daily living, and healthcare costs, in adults receiving rehabilitation treatment or exercise therapy?

**Statement:** Interventions based on SDH, such as financial and transportation support, social support, telerehabilitation, and home environment modification, may have favorable effects on treatment continuation and healthcare costs in adults receiving rehabilitation treatment or exercise therapy. However, existing studies are limited in terms of sample size and methodological quality, and the evidence is insufficient to draw conclusions regarding the magnitude of effect or generalizability of these interventions. Therefore, no guideline recommendation is made for this item, and it is designated as a Future Research Question.

**Summary of rationale:** Evidence on SDH-informed interventions in adults receiving rehabilitation treatment or exercise therapy is limited. Because of the social nature of SDH, individual randomization is often difficult; future studies should clarify SDH components and intervention intensity using pragmatic trials, cluster RCTs, propensity score methods, and other appropriate designs ^(238), (244), (245), (246), (247), (248), (249), (250), (251), (252), (253), (254), (255)^.

## Article Information

### Author Contributions

Served as members of the guideline development organization for the Japanese primary guideline and were fully involved in the guideline development process in accordance with standard clinical practice guideline development methods: All authors. Made substantial contributions to the conception and design of the guideline, determination of the scope, formulation and review of the clinical questions, background questions, and future research questions, consideration of the evidence summaries, deliberation and approval of the statements, and review of the recommendation strengths and certainty ratings: All authors. Contributed to the preparation or critical revision of this English executive summary for important intellectual content, approved the final version for submission, and agree to be accountable for all aspects of the work: All authors.

### Conflicts of Interest

All authors completed disclosures of financial conflicts of interest and non-financial academic interests for the Japanese primary guideline. Conflicts of interest and relevant non-financial academic interests were managed in accordance with the regulations of the Japanese Association of Rehabilitation Nutrition and Minds guidance, including restriction of voting rights for relevant clinical questions when applicable. Hidetaka Wakabayashi received lecture fees from Otsuka Pharmaceutical Factory, Inc. Hideki Aragane reported a financial conflict of interest concerning stock or profit interests of a spouse, first-degree relative, or person sharing income or assets, related to Takeda Pharmaceutical Company Limited. Yoshihiro Yoshimura, Shinta Nishioka, Ai Shiraishi, Yoko Saino, Ayaka Matsumoto, and Hidetaka Wakabayashi reported non-financial academic interests through leadership roles in academic societies and/or involvement in related clinical practice guidelines. All other authors declare no conflicts of interest.

### Secondary Publication Statement

This manuscript is an English executive summary and secondary publication of the Japanese Association of Rehabilitation Nutrition Clinical Practice Guideline 2026. It is based on the Japanese primary publication and does not add new clinical questions, background questions, future research questions, statements, evidence, interpretations, or figure/table concepts beyond the primary guideline.

### Primary Publication and Permission Statement

The Japanese primary publication is: Yasumoto Y, Matsumoto T, Nagano A, et al. Clinical Practice Guideline for Rehabilitation Nutrition 2026. Journal of Japanese Association of Rehabilitation Nutrition. 2026;10(1):110–190.

Primary publication URL: https://www.ishiyaku.co.jp/search/details?bookcode=265380

The Japanese primary guideline has already been published in Japanese. Permission to submit this English secondary publication to *JMA Journal* has been obtained from the primary publication venue and *JMA Journal*.

The Japanese primary publication is included in the manuscript reference list and cited in the main text. The manuscript title explicitly identifies this article as a secondary publication.

## Supplement

Supplementary Material **Supplementary Figure 1-1.** Literature search flow diagram (PRISMA 2020): patients with cerebrovascular disease in the acute phase.
PRISMA: Preferred Reporting Items for Systematic Reviews and Meta-Analyses.
**Supplementary Figure 2-1.** Literature search flow diagram (PRISMA 2020): patients with cerebrovascular disease in the convalescent phase.
PRISMA: Preferred Reporting Items for Systematic Reviews and Meta-Analyses.
**Supplementary Figure 3-1.** Literature search flow diagram (PRISMA 2020): patients with proximal femoral fracture.
PRISMA: Preferred Reporting Items for Systematic Reviews and Meta-Analyses.
**Supplementary Figure 4-1**. Literature search flow diagram (PRISMA 2020): patients with COPD.
*The 22 reports included in previously reported systematic reviews were rechecked at the eligibility-assessment stage and were not identified through the new search.
COPD: chronic obstructive pulmonary disease; PRISMA: Preferred Reporting Items for Systematic Reviews and Meta-Analyses.
**Supplementary Figure 5-1.** Literature search flow diagram (PRISMA 2020): patients with cancer.
PRISMA: Preferred Reporting Items for Systematic Reviews and Meta-Analyses.
**Supplementary Figure 6-1.** Literature search flow diagram (PRISMA 2020): exercise therapy for patients with dysphagia.
PRISMA: Preferred Reporting Items for Systematic Reviews and Meta-Analyses.
**Supplementary Figure 7-1.** Literature search flow diagram (PRISMA 2020): nutritional therapy for patients with dysphagia.
PRISMA: Preferred Reporting Items for Systematic Reviews and Meta-Analyses.
**Supplementary Figure 8-1.** Literature search flow diagram (PRISMA 2020): malnutrition in patients with dysphagia.
PRISMA: Preferred Reporting Items for Systematic Reviews and Meta-Analyses,
**Supplementary Figure 9-1.** Literature search flow diagram (PRISMA 2020): exercise therapy for patients with dysphagia.
PRISMA: Preferred Reporting Items for Systematic Reviews and Meta-Analyses.
**Supplementary Figure 10-1.** Literature search flow diagram (PRISMA 2020): exercise therapy and nutritional therapy for patients with dysphagia.
PRISMA: Preferred Reporting Items for Systematic Reviews and Meta-Analyses.
**Supplementary Figure 11-1.** Literature search flow diagram (PRISMA 2020): nutritional therapy for patients with dysphagia, including enteral nutrition, parenteral nutrition, oral nutritional supplementation, and nutritional counseling.
PRISMA: Preferred Reporting Items for Systematic Reviews and Meta-Analyses.
**Supplementary Figure 12-1.** Literature search flow diagram (PRISMA 2020): intensive exercise therapy for patients with malnutrition.
PRISMA: Preferred Reporting Items for Systematic Reviews and Meta-Analyses.
**Supplementary Figure 14-1.** Literature search flow diagram (PRISMA 2020): nutrition assessment by registered dietitians for older adults, older adults requiring long-term care, and frail older adults.
PRISMA: Preferred Reporting Items for Systematic Reviews and Meta-Analyses.
**Supplementary Figure 15-1.** Literature search flow diagram (PRISMA 2020): nutritional counseling by registered dietitians for older adults, older adults requiring long-term care, and frail older adults.
PRISMA: Preferred Reporting Items for Systematic Reviews and Meta-Analyses.
**Supplementary Figure 16-1.** Literature search flow diagram (PRISMA 2020): professional oral management.
PRISMA: Preferred Reporting Items for Systematic Reviews and Meta-Analyses.
**Supplementary Figure 17-1.** Literature search flow diagram (PRISMA 2020): pharmacist-led pharmaceutical interventions.
PRISMA: Preferred Reporting Items for Systematic Reviews and Meta-Analyses.
**Supplementary Figure 18-1.** Literature search flow diagram (PRISMA 2020): hospital-associated sarcopenia.
PRISMA: Preferred Reporting Items for Systematic Reviews and Meta-Analyses.
**Supplementary Figure 19-1.** Literature search flow diagram (PRISMA 2020): screening for SDH.
PRISMA: Preferred Reporting Items for Systematic Reviews and Meta-Analyses; SDH: social determinants of health.
**Supplementary Figure 20-1.** Literature search flow diagram (PRISMA 2020): interventions considering SDH.
PRISMA: Preferred Reporting Items for Systematic Reviews and Meta-Analyses; SDH: social determinants of health.
