# Supplementary material for: Clinical Practice Guideline for Rehabilitation Nutrition 2026: Executive Summary of the Japanese Association of Rehabilitation Nutrition Guideline 2026—A Secondary Publication
Source: JMA J. 2026 Aug 14;9(5):1360–78. doi: 10.31662/jmaj.2026-0293 (PMC13639588; doi:10.31662/jmaj.2026-0293)
Supplement: Supplementary file 1 [file 2433-3298-9-5-1360-s001.pdf]

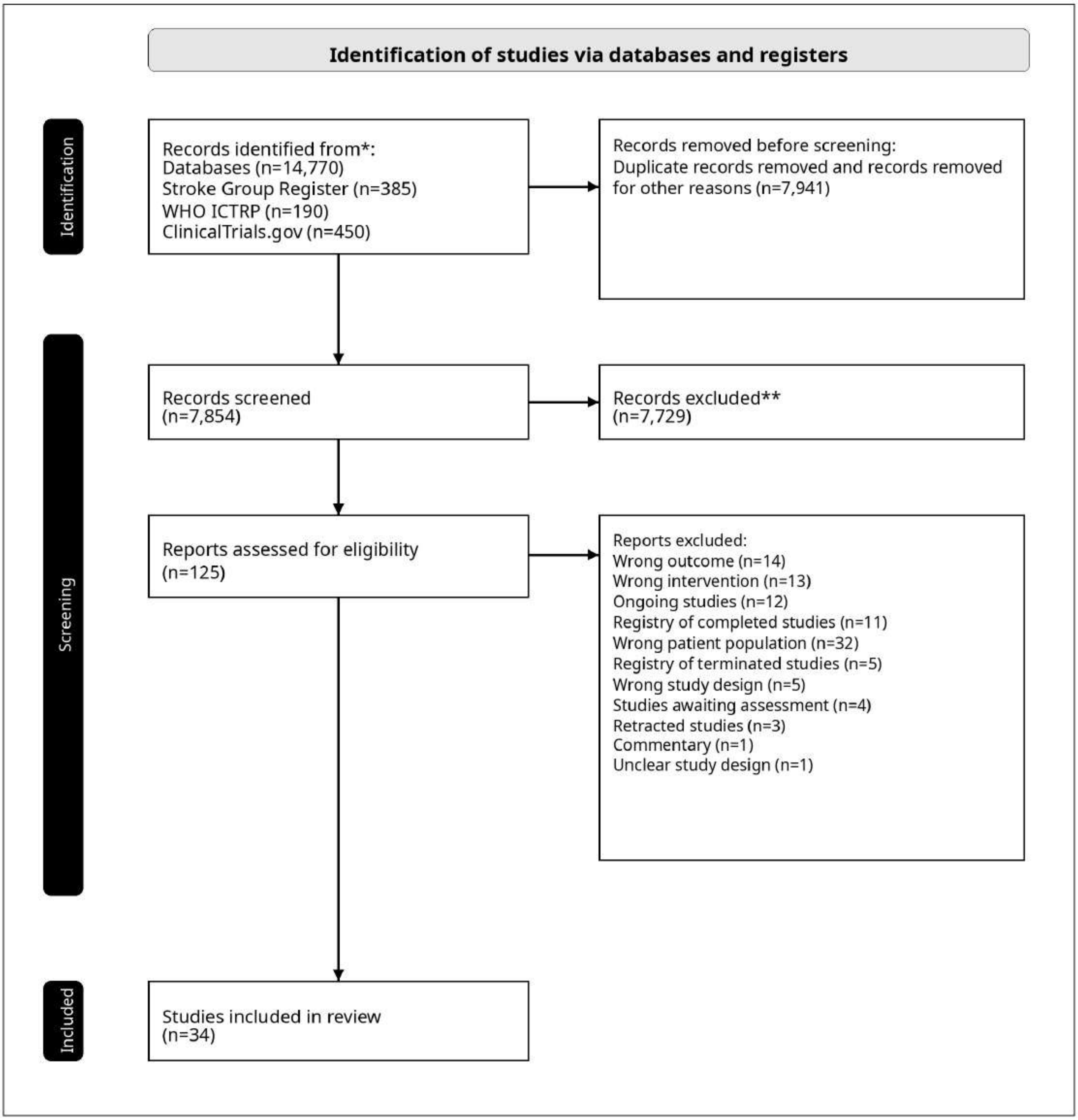

Supplementary Figure 1-1. Literature search flow diagram (PRISMA 2020): Patients with acute cerebrovascular disease

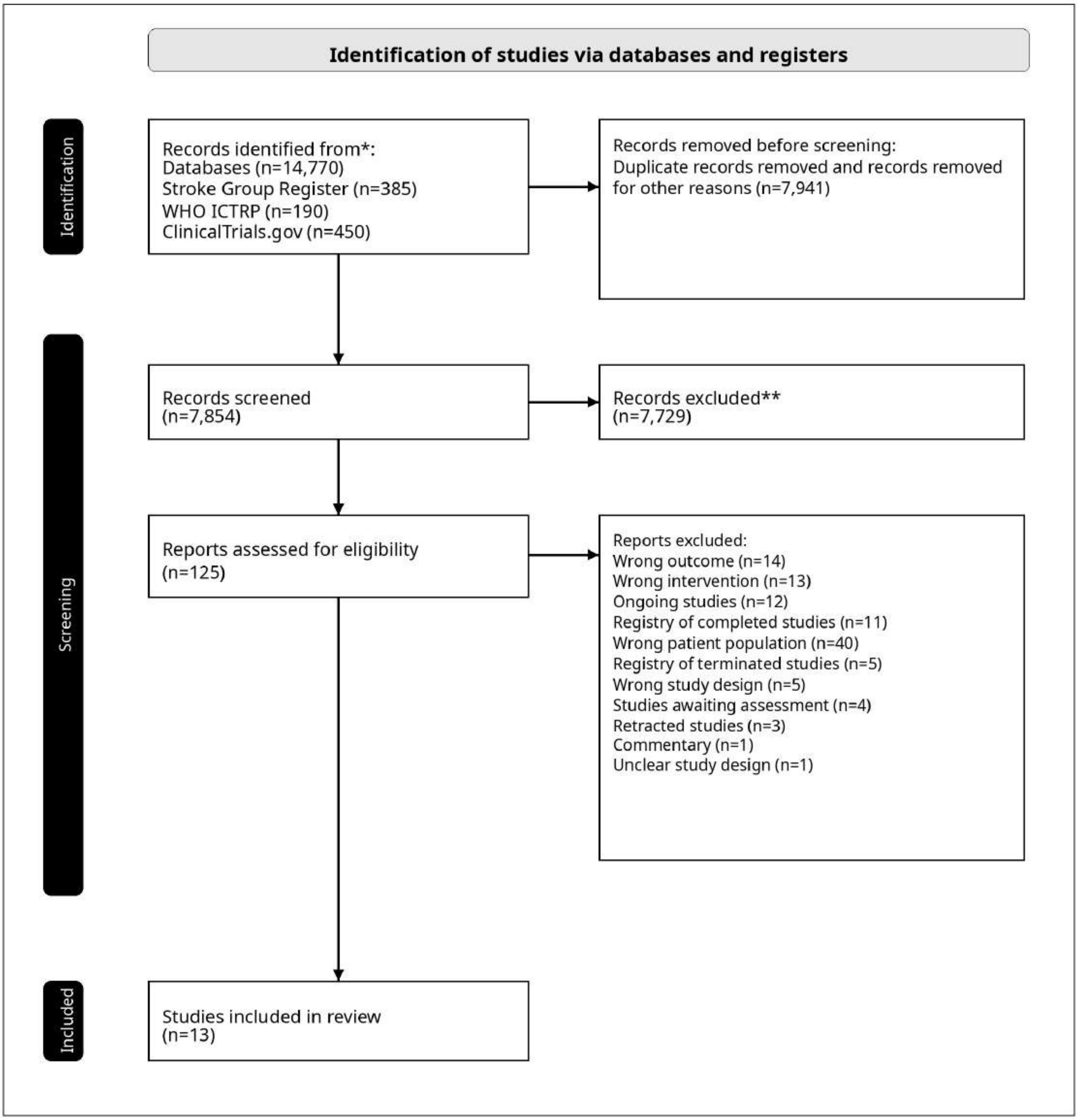

Supplementary Figure 2-1. Literature search flow diagram (PRISMA 2020): Patients with cerebrovascular disease in the convalescent phase

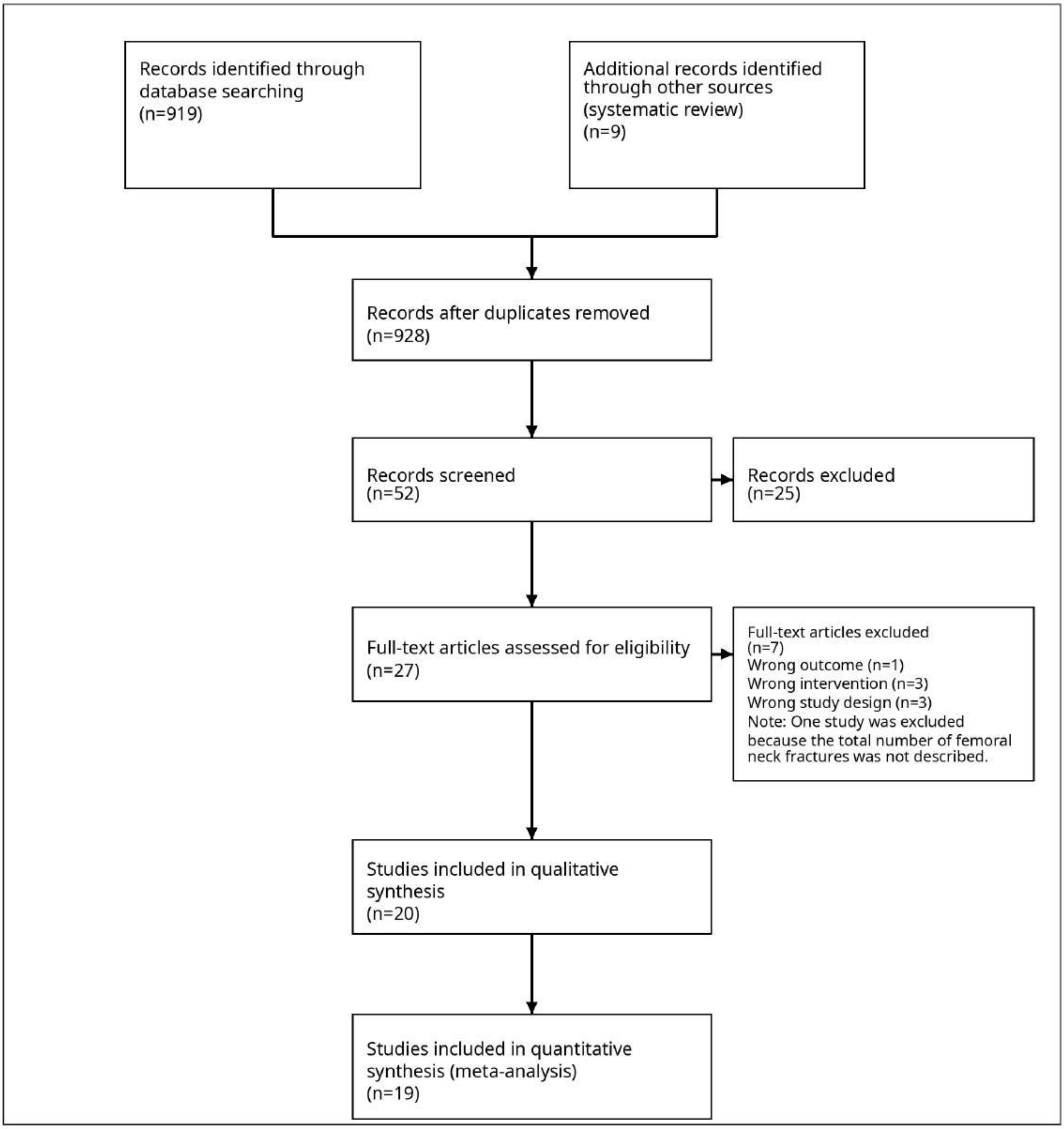

Supplementary Figure 3-1. Literature search flow diagram (PRISMA 2020): Patients with proximal femoral fracture

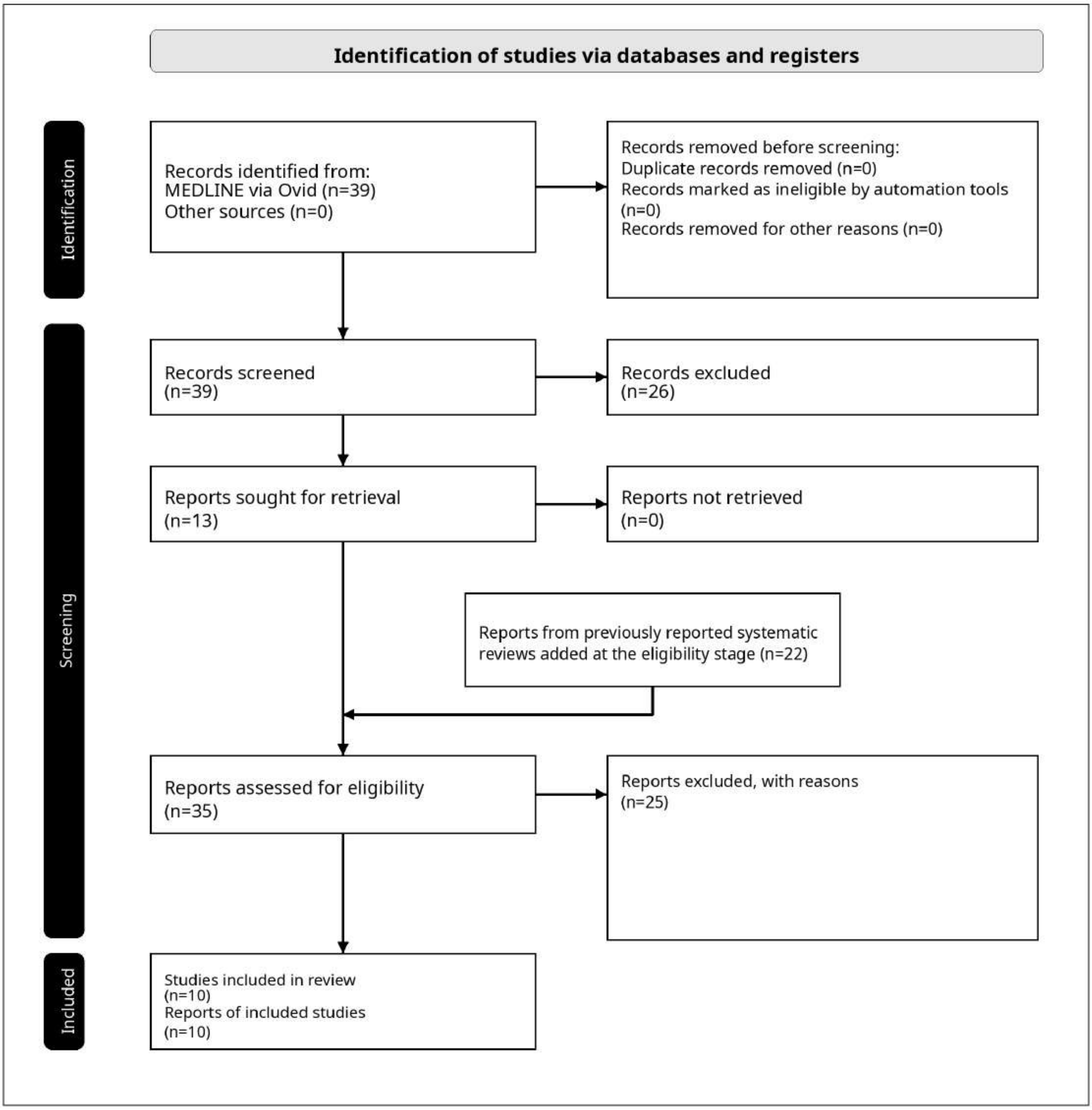

Supplementary Figure 4-1. Literature search flow diagram (PRISMA 2020): Patients with COPD

* The 22 reports included in previously reported systematic reviews were rechecked at the eligibility stage and were not identified by the new search.

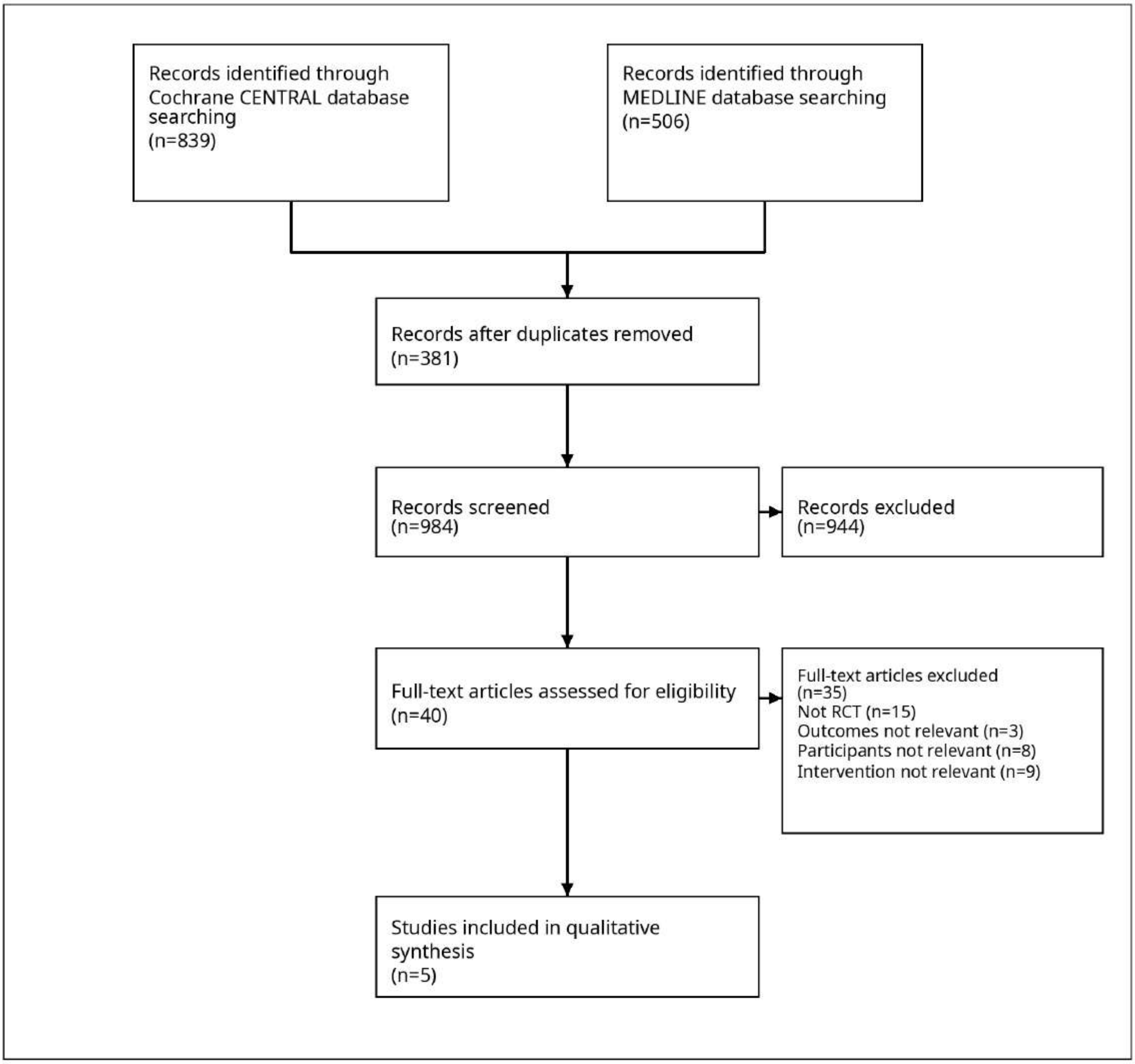

Supplementary Figure 5-1. Literature search flow diagram (PRISMA 2020): Patients with cancer

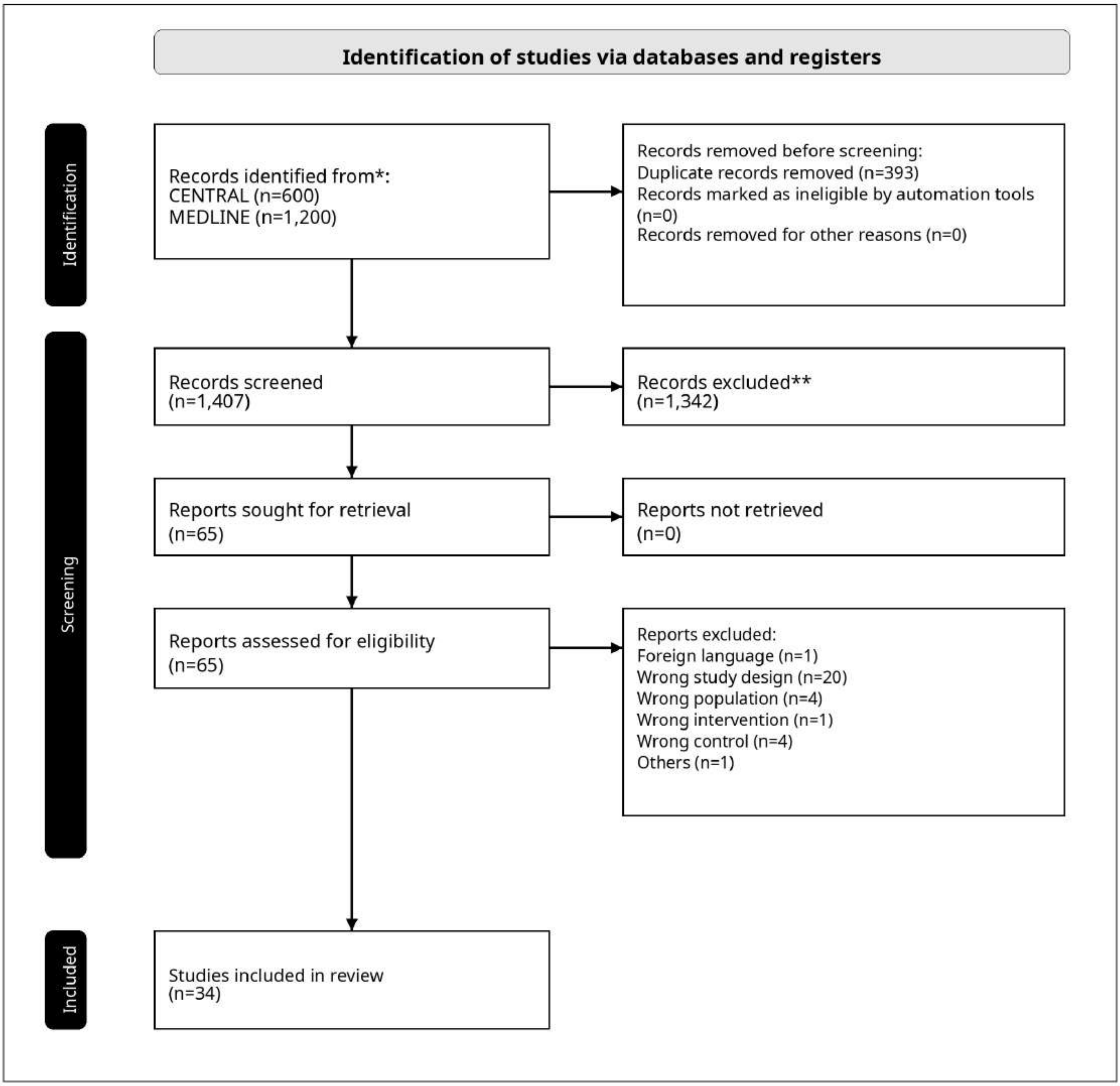

Supplementary Figure 6-1. Literature search flow diagram (PRISMA 2020): Exercise therapy for patients with dysphagia

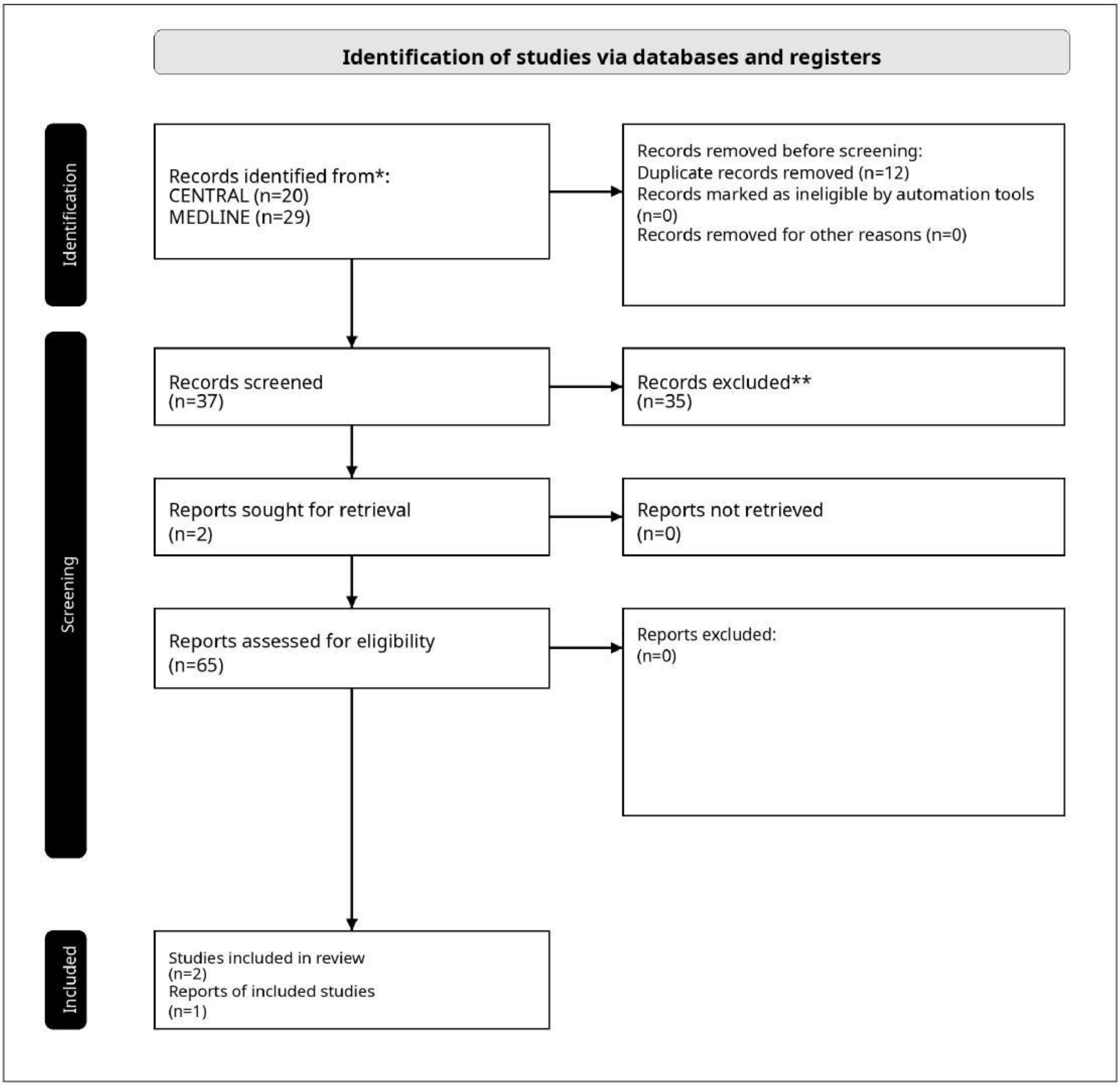

Supplementary Figure 7-1. Literature search flow diagram (PRISMA 2020): Nutritional therapy for patients with dysphagia

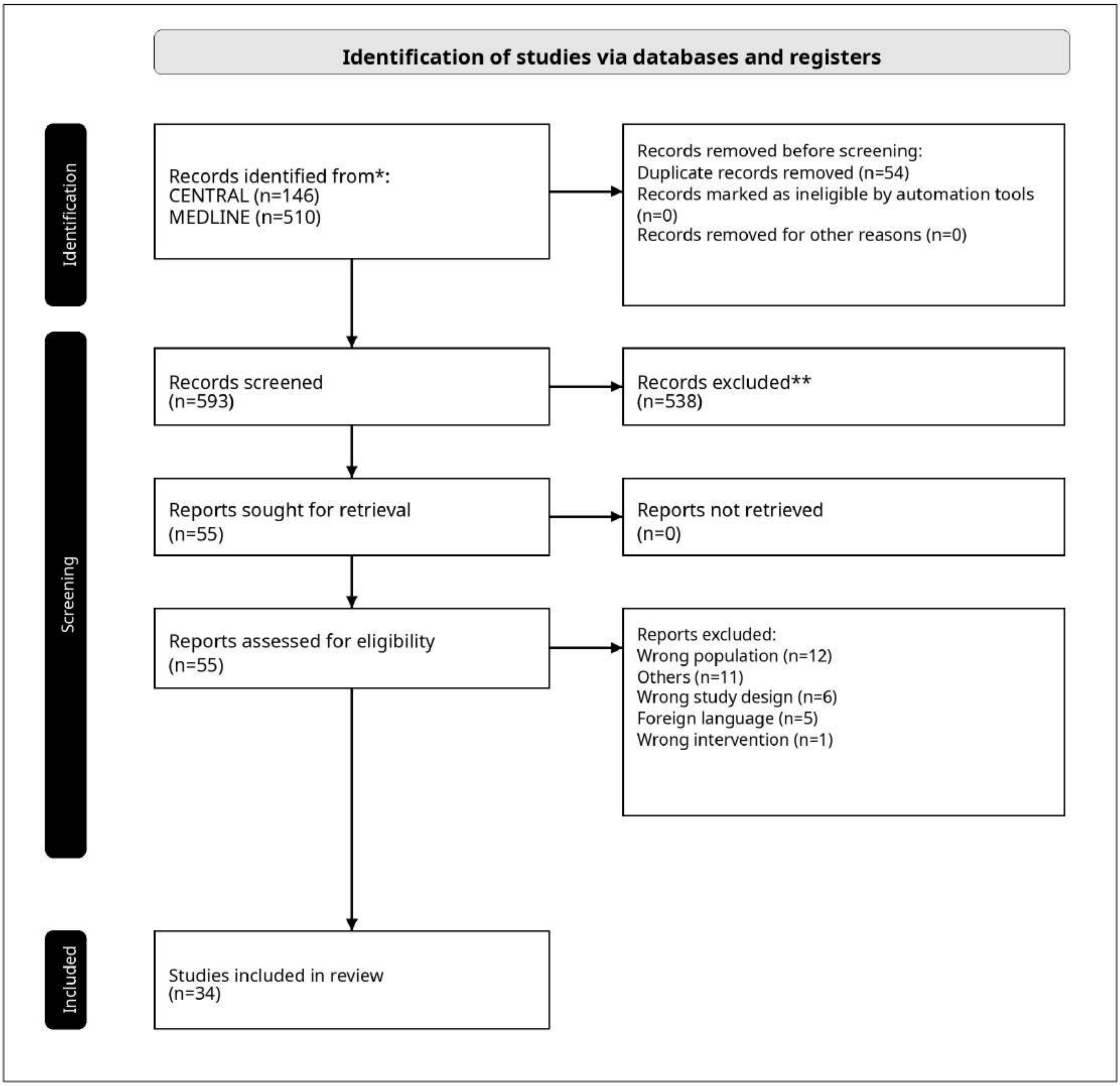

Supplementary Figure 8-1. Literature search flow diagram (PRISMA 2020): Malnutrition in patients with dysphagia

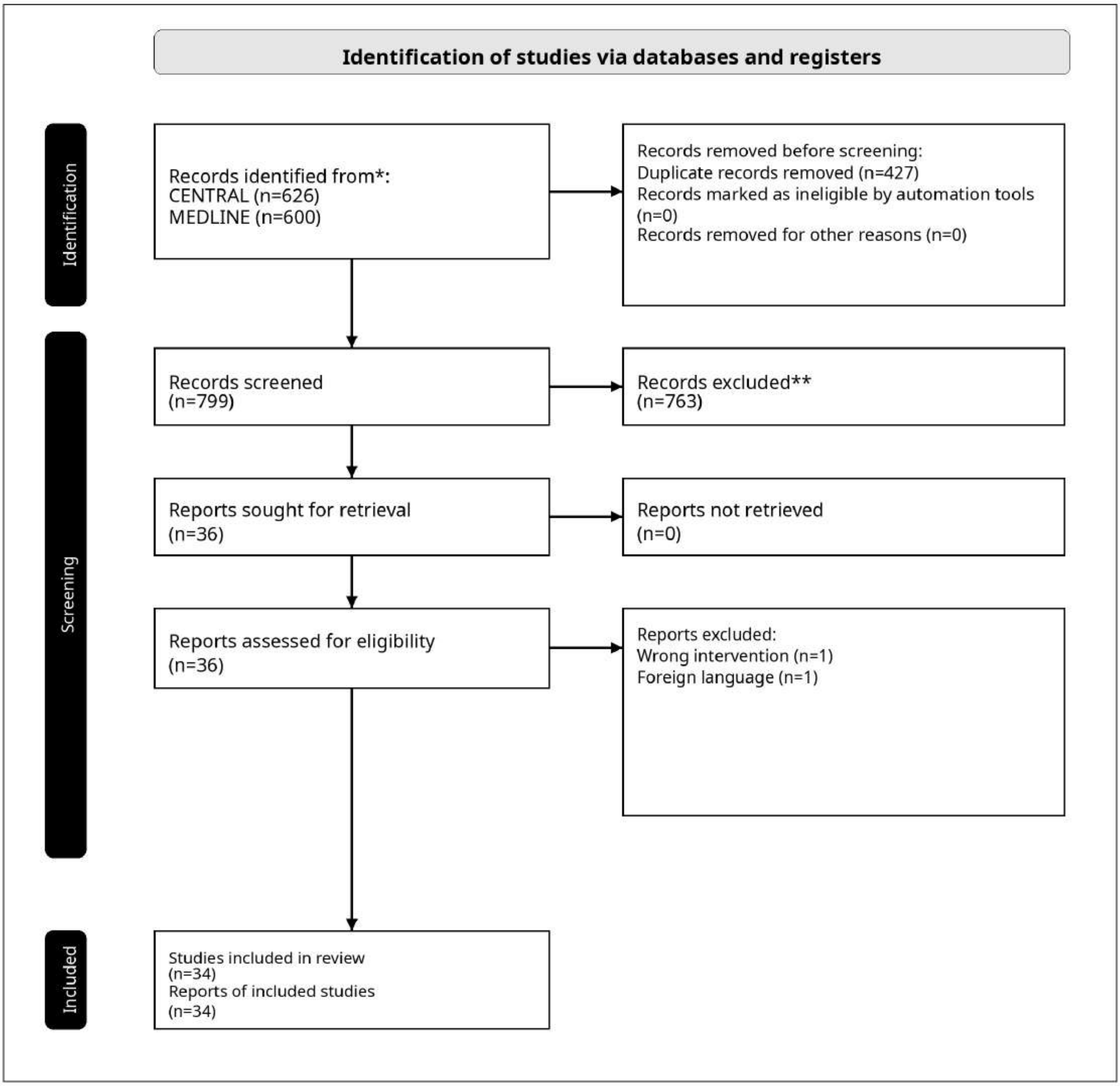

Supplementary Figure 9-1. Literature search flow diagram (PRISMA 2020): Exercise therapy for patients with dysphagia

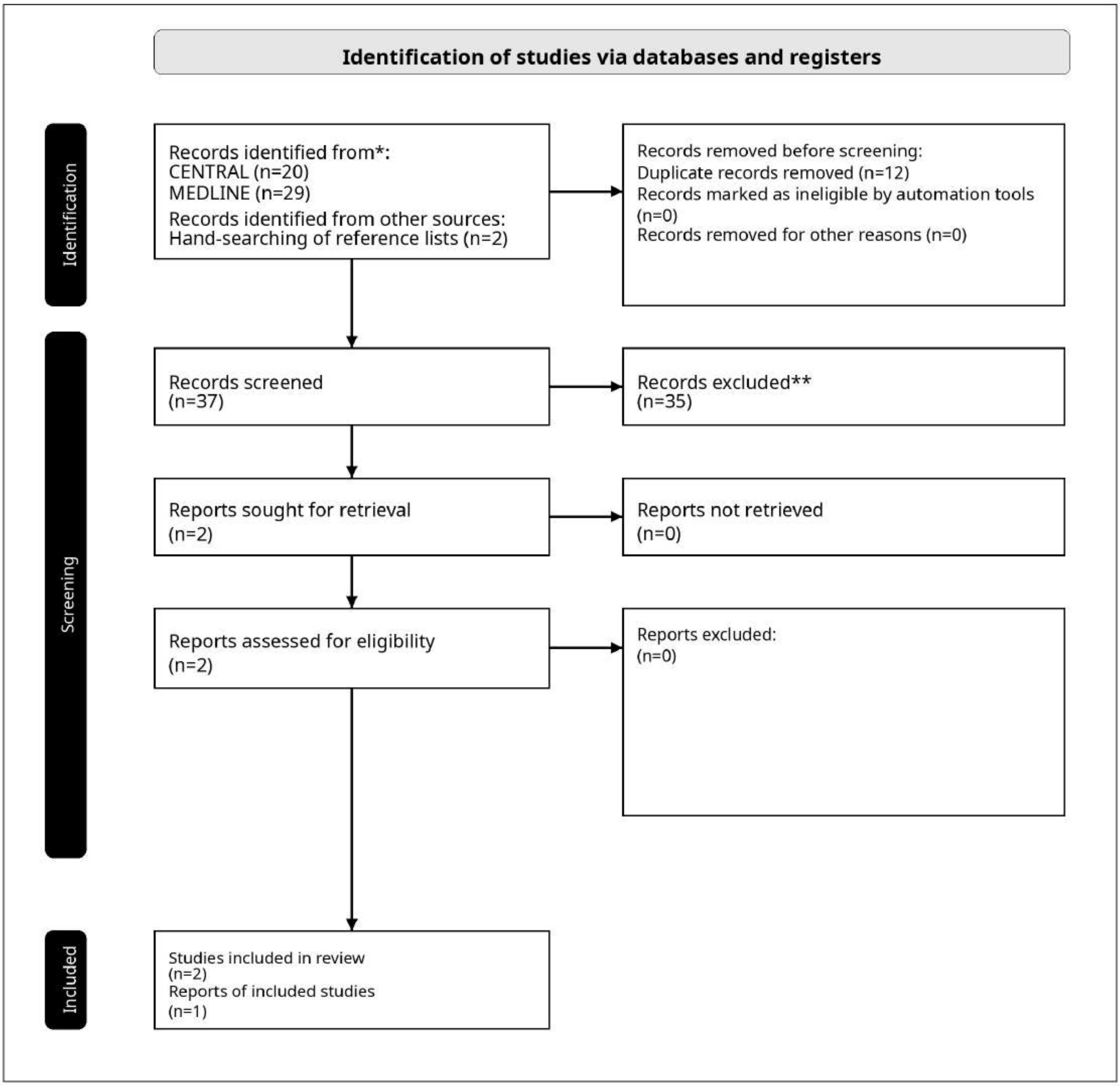

Supplementary Figure 10-1. Literature search flow diagram (PRISMA 2020): Exercise therapy and nutritional therapy for patients with dysphagia

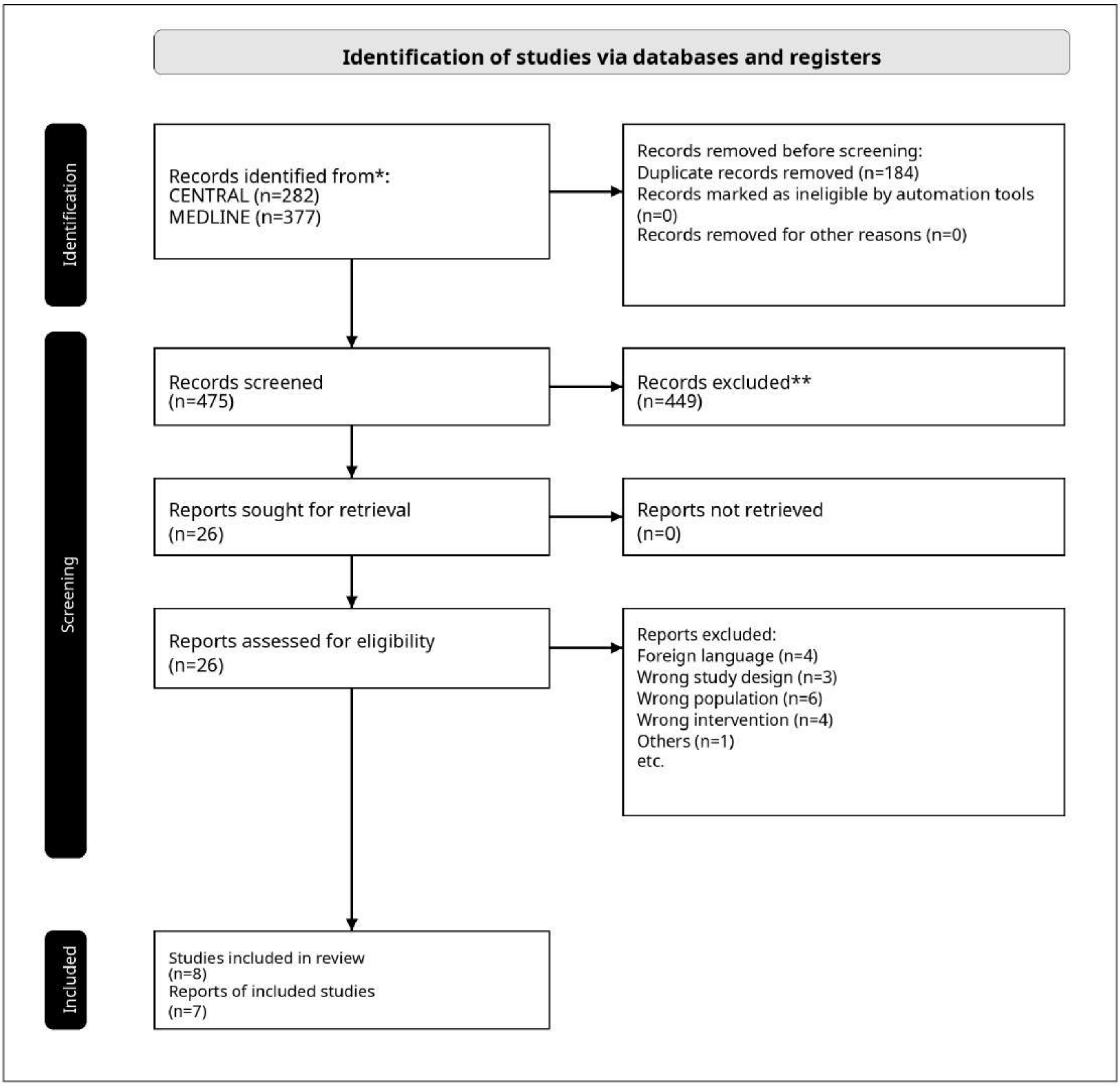

Supplementary Figure 11-1. Literature search flow diagram (PRISMA 2020): Nutritional therapy for patients with dysphagia (enteral nutrition, parenteral nutrition, provision of oral nutritional supplements, and nutritional guidance)

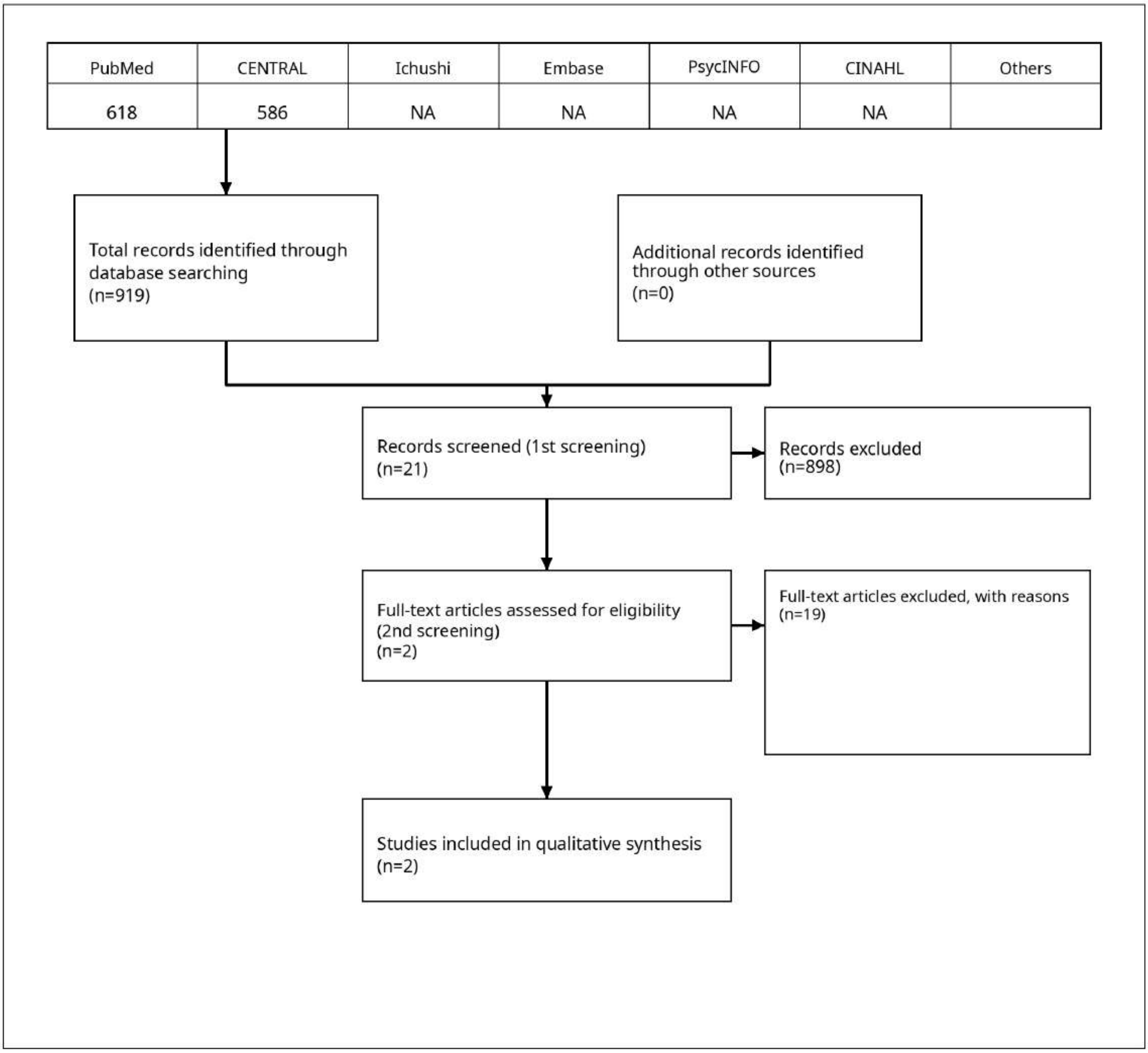

Supplementary Figure 12-1. Literature search flow diagram (PRISMA 2020): Intensive exercise therapy for patients with malnutrition

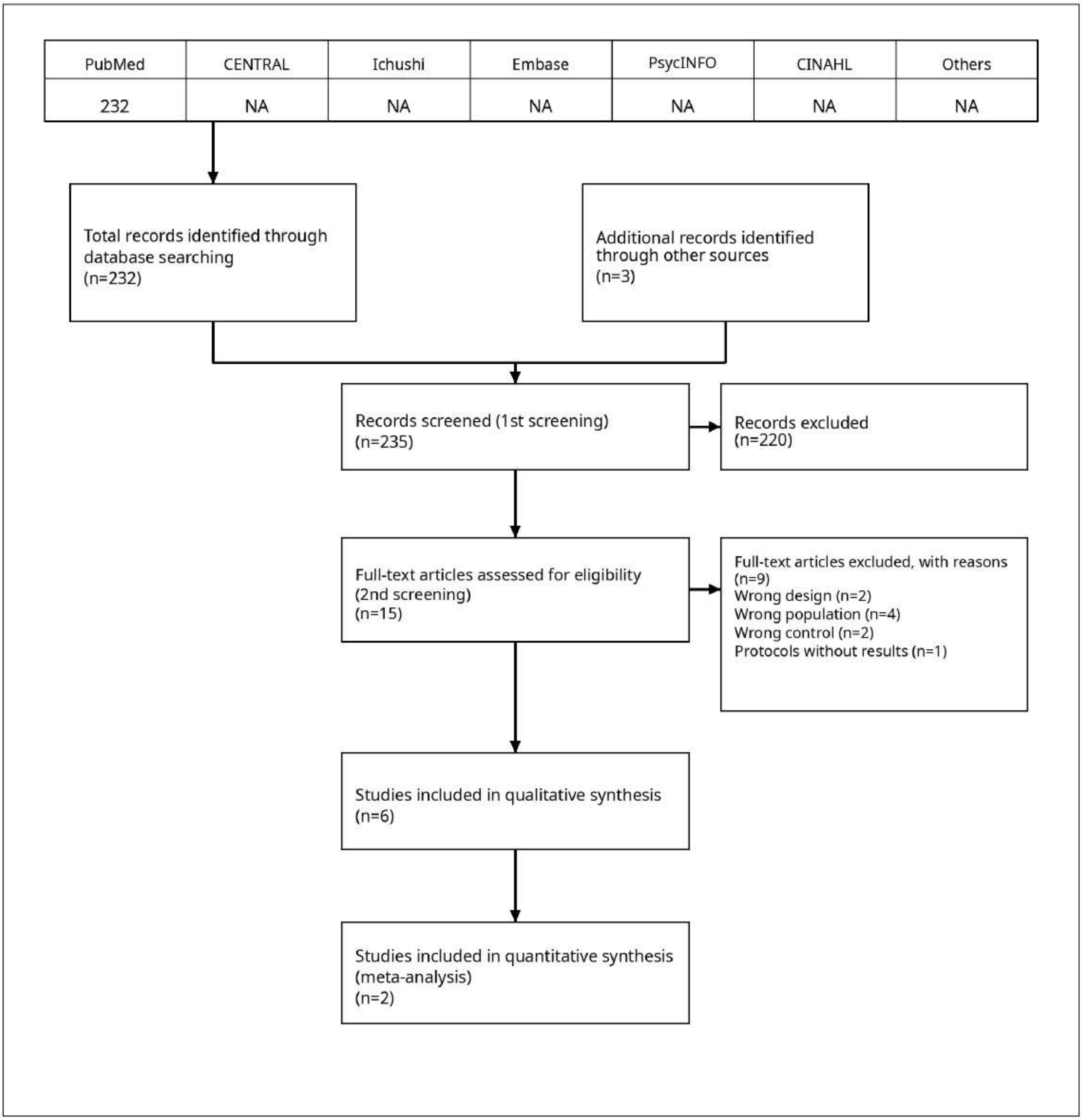

Supplementary Figure 14-1. Literature search flow diagram (PRISMA 2020): Nutritional assessment by registered dietitians for older adults, older adults requiring long-term care, and frail older adults

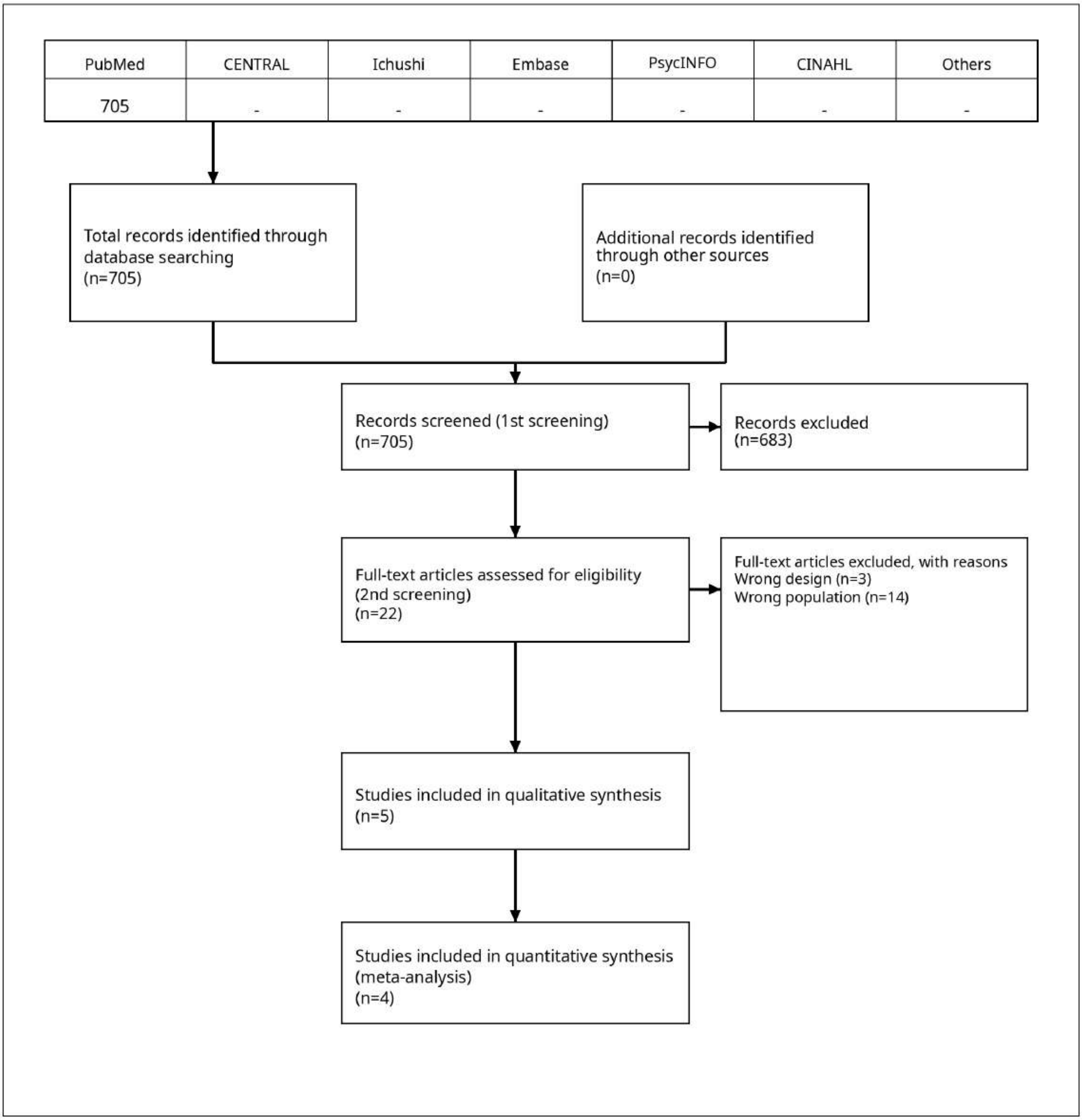

Supplementary Figure 15-1. Literature search flow diagram (PRISMA 2020): Nutritional guidance by registered dietitians for older adults, older adults requiring long-term care, and frail older adults

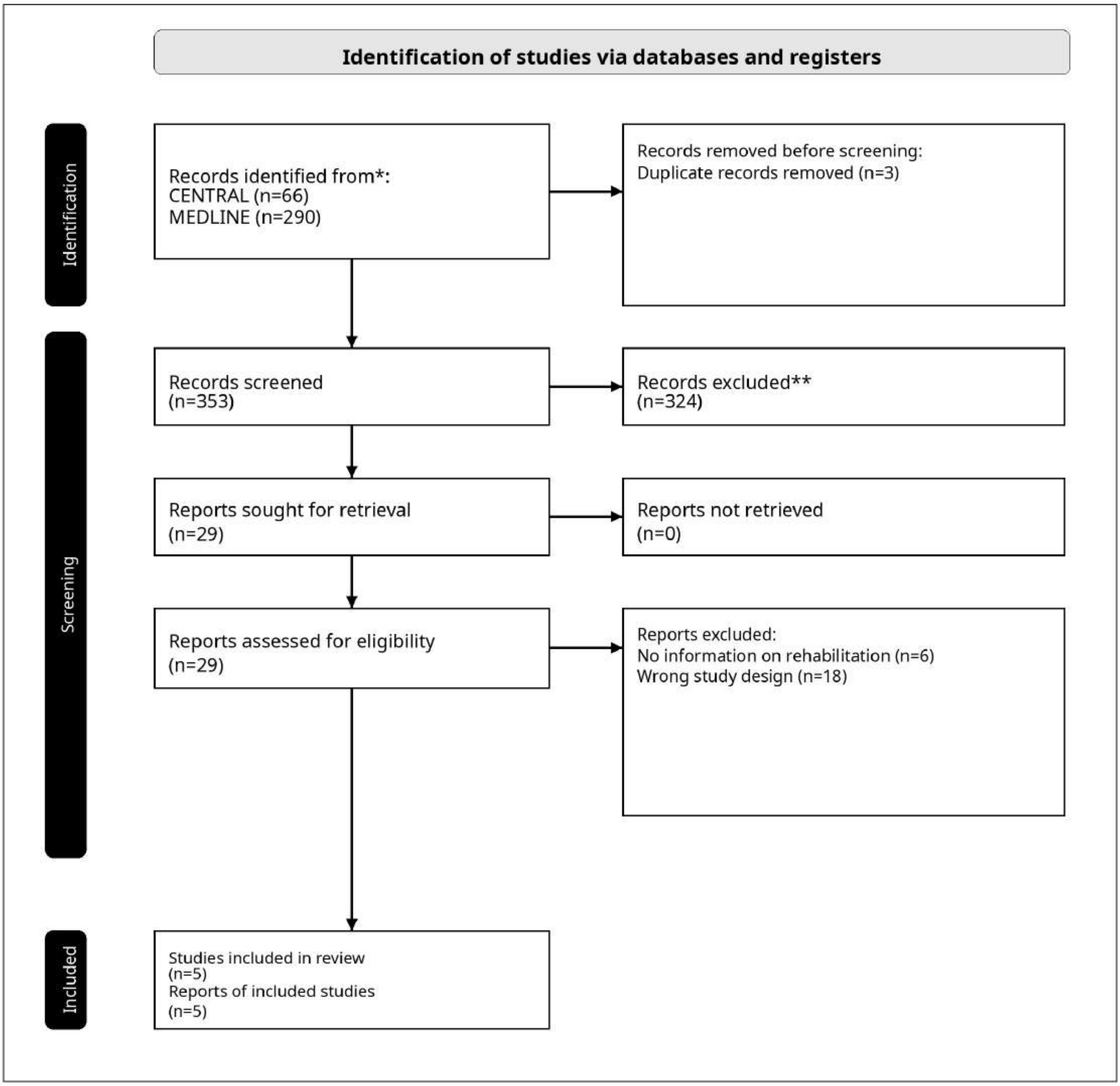

Supplementary Figure 16-1. Literature search flow diagram (PRISMA 2020): Professional oral management

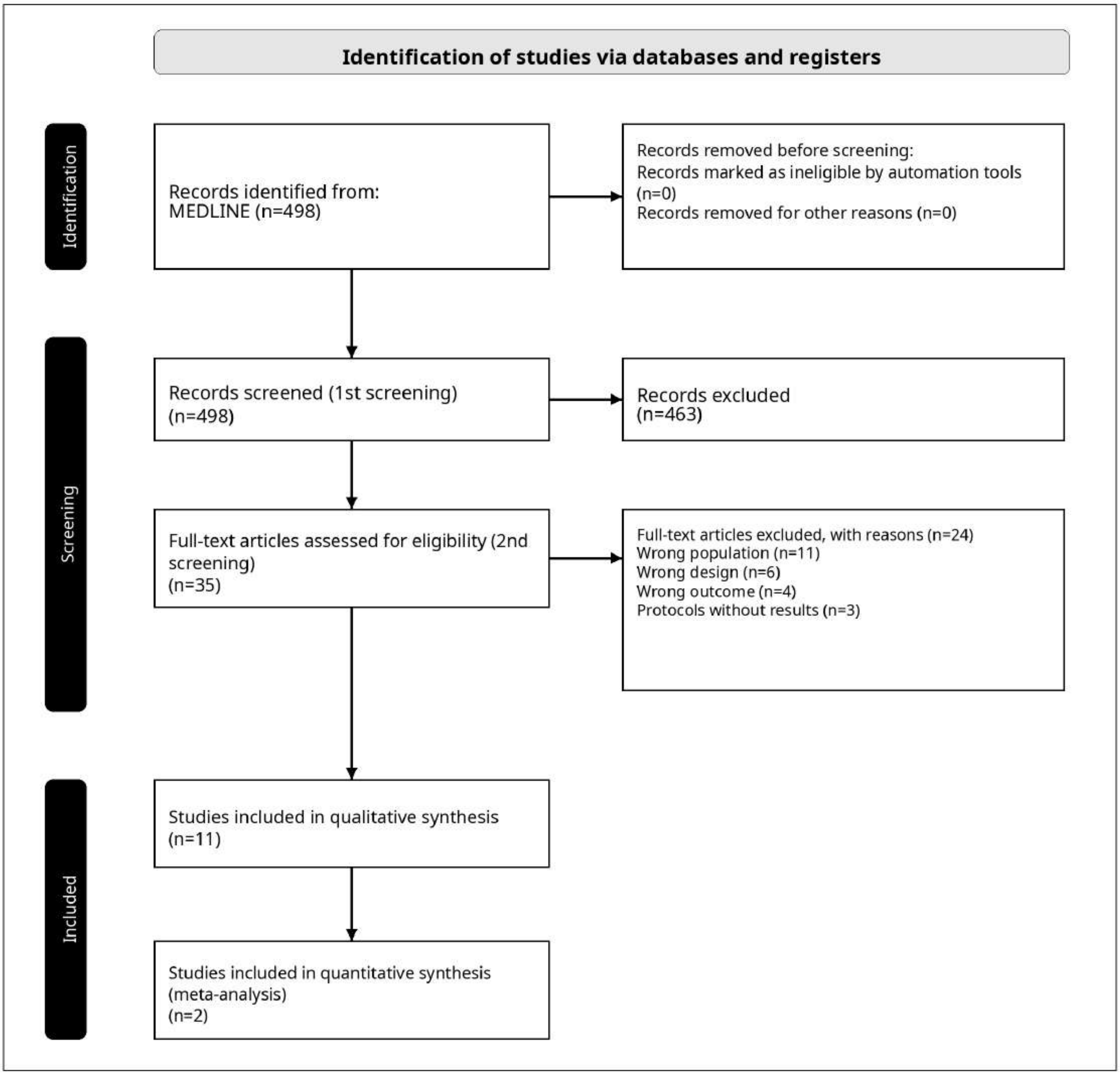

Supplementary Figure 17-1. Literature search flow diagram (PRISMA 2020): Pharmacist-led pharmaceutical intervention

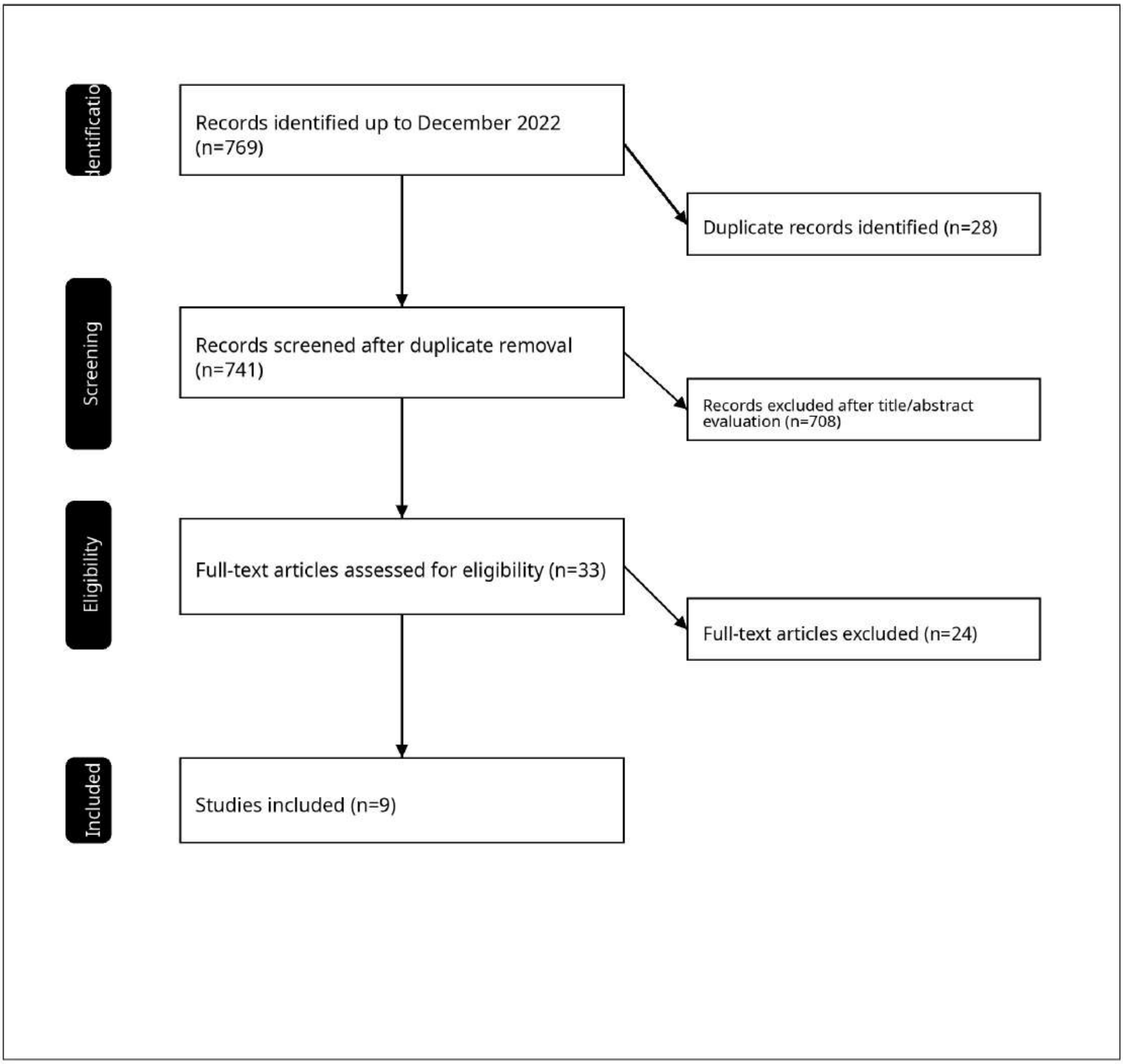

Supplementary Figure 18-1. Literature search flow diagram (PRISMA 2020): Hospital-associated sarcopenia

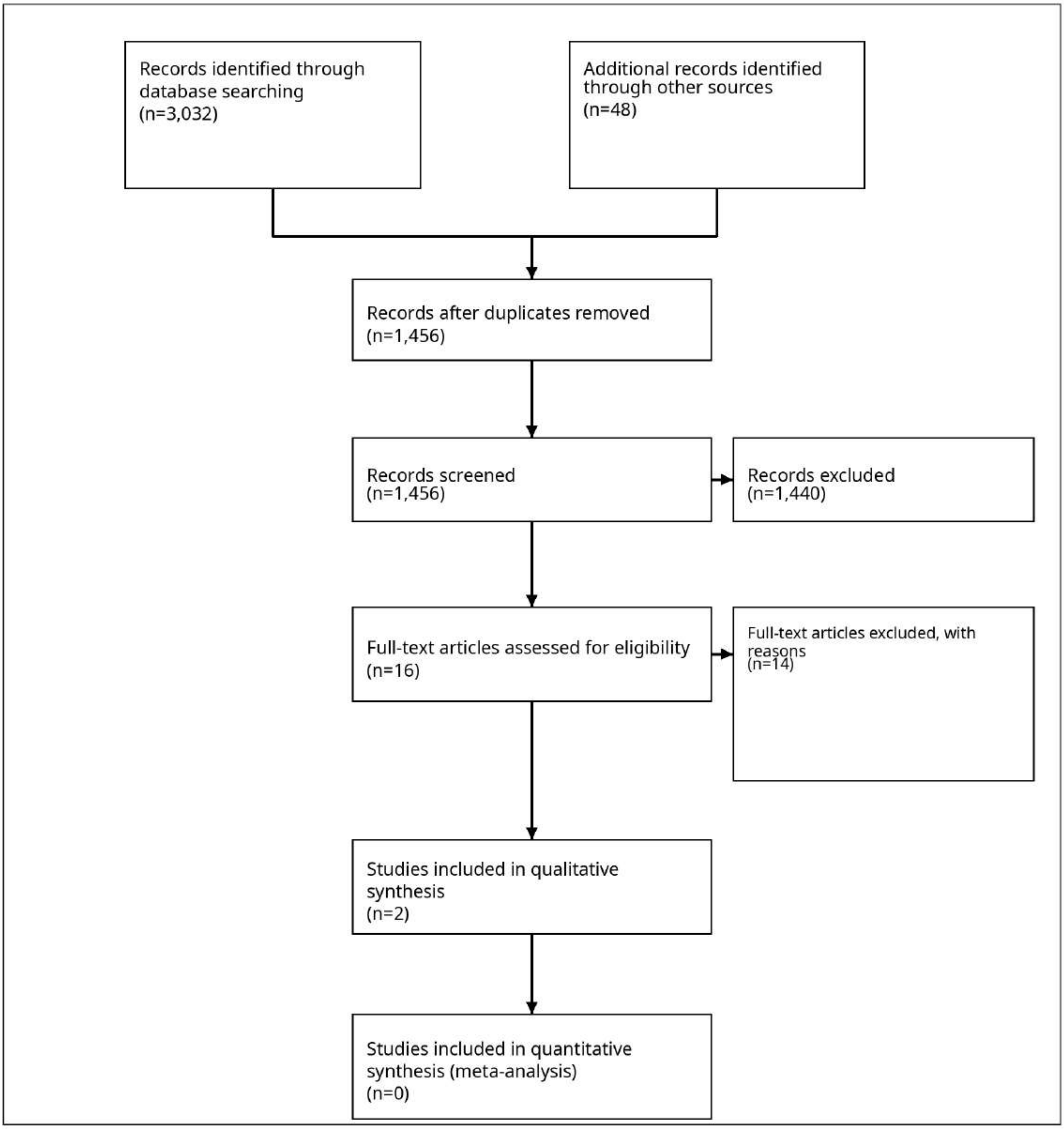

Supplementary Figure 19-1. Literature search flow diagram (PRISMA 2020): Screening for social determinants of health (SDH)

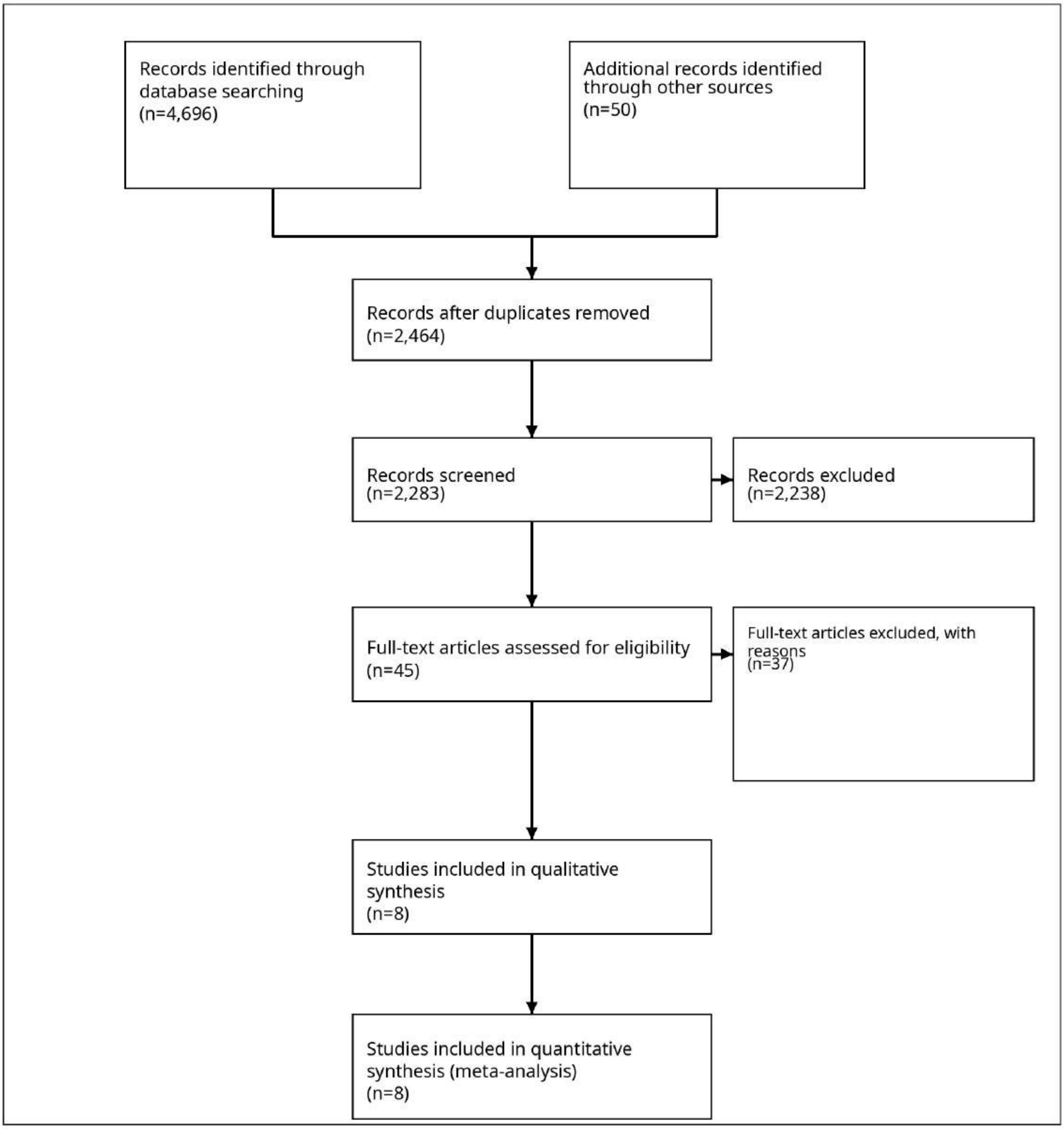

Supplementary Figure 20-1. Literature search flow diagram (PRISMA 2020): Interventions considering social determinants of health (SDH)

Supplementary Table 1-1. Structured abstracts (CQ1)

Author, year	N	Intervention period	Intervention	Control	Outcomes	Findings
Badjatia et al, 2021	26	14 days	Protein 1.75 g/kg (with at least leucine 3 g) plus electrical stimulation	Usual diet (protein 1.2-1.4 g/kg/day)	Muscle mass at day 90; physical function	The muscle atrophy rate was significantly lower in the intervention group than in the control group (6.5 +/- 4.1% vs 12.5 +/- 6.4%, P=0.01). The intervention group had a higher Short Physical Performance Battery (SPPB) score at day 90 (12 [10, 12] vs 9 [4, 12], P=0.01).
Berger et al, 2008	21	5 days	Zinc plus vitamin B, vitamin C, and vitamin E	Usual diet (vitamin C 500 mg)	Complications	No additional finding was described in the source table.
Daga et al, 1997	27	15 days	Vitamin E 300 mg	Placebo	Barthel Index at day 15 and 6 weeks	No significant difference in BI was observed at the end of intervention or at 6 weeks.
Das et al, 2021	12	Not reported	Individualized nutrition therapy (enteral)	Usual care	ICU mortality	There was no significant difference in ICU mortality between groups.
de Aguilar-Nascimento et al, 2011	31	5 days	Whey protein 1.2 kg/kg and 35 kcal/kg (enteral)	Casein protein 1.2 kg/kg and 35 kcal/kg	ICU mortality	There was no difference in ICU mortality (whey protein: 30%; casein protein: 26.7%; P=1.00).
Dennis et al, 2005a	4,023	Until discharge	Energy- and protein-enriched oral nutrition (360 mL; 6.27 kJ/mL; protein 62.5 g/L)	Usual diet	Mortality; complications; QoL	The mortality risk difference decreased by 0.7% in the intervention group (95% CI: 1.4 to 2.7). During intervention, diarrhea was significantly more frequent (OR 4.29, 95% CI: 1.98-9.28), and hyperglycemia or hypoglycemia was also significantly more frequent (OR 15.6, 95% CI: 4.84-50.23). There were no significant differences in complications such as pneumonia or urinary tract infection. QoL (EQ-5D) did not differ significantly.
Dennis et al, 2005b	859	Not applicable	Early enteral nutrition	Parenteral nutrition; tube feeding started after day 7	Death; complications	Mortality decreased by 5.8% in the early enteral nutrition group (95% CI: -0.8% to 12.5%, P=0.09), but the difference was not significant. There were no significant differences in diarrhea, pneumonia, or pressure ulcers. QoL tended to be lower in the intervention group.
Gao et al, 2008	60	During hospitalization	High-protein enteral nutrition; PFC ratio 20:45:35	Low-protein enteral nutrition; PFC ratio 15:53:32	Mortality	There was no significant difference between groups (intervention 10.3%, control 15.4%).
Gariballa et al, 1998	42	4 weeks or until death/discharge	Energy- and protein-enriched supplement (600 kcal, 20 g)	Usual diet	Death; BI	BI showed similar trends at baseline (intervention median 45, IQR 20 to 58.75; control median 35, IQR 16.25 to 48.75) and at assessment (intervention median 90, IQR 60 to 93.75; control median 75, IQR 47 to 87.5). Mortality tended to be lower in the intervention group (10% vs 35%, P=0.127).
Gupta et al, 2016	53	6 months	Vitamin D (60,000 IU once monthly)	Usual diet	Death at 6 months	No intervention effect was observed (OR 0.29, 95% CI: 0.08 to 1.0).

Author, year	N	Intervention period	Intervention	Control	Outcomes	Findings
Ha et al, 2010a	170	During hospitalization (mean 12 days)	Individualized nutrition therapy (oral and enteral)	Usual diet	Death at 3 months; QoL	No effect on all-cause death was observed (OR 0.92, 95% CI: 0.49 to 1.17). In EQ-5D-based QoL assessment, only the EQ-5D VAS score showed a significant intervention effect.
Ha et al, 2010b	170	During hospitalization	Individualized nutrition therapy (oral and enteral)	Usual diet	Muscle mass	No difference was found in mid-arm muscle circumference at the end of the intervention.
Hashemilar et al, 2020	42	3 weeks	Protein-enriched oral nutrition	Usual diet	NIHSS	There was no significant difference between groups in the change in NIHSS score during the intervention (MD 0.58, 95% CI: 1.73 to 0.57).
Kadri et al, 2020	120	12 weeks	1) Vitamin A; 2) vitamin D; 3) vitamins A + D	Placebo	NIHSS	Among the interventions, the vitamin A + D group showed the greatest improvement in NIHSS score (vitamin A: 10.30 +/- 1.59; vitamin D: 10.40 +/- 1.23; vitamins A and D: 6.00 +/- 1.52; placebo: 11.75 +/- 1.29).
Laviano et al, 2011	20	14 days	Amino acid-enriched nutrition (enteral and intravenous)	Standard protein	Death; complications	No significant difference in mortality was observed (intervention 60%, control 77%). No significant difference was observed in urinary tract infection or blood glucose level at the end of intervention.
Li et al, 2008	54	21 days	Energy-enriched enteral nutrition	Standard enteral nutrition	ADL; complications	ADL at 30 days was significantly higher in the intervention group (MD 10.68, 95% CI: 6.35-15.01). No significant differences were observed in pneumonia or urinary tract infection.
Li et al, 2014	148	21 days	Energy- and protein-enriched enteral nutrition	Standard enteral nutrition	Death; complications	Mortality differed significantly (intervention 15.3%, control 21.2%). Pneumonia incidence was significantly lower in the intervention group (41.3% vs 63.2%).
Li et al, 2016	112	1 month	Early enteral nutrition within 48 hours, gradually increased to 2,000 kcal	Parenteral nutrition	NIHSS	The proportion of patients with >=90% improvement from baseline NIHSS was significantly higher in the intervention group (OR 3.94, 95% CI: 1.72-9.00).
Mohan et al, 2015	70	8 weeks	Intravenous amino acids plus enteral nutrition	Enteral nutrition	Mortality	Thirty-day mortality was 40% in the intervention group and 67% in the control group.
Momosaki et al, 2019	97	8 weeks	Vitamin D supplement (2,000 IU)	Placebo	BI; grip strength; muscle mass	No significant difference was observed in grip strength change (MD 0.50, 95% CI: 0.85 to 1.85) or BI at the end of intervention (MD 0.04, 95% CI: 0.43 to -0.36).
Ogawa, 2021	20	>=14 days	Fatty acid (eicosapentaenoic acid) with enteral nutrition	Usual care	Pressure ulcers	There was no significant difference in pressure ulcer risk (OR 0.10, 95% CI: 0.00-2.28).
Otsuki et al, 2020	124	During hospitalization (mean 28-30 days)	Individualized nutrition therapy considering stress and physical activity level	Usual care based only on body weight	FIM; body weight; muscle mass	No significant difference was observed in FIM score at the end of intervention (MD 8.60, 95% CI: 0.74-17.94). There was no difference in weight loss. Thigh circumference increased significantly in the intervention group (paretic side MD 1.40 [95% CI: 0.25-2.55]; non-paretic side MD 1.60 [95% CI: 0.38-2.82]).

Author, year	N	Intervention period	Intervention	Control	Outcomes	Findings
Ouyang et al, 2003	62	NA	Early enteral nutrition within 48 hours after onset	Usual care with feeding started on days 3-4 after onset	Muscle mass; complications	Mid-arm muscle circumference at the end of intervention was significantly higher in the intervention group (MD 2.46 [95% CI: 1.40-3.52]). Vomiting incidence was significantly lower (OR 0.19 [95% CI: 0.05-0.79]).
Saito et al, 2017	41	60 days	Fatty acids (EPA 184 mg, DHA 1,520 mg) intravenously for 5 days followed by oral administration	Usual care	Degree of disability	No significant difference was observed in the Glasgow Outcome Scale Extended (GOS-E) score (OR 0.61 [95% CI: 0.17-2.16]).
Tajiri, 2008	41	10 days	Intervention 1: high-protein enteral formula with nonessential amino acids; Intervention 2: high-essential-amino-acid enteral formula	Comparison between the two intervention groups	Death at discharge	No difference in death was observed (2 cases in each group).
Ullegaddi et al, 2005a	48	14 days	Oral vitamin C 500 mg plus vitamin D 800 IU	Usual care	Complications	No significant difference was observed in infections (pneumonia, urinary tract infection, sepsis).
Ullegaddi et al, 2005b	72	14 days	Intervention 1: vitamins E + C; Intervention 2: vitamin B; Intervention 3: vitamins E + C + B	Usual care	Complications	No significant difference was observed in infections (pneumonia, urinary tract infection, sepsis).
Zhang et al, 2004	107	NA	Intervention 1: early enteral nutrition; Intervention 2: early parenteral nutrition	Usual care (enteral nutrition started on day 5 after admission)	Death; ADL	No significant difference was observed in ADL independence (OR 1.79, 95% CI: 0.70 to 4.60) or follow-up mortality (OR 0.96, 95% CI: 0.33 to 2.82).
Zhang et al, 2014	89	14 days	High-protein enteral nutrition (1.6 kg/kg body weight)	Protein 0.9 g or 1.2 g	Muscle mass	Calf circumference at the end of intervention was significantly higher in the high-protein group (36.04 +/- 5.11 vs 30.17 +/- 6.60 / 30.48 +/- 5.54).
Zhao, 2020	78	NA	Early enteral nutrition within 3 days after onset	Usual care (enteral nutrition started on days 4-7)	BI; complications; muscle mass	No significant difference was observed in BI score at the end of intervention (MD 0.41, 95% CI: 2.05 to 1.23). Mid-arm muscle circumference was significantly higher in the intervention group (MD 0.97, 95% CI: 0.25-1.69). Diarrhea incidence did not differ significantly.
Zheng et al, 2006	49	21 days	Energy plus multiple nutrients (enteral nutrition)	Usual care (family-managed enteral nutrition)	Muscle mass; complications	No significant difference was observed in mid-arm muscle circumference at the end of intervention. No significant differences were observed in pneumonia, urinary tract infection, or pressure ulcer.
Zheng et al, 2015	146	21 days	Early enteral nutrition	Usual care (family-managed ordinary diet)	Death; BI; complications; muscle mass	Mortality was significantly lower in the intervention group (OR 0.32, 95% CI: 0.11-0.95). No significant difference was observed in BI score at the end of intervention (MD 2.30 [-0.64, 5.24]). Mid-arm muscle circumference was significantly higher in the intervention group (MD 1.21, 95% CI: 0.52-1.90). No significant difference was observed in pneumonia, urinary tract infection, or pressure ulcer risk.

Author, year	N	Intervention period	Intervention	Control	Outcomes	Findings
Zhou et al, 2006	51	14 days	High-protein enteral nutrition (PFC ratio 20:45:35)	Usual care (enteral nutrition, PFC ratio 16:45:35)	Mortality	Mortality was lower in the intervention group.

Supplementary Table 2-1. Structured abstracts (CQ2)

Author, year	N	Intervention period	Intervention / control	Outcomes	Findings
Aquilani et al, 2008a	48	21 days	Intervention: energy-protein oral supplementation (250 kcal, 20 g protein). Control: usual care (voluntary supplementation).	Body weight	Weight gain was observed in the intervention group (intervention +0.36 +/- 0.63; control -0.5 +/- 1.5).
Aquilani et al, 2008b	42	21 days	Intervention: energy-protein oral supplementation (250 kcal, 20 g protein). Control: usual care (voluntary supplementation).	Body weight	Body weight did not change during the intervention in either group (intervention 66.2 +/- 8.7 to 65.9 +/- 8.8; control 64 +/- 9.6 to 64.4 +/- 9).
Aquilani et al, 2009	26	30 days	Patients with zinc deficiency. Intervention: zinc supplementation (10 mg as Zn2+). Control: placebo.	Body weight	A statistically significant between-group difference was observed over 30 days (P=0.002).
Aquilani et al, 2014	38	Until discharge (mean 42 +/- 4 days)	Intervention: essential amino acids (8 g). Control: placebo.	FIM; body weight	No significant difference in FIM (P=0.5; EAA 27.6 +/- 21.7 to 47.4 +/- 23.9; placebo 31.1 +/- 16 to 60 +/- 36.8). No significant difference in body weight (P=0.6; EAA 62.2 +/- 12.7 kg to 59.7 +/- 12 kg; placebo 57.6 +/- 7.1 kg to 55.9 +/- 7.5 kg).
Aquilani et al, 2015	42	35 days	Intervention: essential amino acids 8 g/day (4 g in the morning and 4 g in the afternoon diluted in water). Control: placebo.	FIM; body weight	FIM scores improved significantly in both groups (mean +69%, P<0.001), but the pattern of improvement did not differ significantly between groups. Body weight decreased significantly in both groups (mean -1.5 kg, P<0.02), with a similar temporal pattern between the EAA and placebo groups.
Rabadi et al, 2008	116	14.10 +/- 11.23 days	Intervention: energy-protein supplementation (hospital diet plus 240 kcal and 11 g protein). Control: usual care (hospital diet plus supplement containing 127 kcal and 5 g protein).	FIM; gait speed	Total FIM score, motor FIM score, and gait speed improved significantly in the intervention group. Cognitive FIM did not differ significantly.
Dang et al, 2018	22	4 weeks	Intervention: early enteral nutrition. Control: usual diet.	ADL	There was no significant difference in ADL between groups.
Garbagnati et al, 2009	72	12 months	Intervention 1: vitamin E 290 mg + vitamin C 240 mg; Intervention 2: fatty acids 500 mg; Intervention 3: vitamins plus fatty acids. Control: placebo.	Barthel Index	No significant intervention effect was observed (vitamin group 77.78 +/- 19.06; fatty acid group 83.85 +/- 22.66; vitamin + fatty acid group 80.50 +/- 19.06; placebo 73.64 +/- 24.50).
Honaga et al, 2022	50	16 weeks	Intervention: protein plus vitamins (100 kcal, carbohydrate 15 g, fat 0 g, protein 10 g, and vitamin D 20 micrograms per pack, two packs/day). Control: placebo.	FIM motor score; gait speed; muscle mass	No statistically significant between-group differences were found in SMI or thigh muscle cross-sectional area on either the paretic or non-paretic side. Grip strength, knee extension strength, and FIM motor score improved significantly in both groups during the intervention, but between-group differences were not significant. No significant between-group differences were observed in the 10-m walk test or Timed Up and Go test.
Pan et al, 2017	291	6 months	Intervention 1: potassium-enriched salt; Intervention 2: potassium- and magnesium-enriched salt; Control: ordinary salt; each 1 kg/month.	Mortality	Mortality did not differ significantly between groups.

Author, year	N	Intervention period	Intervention / control	Outcomes	Findings
Toole et al, 2004	56	2 years	Intervention: high-dose multivitamin (pyridoxine 25 mg, cobalamin 0.4 mg, folic acid 2.5 mg). Control: low-dose vitamin (pyridoxine 200 micrograms, cobalamin 6 micrograms, folic acid 20 micrograms).	Mortality	Mortality did not differ significantly (high-dose 99 persons, low-dose 117 persons).
Torrisi et al, 2021	40	12 weeks	Intervention: vitamin D 2,000 IU. Control: placebo.	FIM	The intervention group showed a significant FIM improvement from rehabilitation start (T0) to completion (T1) ($P<0.001$; T0 58.73 +/- 32.05; T1 95.53 +/- 28.89). The control group also improved significantly ($P<0.001$; T0 63.45 +/- 25.72; T1 80.09 +/- 29.12).
Yoshimura et al, 2014	49	8 weeks	Intervention: amino acids (3 g; essential amino acids enriched with 40% leucine). Control: usual diet.	FIM; grip strength; skeletal muscle index	FIM scores increased significantly in both groups, with significantly greater improvement in the intervention group ($P<0.045$). Grip strength increased significantly in both groups, with significantly greater improvement in the intervention group ($P<0.01$). Skeletal muscle index increased significantly only in the intervention group, and the improvement was significantly greater than in the control group.

Note: Rehabilitation is abbreviated as rehab where used.

Supplementary Table 3-1. Structured abstracts (CQ3)

Author, year	Study design	N	Participants	Intervention	Control	Main outcomes	Findings
Shi H et al, 2020	Controlled trial	48	Patients aged >=60 years with femoral trochanteric fracture	Nutritional supplements three times daily until postoperative day 3	Usual care	Length of stay, albumin, IL-6, CRP, PCT, bed days, four EQ-5D QoL domains	Early enteral nutrition reduced days in bed but did not reduce length of stay.
Bastow MD et al, 1983	Controlled trial	122	Malnourished women with femoral fracture	Usual diet plus nasogastric feeding within 5 days after surgery (1,000 kcal, protein 28 g for 8 hours overnight)	Usual care	Mortality, aspiration, length of stay, days to weight-bearing and independent mobility	Nutritional care shortened hospital stay.
Cameron ID et al, 2011	Controlled trial	44	Malnourished older female patients after fracture	One nutritional supplement per day	Milk 150 mL	Nutritional status, ADL, length of stay, readmission	No significant differences were observed in nutritional or functional indices; efficacy could not be concluded.
Duncan DG et al, 2006	Controlled trial	318	Older hospitalized women with acute non-pathological hip fracture	Additional nutrition support by dietetic assistants	Standard nursing only	Postoperative mortality on acute ward, 4-month mortality, length of stay, energy intake, nutritional status	Dietetic assistant support improved energy intake and significantly reduced acute-stage and 4-month mortality.
Ekinci O et al, 2016	Controlled trial	75	Older hospitalized women with proximal femoral fracture	Enteral product containing CaHMB, vitamin D, and protein	Standard postoperative nutrition only	Wound healing time, mobilization status, muscle strength, anthropometric and laboratory indices	The intervention promoted wound healing and significantly improved early mobilization rate and muscle strength at postoperative day 30.
Flodin L et al, 2015	Controlled trial	79	Patients with proximal femoral fracture	High-protein nutritional supplementation	Calcium plus vitamin D only	Body composition, grip strength, health-related QoL	No significant additive effect on maintenance of FFMI was shown, but grip strength and QoL tended to improve.
Milte R et al, 2016	Controlled trial	175	Frail older patients after proximal femoral fracture	Comprehensive program by physiotherapist and dietitian	Social visits only	Health and long-term care cost, QALY, incremental cost-effectiveness ratio	The comprehensive intervention showed small cost differences and a slight QALY increase; cost-effectiveness was estimated to be within an acceptable range, with high uncertainty.
Myint MW et al, 2013	Blinded randomized controlled trial	126	Older patients with proximal femoral fracture	Additional oral nutritional supplements	Standard hospital diet only	Serum albumin, BMI, FIM, EMS, complications, length of stay, death, acute readmission	ONS limited BMI decline, shortened length of stay, and reduced infection episodes, but did not improve FIM or EMS.
Niitsu M et al, 2016	Controlled trial	38	Older postoperative patients with proximal femoral fracture	Whey protein 32.2 g	Usual care	Knee extension strength (Biodex), Barthel Index items	The whey protein group had significantly greater improvement in operated-side knee extension strength and Barthel Index transfer, walking, and toileting items.

Author, year	Study design	N	Participants	Intervention	Control	Main outcomes	Findings
Tidermark J et al, 2004	Randomized controlled trial	60	Thin older women after femoral neck fracture	High-protein oral supplement (20 g protein/day for 6 months); high-protein supplement plus intramuscular nandrolone every 3 weeks	Usual treatment	Lean body mass, ADL (Katz index), EQ-5D, body weight, fracture healing	Protein plus nandrolone tended to suppress loss of LBM and decline in ADL and QoL.
Renerts K et al, 2019	Randomized controlled trial	173	Older patients after proximal femoral fracture	High-dose vitamin D (2,000 IU/day)	Vitamin D 800 IU/day	Health-related QoL (EQ-5D-3L)	HE and/or high-dose vitamin D did not significantly improve HRQL decline over the full period, but may have suppressed further HRQL decline in the later recovery period of 6-12 months.
Wang L et al, 2024	Randomized controlled trial	106	Older postoperative patients with vertebral or hip fracture (VOHF)	Nutritional nursing plus Baduanjin exercise	Usual care	Fracture healing, nutritional indices, physical function, pain, QoL, adverse events	Fracture healing, nutritional indices, functional recovery, and QoL improved significantly more in the intervention group, and adverse events were fewer.
Scaturro D et al, 2022	Controlled trial	75	Patients with proximal femoral fracture and hypomagnesemia	Magnesium plus vitamin D supplementation	Standard hospital diet only	Serum magnesium, pain (NRS), balance (Tinetti scale), QoL (SarQoL)	The magnesium supplementation group had significant improvements in serum Mg, pain, balance, and QoL.
de Sire A et al, 2020	Randomized controlled trial	20	Older patients after proximal femoral fracture	Amino acid supplementation	Usual care	Serum myostatin and sarcopenia-related indices	Myostatin decreased after rehab in both groups, with a more pronounced decrease in sarcopenic patients receiving amino acid supplementation.
Nie N et al, 2023	Randomized controlled trial	100	Patients with femoral fracture and sarcopenia	Nutrition intervention plus strengthening exercise	Usual care	Sarcopenia-related indices, ADL, fracture healing time, length of stay, pain, nursing satisfaction	Combined nutrition intervention and strengthening exercise improved sarcopenia indices and ADL and significantly improved fracture healing time, length of stay, and pain.
Han CY et al, 2021	Randomized controlled trial	175	Patients aged >=70 years after proximal femoral fracture	Individualized exercise therapy plus nutrition intervention for 6 months	Usual care	Gait speed, physical function, strength, balance, body composition, energy/protein intake	Combined exercise and nutrition did not significantly improve gait speed or physical function, but increased energy and protein intake.
Baek Y et al, 2023	Cluster randomized trial	7,224	Older residents of long-term care facilities	Nutrition intervention with dairy products (milk, yogurt, cheese; calcium approximately 1,142 mg/day, protein approximately 69 g/day)	Usual diet (calcium approximately 700 mg/day, protein approximately 58 g/day)	Fracture occurrence, medical costs, cost-effectiveness (ICER)	A high-protein, high-calcium diet reduced fracture occurrence and contributed to lower medical costs from a cost-effectiveness perspective.

**Author, year**	**Study design**	**N**	**Participants**	**Intervention**	**Control**	**Main outcomes**	**Findings**
Han Z et al, 2022	Randomized controlled trial	200	Patients aged >=65 years with femoral neck fracture and sarcopenia	Resistance training plus HMB supplementation	RT alone, HMB alone, or no intervention	Body composition, grip strength, Barthel Index, Harris Hip Score, pain	Resistance training, alone or with HMB, significantly improved body composition and grip strength, but no between-group differences were observed in ADL or hip function.
Wyers CE et al, 2018	Randomized controlled trial	152	Older patients after proximal femoral fracture	Intensive nutrition intervention for 3 months	Usual care	Total length of stay including hospital and recovery period, nutritional status, physical function, cognition, QoL, postoperative complications, refracture, death	Nutrition intervention improved nutritional intake and status at 3 months, but did not affect length of stay or clinical outcomes.

Note: Rehabilitation is abbreviated as rehab where used.

Supplementary Table 4-1. Structured abstracts (CQ4)

Author, year	Study design	N	Intervention period	Participants	Intervention	Control	Main outcomes	Findings
Baldi et al, 2010	Randomized controlled trial	Intervention 14; control 14; total 28	12 weeks	Severe COPD by ATS criteria with dynamic weight loss >=5% in the past 6 months; age 73.1 +/- 6.0 vs 70.1 +/- 5.8 years	Essential and nonessential amino acid mixture 4 g twice daily in 200 mL liquid supplement	No supplementation	Body weight; blood glucose	At 12 weeks, body weight increased by 3.8 +/- 2.6 kg in the intervention group and -0.1 +/- 1.1 kg in controls. Fat-free mass also increased, indicating that EAA supplementation contributed to improvements in body weight and fat-free mass.
de Bisschop et al, 2021	Randomized controlled trial	Intervention 30; control 30; total 60	4 weeks	Stable COPD GOLD stage 2 or 3; FEV1 30-80%, FEV1/FVC <70%; age 40-80 years; agreed to smoking cessation and pulmonary rehab program	BCAA supplement containing 18.44 g protein, 1.59 g carbohydrate, 0.51 g fat, 2.67 g fiber, and 0.3 g salt; each dose contained 4.3 g BCAA	Control supplement with 0.7 g protein, 2.05 g carbohydrate, 0.38 g fat, and 19 g fiber	Peak oxygen uptake, 6MWD, BMI, quadriceps strength, VQ11	A 4-week pulmonary rehab program with BCAA supplementation was not more beneficial than pulmonary rehab alone.
Deacon et al, 2008	Randomized controlled trial	80	8 weeks	Patients with COPD referred to pulmonary rehabilitation; intervention age 67.6 +/- 7.4, control 68.3 +/- 8.2	Creatine loading 22 g/day for 5 days, then maintenance 3.76 g/day during PR plus PR	Placebo plus PR	Weight, ISWT, isometric quadriceps strength	The PRNS group had increases in body weight, BMI, FFMI, and mid-thigh CSA. Exercise tolerance and QoL also improved in the PR group; the main additive effect was on body composition indices.
Fuld et al, 2005	Randomized controlled trial	38	10 weeks	Stable moderate-to-severe COPD; no medication change, no oral corticosteroids, no exacerbation in past 4 weeks	Creatine plus glucose polymer (creatine monohydrate 5.7 g, equivalent to creatine 5 g and glucose 35 g per dose)	Glucose polymer only (40.7 g per dose)	FEV1, MIP, weight, grip peak force, knee extension peak torque, ISWT distance, SGRQ	Creatine supplementation increased BMI, lower-limb muscle strength, and endurance.
Gouzi et al, 2019	Randomized controlled trial	Intervention 32; control 32; total 64	28 days	COPD with post-bronchodilator FEV1/FVC <70%; age 62.4 +/- 6.5 vs 61.1 +/- 8.7 years	Antioxidant supplement containing vitamin E 30 mg/day, vitamin C 180 mg/day, zinc gluconate 15 mg/day, selenium 50 micrograms/day	Placebo	Quadriceps strength, 6MWD, peak oxygen uptake, BMI	Antioxidant supplementation with pulmonary rehab did not improve quadriceps endurance (primary outcome), but improved quadriceps strength and serum total protein and tended to increase type I fiber proportion.
Gurgun et al, 2013	Randomized controlled trial	46	8 weeks	COPD with at least one of BMI <21 kg/m2, low fat-free mass, or weight loss before admission	Pulmonary rehab plus nutrition support (PRNS)	Pulmonary rehab (PR)	6MWT, ISWT, SGRQ domains, BMI, body weight	PRNS increased BMI compared with PR and control for the outcomes adopted.

Author, year	Study design	N	Intervention period	Participants	Intervention	Control	Main outcomes	Findings
Hornikx et al, 2012	Randomized controlled trial	Intervention 25; control 25; total 50	3 months	Age >=50 years, current/former smokers, COPD by GOLD criteria, clinically indicated for pulmonary rehab	Monthly vitamin D 100,000 IU	Placebo (4 mL arachidis oleum)	Quadriceps strength, maximal inspiratory/expiratory pressure, 6MWD, peak oxygen uptake	High-dose vitamin D supplementation during rehab may add a modest training effect.
Schols et al, 1995	Randomized controlled trial	Control 38; intervention 1: 38; intervention 2: 33; total 109	Baseline, day 29, day 57	Patients with moderate-to-severe COPD admitted consecutively for intensive inpatient pulmonary rehab	Nandrolone decanoate placebo plus nutritional supplementation; or nandrolone decanoate plus nutritional supplementation	Nandrolone decanoate placebo	Body weight; respiratory muscle strength (PImax)	Combined nutritional supplementation and short-course anabolic steroid may promote increases in FFM and respiratory muscle function without adverse effects.
Steiner et al, 2003	Randomized controlled trial	Intervention 42; control 43; total 85	7 weeks	Patients meeting clinical and spirometric COPD criteria, stable at enrollment, optimized medical treatment before referral to rehab	125 mL supplement three times daily; 570 kcal/day; carbohydrate 60%, fat 20%, protein 20%	Non-nutritive placebo	ISWT, grip strength, quadriceps strength, body weight, BMI	When prescribed universally, carbohydrate supplementation did not facilitate rehab in COPD patients.
Sugawara et al, 2012	Randomized controlled trial	Intervention 18; control 18; total 36	3 months	Stable older COPD patients with IBW <110% and FEV1 <80% undergoing low-intensity exercise therapy according to pulmonary rehab program	MEINa 200 kcal/200 mL (protein 20%, fat 25%, carbohydrate 53.2%, fiber 1.8%, Fischer ratio 3.7%) twice daily for 12 weeks plus diet provision with nutrition counseling	Usual diet and dietary counseling only	Body weight, PImax, 6MWD, QoL (CRQ)	Adding anti-inflammatory nutritional supplementation with whey peptide to low-intensity exercise therapy may suppress systemic inflammation and improve exercise tolerance and HRQoL in stable older COPD patients.

Note: Rehabilitation is abbreviated as rehab where used.

Supplementary Table 5-1. Structured abstracts (CQ5)

Author, year	Study design	N	Participants	Intervention	Control	Main outcomes	Findings
Uster A et al, 2018	Randomized controlled trial	58	Patients with metastatic or locally advanced gastrointestinal or lung tumors; ECOG PS <=2; expected survival >=6 months by physician judgment	Nutrition therapy (>=3 nutrition counseling sessions and supplements) plus rehab (supervised strength and balance training by physiotherapist, twice weekly, 60 min/session)	Standard care	Health status/QoL (EORTC QLQ-C30 v3.0), grip strength, 1RM leg strength, bioelectrical impedance, weight, unplanned hospital days, estimated 3-month survival	The nutrition and exercise program did not show significant improvement versus control for the outcomes. However, nausea/vomiting symptom scores improved in the intervention group (P=0.023), and protein intake increased significantly (intervention +6 +/- 4 g/day; control -2 +/- 5 g/day; P=0.01).
Houben LHP et al, 2023	Double-blind randomized controlled trial	96	Patients with prostate cancer starting or continuing androgen deprivation therapy for at least 6 months	Resistance exercise plus nutrition therapy group and resistance exercise alone group	Standard care	Lean mass, fat mass, skeletal muscle mass, body weight, BMI, leg extension strength, leg press strength	In advanced prostate cancer patients receiving androgen deprivation therapy, resistance exercise training was safe, feasible, and well tolerated and countered adverse effects on body composition, muscle mass, strength, and aerobic capacity. The rehab groups showed significant improvements in grip strength, lower-limb strength, and 1-RM (P<0.05), with significant increase in quadriceps cross-sectional area (P<0.001) and suppression of fat gain.
Bourke L et al, 2011	Randomized controlled trial	50	Patients with advanced prostate cancer receiving androgen deprivation therapy for at least the previous 6 months	Supervised aerobic and resistance exercise plus nutrition therapy (dietary counseling)	Standard care	BMI, quadriceps strength, QoL (FACT-F fatigue, FACT-Physical, FACT-General), exercise tolerance	Fatigue improved significantly in the intervention group compared with control (P=0.002), but prostate-specific QoL indices did not change significantly.
Focht BC et al, 2018	Randomized controlled trial	32	Patients diagnosed with prostate cancer, currently receiving androgen deprivation therapy with at least 3 months of continued treatment planned, and <60 min/week of exercise in the past 6 months	Behavioral intervention program combining rehab and nutrition therapy	Standard care	Fat mass, body fat percentage, body weight, lean body mass, strength (1-RM test)	The intervention group had significant improvements in grip strength, lower-limb strength, and 1-RM (P<0.05), as well as body fat percentage (P<0.05) and fat mass (P<0.03). Individualized exercise plus nutrition yielded clinically meaningful improvements in mobility, body composition, and strength, suggesting feasibility, safety, and preliminary efficacy.

Author, year	Study design	N	Participants	Intervention	Control	Main outcomes	Findings
Lonbro S et al, 2013	Randomized controlled trial	30	Patients with histologically diagnosed squamous cell carcinoma of the larynx, pharynx, oral cavity, or lymph-node metastasis of unknown primary after curative radiotherapy +/- chemotherapy, complete remission, no metastasis	Rehab intervention plus creatine and protein supplementation	Rehab plus placebo supplementation	Lean body mass, fat mass, isometric and isokinetic knee extensor/flexor strength	Both intervention and control groups showed significant strength improvement, suggesting efficacy of rehab; the additional effect of nutrition therapy was unclear.

Note: Rehabilitation is abbreviated as rehab where used.

Supplementary Table 6-1. Structured abstracts (BQ1)

Author, year	Study design	N	Intervention period	Participants	Intervention	Control	Main outcomes	Findings
Eriksson H et al, 2023	RCT	50	8 weeks	Community-dwelling patients after radiotherapy/chemotherapy for head and neck cancer	Head-lift training (Shaker exercise): sustained head lift 60 s x3 and consecutive head lifts x30, three times/day	Usual dysphagia rehab	Voice function (GRBAS, vocal fry, VHI)	No significant improvement in GRBAS or VHI.
Tuomi L et al, 2022	RCT	61	8 weeks	Community-dwelling patients after radiotherapy/chemotherapy for head and neck cancer	Shaker exercise: sustained head lift 60 s x3 plus 30 consecutive isotonic lifts, three times/day	Usual dysphagia rehab	Swallowing function (PAS, SPS); HRQoL (EORTC QLQ-H&N35)	Only health-related QoL improved.
Park JS et al, 2017	RCT	37	4 weeks	Inpatients with post-stroke dysphagia	Shaker exercise: sustained head lift 60 s x3 plus consecutive head lifts x30, 5 times/week	Usual dysphagia rehab	Swallowing function (PAS); hyoid movement	Improved upward hyoid movement and reduced liquid aspiration.
Mepani R et al, 2009	RCT	19	6 weeks	Community-dwelling patients with dysphagia after stroke or chemoradiotherapy for head and neck cancer	Shaker exercise, three times/day	Usual dysphagia rehab	Swallowing function	Improved thyrohyoid muscle strength.
Logemann JA et al, 2009	RCT	19	6 weeks	Community-dwelling patients with dysphagia after stroke or chemoradiotherapy for head and neck cancer	Shaker exercise, four times/day	Usual dysphagia rehab	Swallowing function	Reduced liquid aspiration and increased upper esophageal sphincter opening.
Shaker R et al, 2002	RCT	27	6 weeks	Community-dwelling patients with dysphagia	Shaker exercise, three times/day	Sham exercise	Swallowing function (FOAMS)	Increased upper esophageal sphincter opening and laryngeal elevation.
Dotevall H et al, 2022	RCT	61	8 weeks	Community-dwelling patients after chemoradiotherapy for head and neck cancer	Shaker exercise with sustained and isotonic lifts, three times/day	Written, video, and oral instruction	PAS, EAT-10, SPS	Only EAT-10 improved significantly.
Jensen D et al, 2022	RCT	90	6 weeks	Community-dwelling patients with dysphagia	Chin tuck against resistance (CTAR), three times/day	Usual dysphagia rehab including environmental and diet modification	V-VST, MEOF-II, Dysphagia Handicap Index, Barthel 20, EQ-5D, nutrition intake, BMI, grip strength, FOIS	Swallowing function and health-related QoL improved.

Author, year	Study design	N	Intervention period	Participants	Intervention	Control	Main outcomes	Findings
Park JS et al, 2019	RCT	46	4 weeks	Inpatients with post-stroke dysphagia	Game-based CTAR at 70% 1-RM, isometric 60 s x3 and isotonic x30, 5 times/week plus usual rehab	Shaker exercise plus usual rehab	VDS, PAS, FOIS, adherence-related evaluation	No significant difference in improvement in swallowing function; motivation, interest, and enjoyment were higher, and physical effort and muscle fatigue were lower.
Koyama Y et al, 2017	RCT	16	6 weeks	Inpatients with post-stroke dysphagia	Modified jaw-opening exercise at 80% MVC, 6 s x5, 4 sets/day, 5 times/week	Sham isometric jaw-closing exercise at 20% MVC	Swallowing function	Anterior hyoid movement increased.
Choi JB et al, 2020	RCT	21	6 weeks	Inpatients with post-stroke dysphagia (1-5 months after onset)	CTAR: isometric 60 s x3 and isotonic x30, 5 times/week	Head-lift training	Swallowing function, suprahyoid muscle mass, hyoid movement, Borg RPE	Both groups increased suprahyoid muscle mass and hyoid excursion; perceived exertion was significantly lower with CTAR.
Park JS et al, 2020	Double-blind RCT	40	5 weeks	Community-dwelling patients with post-stroke dysphagia within 6 months	Resistive jaw-opening exercise (RJOE): isometric 30 s x3 and isotonic 2-3 s push-down x10, three sets	Training with sham device	PAS, FOIS	PAS improved during liquid intake.
Kim HH et al, 2019	RCT	30	6 weeks	Inpatients with post-stroke dysphagia	CTAR: isometric 10 s x3 and consecutive isotonic x30, 5 days/week	Usual dysphagia rehab	PAS, FOIS, transition to oral intake	PAS and FOIS improved significantly; transition to oral intake was 25% in the intervention group and 15% in control.
Park JS et al, 2018	Single-blind RCT	25	4 weeks	Patients with post-stroke dysphagia within 12 months	CTAR: isometric 60 s x3 and consecutive isotonic x30	Usual dysphagia rehab	FDS, PAS	FDS and PAS improved significantly.
Acar G et al, 2022	Single-blind RCT	40	6 weeks	Children aged 18-54 months with cerebral palsy and eating/swallowing disorders and their families	Oral motor intervention strategies plus nutrition-related caregiver training plus NDT-Bobath	Oral motor intervention strategies plus nutrition-related caregiver training	EDACS, SOMA, PedsQL	Head and neck stabilization and feeding function improved.
Wang T et al, 2022	Single-blind RCT	37	4 weeks	Inpatients with post-stroke dysphagia	Tongue-pressure resistance training (TPRT), 5 days/week, 20 min/day	Usual dysphagia rehab	FCM, OMFS, MTP, MSS, YPRSRS, PAS	Swallowing and oral motor function improved; tongue strength increased.
Moon JH et al, 2018	Single-blind RCT	19	8 weeks	Inpatients with post-stroke dysphagia	Tongue-pressure strength and accuracy training, 30 min/day, 5 times/week	Usual dysphagia rehab	MIP, MASA, SWAL-QOL	Swallowing function improved; anterior and posterior tongue strength increased.

Author, year	Study design	N	Intervention period	Participants	Intervention	Control	Main outcomes	Findings
Wakabayashi H et al, 2018	Cluster RCT	104	12 weeks	Community-dwelling adults aged >=65 years with dysphagia	Resistance training of swallowing muscles (tongue resistance and manual-resisted head/neck flexion), 10 s x10, two sets/day, 3 times/week	Pamphlet on dysphagia rehab	EAT-10, tongue pressure, MNA-SF, Barthel Index	No improvement in swallowing function was observed in either group.
Kim HD et al, 2017	RCT	41	4 weeks	Inpatients with post-stroke dysphagia	Tongue-to-palate resistance training: 30 repetitions each anterior and posterior tongue, 5 times/week plus usual rehab	Usual dysphagia rehab	ATS, PTS, VDS, PAS	Swallowing function improved and tongue strength increased.
Steele CM et al, 2016	RCT	14	8-12 weeks; 24 sessions	Inpatients with post-stroke dysphagia	Tongue-pressure profile training	Tongue-pressure strength and accuracy training	Tongue strength, VFSS, PAS, NRRS	Both groups improved tongue strength during swallowing and reduced pharyngeal residue after liquid intake.
Claus I et al, 2021	Double-blind RCT	50	4 weeks	Outpatients with Parkinson disease	Expiratory muscle strength training (EMST), 5 days/week, 5 x 5 sets	Training with sham device	FEES, SWAL-QOL, SDQ	Swallowing function improved and pharyngeal residue decreased.
Park JS et al, 2016	Single-blind RCT	33	4 weeks	Inpatients with post-stroke dysphagia	EMST at approximately 70% maximum expiratory pressure, 5 repetitions/set, 5 sets/day, 5 times/week	Training with sham device	PAS, FOIS, surface EMG	Swallowing during liquid intake improved; suprahyoid muscle activity increased.
Troche MS et al, 2010	RCT	68	4 weeks	Community-dwelling patients with Parkinson disease	EMST at 75% mean MEP, 5 repetitions/set, 5 sets/day, 5 days/week	Sham EMST device	Penetration-aspiration score, SWAL-QOL	PAS scores improved; hyoid movement increased.
Riboldazzi G et al, 2020	RCT	25	48 weeks	Inpatients with Parkinson disease	Expiratory flow acceleration with Free-Aspire device, 20 min/session, three times/day, 7 days/week	Usual dysphagia rehab	PDQ-39, cough VAS, VNS, EuroQoL-VAS	Respiratory function and health-related QoL improved.
Eom MJ et al, 2017	RCT	30	4 weeks	Inpatients with post-stroke dysphagia	EMST at 70% maximum expiratory pressure, 5 repetitions/set, 5 sets/day, 5 times/week	Sham EMST device	VDS, PAS	Swallowing function improved.
Zhang J et al, 2021	RCT	214	4 weeks	Older patients with vascular dementia and dysphagia	Mendelsohn maneuver once daily for 10 min plus swallowing training in reclining or lateral position	Usual dysphagia rehab	Water-swallowing test, VFSE, ADL scale	Swallowing function improved.
Park HS et al, 2019	Double-blind RCT	30	4 weeks	Inpatients with post-stroke dysphagia	Effortful swallowing training, 10 repetitions/set, three times/day, five times	Usual dysphagia rehab	Tongue strength, VFSS, VDS	Oral-phase swallowing function improved; anterior and posterior tongue strength increased.

Author, year	Study design	N	Intervention period	Participants	Intervention	Control	Main outcomes	Findings
Hagglund P et al, 2019	Cluster RCT	36	5 weeks	Older patients with dysphagia	Oral neuromuscular training device (IQoro), 5-10 s/repetition, three times/day	Usual dysphagia rehab	Water swallow test, swallowing-related QoL questionnaire	Swallowing speed and swallowing function during liquid intake improved.
Krajczy E et al, 2019	RCT	60	15 days	Patients with dysphagia after cerebral infarction	Composite training, 60 min/day for 15 days	Usual dysphagia rehab	Voice quality, number of swallows, swallowing time	Swallowing reflex improved.
Jakobsen D et al, 2019	Pilot RCT	10	3 weeks	Patients with dysphagia due to severe brain injury	Facial Oral Tract Therapy (F.O.T.T.), 30 min/day, 7 days/week	Intraoral and facial stimulation	EFA Scale, FOIS, PAS, EMBI	PAS and FOIS improved in both groups, but no significant difference was observed.
Ploumis A et al, 2018	RCT	70	12 weeks	Inpatients with post-stroke dysphagia	Cervical isometric strengthening exercises plus PT, OT, and ST	Sitting balance training plus PT, OT, and ST including swallowing strengthening and compensatory techniques	Water swallow test, cervical auscultation, VFSS	Cervical alignment and swallowing function improved.
Sayaca C et al, 2020	RCT	50	6 weeks	Community-dwelling adults aged >=65 years with dysphagia	Proprioceptive neuromuscular facilitation (PNF) exercises, 30 repetitions/set, 1 set/day, 3 times/week	Head-lift/Shaker exercise	EAT-10, water swallowing test, sEMG, swallow volume/capacity/speed	Swallowing function improved in both groups; suprahyoid muscle strength increased significantly in the intervention group.
Hwang NK et al, 2019	Double-blind RCT	25	4 weeks	Outpatients with post-stroke dysphagia	Tongue stretching exercises plus TDT, 20 repetitions/day, 5 times/week	Usual dysphagia rehab	VFSS, VDS, tongue motility	Tongue movement, bolus formation, tongue-palate contact, and bolus transit time improved significantly.
Gao J et al, 2017	RCT	90	10.5 weeks	Inpatients with post-stroke dysphagia	CTAR 30 repetitions/set, 3 sets/day, 7 days/week for 42 days, and Shaker exercise 30 repetitions/set, 3 sets/day, 7 days/week	Usual dysphagia rehab	Swallowing function	Swallowing function improved significantly with CTAR and Shaker exercise.

Note: Rehabilitation is abbreviated as rehab where used.

Supplementary Table 7-1. Structured abstracts (BQ2)

Author, year	Study design	N	Intervention period	Participants	Intervention	Control	Main outcomes	Findings
Wu XS et al, 2022	RCT	22	6 weeks	Facility residents aged >=65 years consuming dysphagia diets	Energy- and protein-rich hydrolyzed meat puree diet	Usual puree diet	Energy, protein, and fat intake, nutritional status, weight gain, body composition, calf and upper arm circumference, grip strength	Energy, protein, and fat intake increased; MNA-SF score increased; body weight increased.
Germain I et al, 2006	RCT	17	12 weeks	Facility residents aged >=65 years with dysphagia, BMI <24 or >=7.5% weight loss in 3 months	Texture-modified minced or puree diet, thickened fluids as needed, supplements, new menu in 3-week cycles, individualized preferences	Usual menu	Macro- and micronutrient intake, body weight, BMI	Body weight increased. Intake increased for energy, protein, fat, saturated and monounsaturated fatty acids, potassium, magnesium, calcium, phosphorus, zinc, vitamin B2, and vitamin D.
Duncan DR et al, 2019	Retrospective cohort	137	Mean 78 days	Children <=2 years with laryngeal penetration on VF but no aspiration	Adjustment of liquid viscosity and flow rate; feeding-method instruction	No instruction	Improvement of laryngeal penetration symptoms; hospitalization risk and hospital days for pulmonary disease	Laryngeal penetration improved; total hospital days and pulmonary-disease hospital days decreased.
Zanini M et al, 2017	Before-after study	401	24 weeks	Older facility residents with dysphagia	NUTRICARE program: foods optimized for texture, palatability, and nutrition to meet Italian older adult requirements	Usual diet (before-after study without control)	Body weight, BMI, nutritional status (total protein, albumin, transferrin, lymphocyte count, creatinine)	Body weight and BMI increased; total protein, serum albumin, serum transferrin, and lymphocyte count improved.
Yuan T et al, 2021	RCT	160	24 weeks	Hospitalized patients with dysphagia after cerebral infarction and long-term bed rest	Gastrostomy	Nasogastric tube feeding	Immune function, nutritional status, hepatic/renal function, QoL	Nutritional status did not differ significantly at 3 or 6 months. Depression scale scores and enteral nutrition intolerance were reduced in the gastrostomy group; catheter-related complications increased.
Zheng T et al, 2015	RCT	146	3 weeks	Acute stroke patients with dysphagia	Nasogastric tube feeding	Family nutrition management	Nutritional status, hospital infection, mortality	Enteral nutrition was associated with better nutritional status at 3 weeks and lower hospital infection and mortality; no significant differences remained at 90 days, indicating short-term improvement only.

Author, year	Study design	N	Intervention period	Participants	Intervention	Control	Main outcomes	Findings
Hamidon BB et al, 2006	RCT	22	4 weeks	Acute stroke patients with dysphagia	Gastrostomy	Nasogastric tube feeding	Subcutaneous fat thickness, upper arm circumference, serum albumin	Serum albumin improved significantly more with gastrostomy than nasogastric feeding; anthropometric parameters did not differ significantly.
Dennis M et al, 2006	RCT	321	-	Stroke patients with dysphagia	Gastrostomy	Nasogastric tube feeding	Death; poor outcome	Nasogastric feeding may be better for acute outcomes, but mortality risk did not differ significantly.
Dennis MS et al, 2005	RCT	859	-	Stroke patients with dysphagia	Early tube feeding within 7 days after admission	Usual tube-feeding management	Death; poor outcome	Early tube feeding reduced mortality risk but may increase survival with severe disability.
Norton B et al, 1996	RCT	30	6 weeks	Patients with dysphagia after acute cerebral infarction	Gastrostomy	Nasogastric tube feeding	6-week mortality, nutritional status, discharge rate	Mortality was significantly lower and nutritional status significantly better in the gastrostomy group.
Lee JH et al, 2021	Retrospective study	42	4 weeks	Patients with neurogenic dysphagia	Gastrostomy	Before vs after gastrostomy	Pneumonia incidence	Gastrostomy may reduce pneumonia incidence.
Juan W et al, 2020	RCT	97	-	Patients with post-stroke dysphagia	Intermittent oro-esophageal tube feeding (IOE)	Nasogastric tube feeding	Pneumonia incidence, swallowing function, HAD, comfort score	IOE reduced aspiration pneumonia incidence, improved swallowing function, and produced better comfort and psychological status.
Cintra MT et al, 2014	Prospective observational study	67	At least 6 months follow-up	Patients aged >=60 years with progressive dementia and dysphagia	Alternative nutrition (nasogastric tube or gastrostomy)	Usual oral intake	Mortality and aspiration pneumonia incidence	Alternative nutrition was associated with higher 3- and 6-month mortality and more aspiration pneumonia.
Ickenstein GW et al, 2005	Prospective observational study	66	-	Patients with dysphagia after gastrostomy	Gastrostomy	Gastrostomy withdrawal	Survival	Gastrostomy remaining at discharge and aspiration were associated with lower survival. Patients with better swallowing and cognition had greater likelihood of improved swallowing and survival.
Finestone HM et al, 2001	Prospective observational study	13	3 weeks	Patients admitted within 5 days after stroke onset	PN, EN, dysphagia diet	Oral intake only	Fluid intake	Fluid intake was significantly lower with oral intake only and did not meet required intake.

Author, year	Study design	N	Intervention period	Participants	Intervention	Control	Main outcomes	Findings
Mitchell SL et al, 1998	Prospective observational study	5,266 (tube feeding 551)	56 weeks	Facility residents aged >=65 years with chewing or swallowing impairment	Tube feeding	No tube feeding	Survival, mortality	One-year mortality was higher in the tube-feeding group. Among tube-fed patients surviving 1 year, approximately 25% were tube-free, suggesting temporary use may be possible in reversible conditions.
Szefel J et al, 2016	RCT	50	11 days	Inpatients with refractory cancer cachexia, NRS >=3, and unable to take oral intake	Long-chain plus medium-chain fatty acid intake	Long-chain fatty acid intake	Total protein, albumin, prealbumin, lymphocytes, CRP, survival, mortality	No difference was observed in short-term improvement in nutritional status.
Reyes-Torres CA et al, 2019	RCT	40	12 weeks	Patients aged >=65 years with dysphagia	Viscosity- and texture-modified diet and thickened fluids	Usual diet with equivalent calories	Energy/protein intake, body weight, grip strength, body composition (phase angle)	Energy and protein intake, body weight, and grip strength increased significantly; phase angle also improved.
Heather H Keller et al, 2012	Prospective interrupted time-series pilot study	42	36 weeks	Older adults in hospitals or facilities receiving dysphagia diets	Ready-to-use processed dysphagia diet	Conventional dysphagia diet for 3 months	Body weight, food intake	At 6 months, 74% achieved target body weight; fat intake increased.
Zhu X et al, 2022	RCT	66	12 weeks	Patients with dysphagia after laryngeal cancer surgery	Individual nutrition intervention weekly for 30 min over 3 months; diet adjustment, nutrition assessment, ONS or nasogastric feeding when needed	Health counseling and swallowing training	Swallowing function (VFSE), nutritional status (PG-SGA), QoL (QLQ-C30)	All outcomes improved in both groups, with greater improvement in the nutrition intervention group.
Yan XL et al, 2022	RCT	173	1 week	Hospitalized acute cerebral infarction patients with dysphagia	Dysphagia diet or IOE plus TPF selected according to swallowing and individual status	ONS and family-prepared diet or NG tube plus TPF according to swallowing status	Swallowing function; albumin, total protein, hemoglobin, body composition	Swallowing improvement rate was higher in the intervention group, and nutritional indices such as serum albumin and total protein improved.
Iwamoto M et al, 2014	Retrospective study	70	Mean 45.5 days	Hospitalized acute-care patients with dysphagia	NST intervention: nutrition assessment, enhanced nutrition, swallowing assessment	-	Mortality; swallowing function improvement	Patients with better nutritional status had greater swallowing-function improvement. Energy intake <22 kcal/kg/day was associated with poor swallowing recovery and prognosis.

Author, year	Study design	N	Intervention period	Participants	Intervention	Control	Main outcomes	Findings
Wright L et al, 2008	Intervention group prospective observation; control group retrospective observation	46	3 days	Patients aged >=65 years with dysphagia receiving texture-modified or thick liquid diets	Feeding support according to level, including meal assistance	Usual care	Dietary intake	Energy and protein intake increased significantly in the feeding-support group.
Manabe T et al, 2022	Retrospective observational study	1,061	>=5 days	Patients managed with alternative nutrition who died in hospital	EN, EN+PN, or PN	-	Survival days, 180- and 360-day mortality	The PN group had shorter survival and higher mortality than EN or EN/PN groups.
Furuta M et al, 2019	Retrospective cohort	51	-	Esophageal cancer patients receiving EN or TPN within 2 weeks after chemoradiotherapy initiation	EN	TPN	Nutritional status, SMA, adverse events	Serum albumin improved significantly in the EN group, with trends toward less hematologic toxicity and shorter hospitalization.
Sitzmann JV, 1990	Retrospective observational study	90	-	Hospitalized patients with dysphagia	EN or PN	None	Aspiration pneumonia incidence, mortality	The EN group had complications such as aspiration and high mortality.
Lim ST et al, 1981	Randomized trial	24	3-4 weeks	Patients with esophageal cancer and dysphagia	TPN	Gastrostomy	Nutritional status and complications	TPN achieved earlier improvement in nitrogen balance and weight gain.
Mamun K et al, 2005	Prospective observational study	122	-	Older home-care patients with dysphagia	Nasogastric tube feeding	Oral intake or refusal of tube	Aspiration pneumonia incidence, mortality	Aspiration pneumonia and mortality were higher in the NG group, but poorer cognition and ADL may have influenced prognosis.
Shimizu A et al, 2021	Prospective cohort	110	-	Hospitalized patients aged >=65 years with sarcopenic dysphagia	High-energy diet >=30 kcal/IBW/day	Usual diet <=30 kcal/IBW/day	FILS at discharge; FIM gain >=22	Swallowing function (FILS) and ADL (FIM) improved significantly in the intervention group.

Author, year	Study design	N	Intervention period	Participants	Intervention	Control	Main outcomes	Findings
Taylor KA, 2006	Crossover trial	37	4 days x2	Facility residents aged >=65 years receiving dysphagia diets	Five meals/day with same total energy as three meals/day	Three meals/day	Mean energy intake	Energy intake did not differ significantly, but fluid intake was significantly higher with five meals.
Ueshima J et al, 2025	Intervention study	62	1 month	Acute stroke patients aged >=65 years with dysphagia	Texture-modified diet fortified while maintaining meal volume	Conventional diet	Energy intake; ADL	Energy intake increased by approximately 200 kcal/day without increasing meal volume, and functional recovery and ADL improved.

Supplementary Table 8-1. Structured abstracts (BQ3)

Author, year	Study design	N	Intervention period / participants	Intervention	Control	Main outcomes	Findings
Wang T et al, 2021	Retrospective observational study	113	Patients with esophageal cancer and dysphagia	Gastrostomy	Self-expanding stent for oral intake	Serum albumin, body weight	Gastrostomy was associated with maintenance of nutritional status, fewer complications, and longer survival compared with stent placement.
Hamidon BB et al, 2006	RCT	22	8 weeks	Hospitalized patients with dysphagia after cerebral infarction	Gastrostomy	Nasogastric tube feeding	Serum albumin, triceps skinfold, upper arm circumference
Jukic Peladic N et al, 2023	Retrospective study	267	Older outpatients	-	-	BMI	Dysphagia was observed in >96% of participants, and 22.1% of those had malnutrition. BMI improved in 68.4% of malnourished patients receiving nutrition therapy.
Kimyagarov S et al, 2013	Retrospective observational study	117	Adults aged >=65 years	Groups without dysphagia, with dysphagia/oral intake, and with gastrostomy	-	BMI, nutritional intake	Dysphagia was associated with low BMI, negative nitrogen balance, low fat mass, and low energy/protein intake.
Scalabrino N, 2010	Cross-sectional observational study	38	Tracheostomized patients after cardiac surgery	Swallow screening using water swallow and blue-dye tests	-	Aspiration, dehydration, malnutrition	Early swallowing screening was useful for early identification of high aspiration risk and prevention of serious complications.
Jeoffray D et al, 2018	Cross-sectional observational study	222	Stroke patients with dysphagia	-	-	BMI, triceps skinfold, upper arm circumference, swallowing function	Malnutrition was frequent and increased further after admission. Women and patients with low admission weight or skinfold thickness required intensified nutrition management.
Zanini M et al, 2017	Before-after study	479	Facility residents with dysphagia	Individualized diet program adjusting density, viscosity, texture, and particle size	-	BMI, biochemical indices	BMI and body weight increased, and biochemical indices improved.
Rofes L et al, 2014	Cross-sectional observational study	134	Patients with dysphagia related to aging, stroke, or neurodegenerative disease	-	-	EAT-10, swallowing function	Useful for screening and clinical assessment of dysphagia.

Author, year	Study design	N	Intervention period / participants	Intervention	Control	Main outcomes	Findings
Taguchi K et al, 2022	Multicenter cohort study	207	Patients aged >=20 years with dysphagia	-	-	FILS, FIM, discharge destination	Sarcopenic dysphagia was frequent in convalescent hospitals; severely malnourished patients had poor swallowing-function prognosis.
Akutsu Y et al, 2012	Cross-sectional observational study	18	4 weeks	Patients with esophageal cancer receiving chemotherapy	TPN	TPN plus trace elements	Serum trace-element concentrations
Alvarez-Larruy M et al, 2023	Cross-sectional observational study	45	Acute stroke patients	-	-	Swallowing function, nutritional status	After acute stroke, patients with dysphagia frequently had reduced spontaneous swallowing, malnutrition, and dehydration and were at high risk of respiratory complications.
Zheng T et al, 2015	RCT	146	28 days	Acute stroke patients	Early enteral nutrition	Family nutrition management	Nutritional status, mortality
Leibovitz A et al, 2004	Cross-sectional observational study	45	Residents of a geriatric medical center	-	-	Nutritional status, biochemical indices	Uric acid concentration was significantly lower in tube-fed than orally fed residents.
Manas-Martinez AB et al, 2018	Retrospective cohort	90	Patients admitted to internal medicine wards	-	-	Malnutrition, mortality	EAT-10 identified 56.7% as at risk of dysphagia; malnutrition and mortality were significantly higher. Dysphagia risk by EAT-10 was an independent predictor of death.
Carrion S et al, 2017	Cross-sectional observational study	133	Older adults aged >=70 years with dysphagia	-	-	Nutritional status	Malnutrition and sarcopenia were highly prevalent among older patients with dysphagia.
Lin LC et al, 2003	Quasi-experimental parallel cluster design	61	Institutionalized patients with stroke	Swallowing training	-	Swallowing function, nutritional status	Swallowing training improved swallowing function and nutritional status in institutionalized stroke patients.
Limpuangthip N et al, 2022	Cross-sectional study	5 years	Adults aged >=60 years receiving dental care	-	-	Swallowing function, malnutrition risk	Those with swallowing problems had significantly lower tongue pressure; subjective swallowing problems and low tongue pressure were both significantly associated with malnutrition risk.
Cristofaro MG et al, 2021	Prospective observational study	60	Cancer patients	-	-	Body weight, nutritional status	In cancer patients, dysphagia was strongly associated with malnutrition and weight loss.
Gandolfo C et al, 2019	Multicenter observational study	-	Patients with post-stroke dysphagia	-	-	Persistent dysphagia	Low BMI (<20) and high mRS (>=3) were important predictors of persistent dysphagia.

Author, year	Study design	N	Intervention period / participants	Intervention	Control	Main outcomes	Findings
Mateos-Nozal J et al, 2022	Prospective observational study	447	Patients aged >=70 years	-	-	Dysphagia management adherence and short-term prognosis	In older patients with dysphagia, adherence to dysphagia management after education was associated with improved short-term prognosis.

Supplementary Table 9-1. Structured abstracts (CQ6)

Author, year	Study design	N	Intervention period	Participants	Intervention	Control	Main outcomes	Findings
Krekeler N et al, 2023	RCT	19	12 weeks	Acute stroke patients with dysphagia	Tongue resistance training using SwallowSTRONG device	Usual dysphagia rehab	Strength of swallowing-related muscles; swallowing function	Tongue strength increased and oral intake improved.
Jensen D et al, 2022	RCT	90	6 weeks	Community-dwelling patients with dysphagia	CTAR three times/day	Usual dysphagia rehab with environmental and diet modification	V-VST, MEOF-II, Dysphagia Handicap Index, Barthel 20, EQ-5D, energy/protein intake, BMI, grip, FOIS	Swallowing function and HRQoL improved.
Tuomi L et al, 2022	RCT	61	8 weeks	Community-dwelling patients after radiotherapy/chemotherapy for head and neck cancer	Shaker exercise three times/day	Usual dysphagia rehab	PAS, SPS, EORTC QLQ-H&N35	Only health-related QoL improved.
Dotevall H et al, 2022	RCT	61	8 weeks	Community-dwelling patients after chemoradiotherapy for head and neck cancer	Shaker exercise three times/day	Written, video, and oral instructions	PAS, EAT-10, SPS	Only EAT-10 improved significantly.
Wang T et al, 2022	Single-blind RCT	37	4 weeks	Inpatients with post-stroke dysphagia	Tongue-pressure resistance training, 20 min/day, 5 days/week	Usual dysphagia rehab	FCM, OMFS, MTP, MSS, YPRSRS, PAS	Swallowing and oral motor function improved; tongue strength increased.
Claus I et al, 2021	Double-blind RCT	50	4 weeks	Outpatients with Parkinson disease	EMST, 5 days/week, 5 x 5 sets	Sham device training	FEES, SWAL-QOL, SDQ	Swallowing function improved and pharyngeal residue decreased.
Choi JB et al, 2020	RCT	21	6 weeks	Inpatients with post-stroke dysphagia 1-5 months after onset	CTAR with isometric and isotonic exercise, 5 times/week	Head-lift training	Swallowing function, suprahyoid thickness, hyoid movement, Borg RPE	Both groups increased suprahyoid muscle mass and hyoid movement; CTAR had significantly lower perceived exertion.
Liaw MY et al, 2020	RCT	21	6 weeks	Outpatients with post-stroke dysphagia	Respiratory training with inspiratory pressure 30-60% MIP and expiratory pressure 15-75% MEP, 1-2 times/day, 5 days/week	Usual dysphagia rehab	FOIS	FOIS improved significantly.
Kim HH et al, 2019	RCT	30	6 weeks	Inpatients with post-stroke dysphagia	CTAR 5 days/week	Usual dysphagia rehab	PAS, FOIS, transition to oral intake	PAS and FOIS improved significantly; transition to oral intake was 25% vs 15%.

Author, year	Study design	N	Intervention period	Participants	Intervention	Control	Main outcomes	Findings
Park HS et al, 2019	Double-blind RCT	30	4 weeks	Inpatients with post-stroke dysphagia	Effortful swallowing training	Usual dysphagia rehab	Tongue strength, VFSS, VDS	Oral-phase swallowing function improved; anterior and posterior tongue strength increased.
Hagglund P et al, 2019	Cluster RCT	36	5 weeks	Older patients with dysphagia	Oral neuromuscular training (IQoro)	Usual dysphagia rehab	Water swallow test; swallowing-related QoL	Swallowing speed and liquid swallowing function improved.
Park JS et al, 2019	RCT	46	4 weeks	Inpatients with post-stroke dysphagia	Game-based CTAR plus usual rehab	Shaker exercise plus usual rehab	VDS, PAS, FOIS, adherence-related assessment	No significant difference in swallowing improvement; motivation, interest, and enjoyment were higher, and effort and fatigue lower.
Hwang NK et al, 2019	Double-blind RCT	25	4 weeks	Outpatients with post-stroke dysphagia	Tongue stretching exercises plus TDT	Usual dysphagia rehab	VFSS, VDS, tongue motility	Tongue movement, bolus formation, tongue-palate contact, and bolus transit time improved significantly.
Moon JH et al, 2018	RCT	16	8 weeks	Subacute stroke patients with dysphagia	Anterior/posterior isometric tongue-strength and accuracy training at 50%, 75%, 100% maximum pressure	Usual dysphagia rehab	Strength of swallowing-related muscles, swallowing function, SWAL-QOL	Tongue function/strength and swallowing-related QoL improved significantly.
Wakabayashi H et al, 2018	Cluster RCT	104	12 weeks	Community-dwelling adults aged >=65 years with dysphagia	Resistance training of swallowing muscles	Pamphlet on dysphagia rehab	EAT-10, tongue pressure, MNA-SF, Barthel Index	No improvement in swallowing function was observed in either group.
Ploumis A et al, 2018	RCT	70	12 weeks	Patients with dysphagia in rehabilitation facilities	Cervical isometric exercises	Usual dysphagia rehab	Swallowing function	Swallowing function improved significantly.
Park JS et al, 2018	Single-blind RCT	25	4 weeks	Patients with post-stroke dysphagia within 12 months	CTAR	Usual dysphagia rehab	FDS, PAS	FDS and PAS improved significantly.
Rachel M et al, 2009	RCT	11	6 weeks	-	Shaker exercise three times/day	Usual dysphagia rehab	Swallowing function	Thyrohyoid contraction was strengthened.
Don KK et al, 2015	RCT	26	6 weeks	-	Neck flexion exercise based on proprioceptive neuromuscular facilitation, 30 min/session, 3 days/week	Shaker exercise, 30 min, 3 days/week	Swallowing function	Pharyngeal transit time, pharyngeal residue, and aspiration improved significantly.
Shaker R et al, 2002	RCT	27	6 weeks	Community-dwelling patients with dysphagia	Shaker exercise	Sham exercise	FOAMS	Upper esophageal sphincter opening and laryngeal elevation increased.

Author, year	Study design	N	Intervention period	Participants	Intervention	Control	Main outcomes	Findings
Park JS et al, 2015	RCT	29	6 weeks	Community-dwelling patients with dysphagia	Tongue-strength training using IOPI, five sets of 10 repetitions daily, 5 times/week	Usual dysphagia rehab	Swallowing-related muscle mass/strength; swallowing function	Tongue strength and swallowing function improved.
Bautmans I et al, 2008	RCT	16	3 weeks	Institutionalized older patients with dysphagia	Cervical mobilization	No intervention	Swallowing function	Swallowing function improved.
Krajczy E et al, 2019	RCT	60	15 days	Patients with dysphagia after cerebral infarction	Composite training 60 min/day for 15 days	Usual dysphagia rehab	Voice quality, number of swallows, swallowing time	Swallowing reflex improved.
Park JS et al, 2020	Double-blind RCT	40	5 weeks	Community-dwelling patients with post-stroke dysphagia within 6 months	Resistive jaw-opening exercise	Training with sham device	VFSS, PAS, FOIS	PAS improved during liquid intake.
Sayaca C et al, 2020	RCT	50	6 weeks	Community-dwelling adults aged >=65 years with dysphagia	PNF swallowing exercises	Head-lift (Shaker) exercises	EAT-10, water swallowing test, sEMG, swallowing performance parameters	Swallowing function improved in both groups; suprahyoid muscle strength increased significantly in the intervention group.
Logemann JA et al, 2009	RCT	19	6 weeks	Community-dwelling patients with dysphagia after stroke or chemoradiotherapy for head and neck cancer	Shaker exercise four times/day	Usual dysphagia rehab	Swallowing function	Liquid aspiration decreased and upper esophageal sphincter opening increased.
Park JS et al, 2016	Single-blind RCT	33	4 weeks	Inpatients with post-stroke dysphagia	EMST at 70% MEP, 5 sets/day, 5 days/week	Training with sham device	PAS, FOIS, sEMG	Swallowing during liquid intake improved; suprahyoid activity increased.
Steele CM et al, 2016	RCT	14	8-12 weeks; 24 sessions	Inpatients with post-stroke dysphagia	Tongue-pressure profile training	Tongue-pressure strength and accuracy training	Tongue strength, VFSS, PAS, NRRS	Both groups improved tongue strength during swallowing and reduced pharyngeal residue after liquid intake.
Koyama Y et al, 2017	RCT	16	6 weeks	Inpatients with post-stroke dysphagia	Modified jaw-opening exercise at 80% MVC	Sham isometric jaw-closing exercise	Swallowing function	Anterior hyoid movement increased.
Kim HD et al, 2017	RCT	41	4 weeks	Inpatients with post-stroke dysphagia	Tongue-to-palate resistance training plus usual rehab	Usual dysphagia rehab	ATS, PTS, VDS, PAS	Swallowing function improved and tongue strength increased.
Choi JB et al, 2020	RCT	21	6 weeks	Inpatients with post-stroke dysphagia 1-5 months after onset	CTAR	Head-lift training	Swallowing function, suprahyoid thickness, hyoid movement, Borg RPE	Both groups increased suprahyoid muscle mass and hyoid movement; CTAR had lower perceived exertion.

Author, year	Study design	N	Intervention period	Participants	Intervention	Control	Main outcomes	Findings
Eom MJ et al, 2017	RCT	26	4 weeks	Subacute stroke patients with dysphagia	EMST using a portable expiratory muscle trainer, 25 repetitions/day, 5 days/week	Sham device without load	PAS, VDS	Swallowing function improved.
Park JS et al, 2017	RCT	37	4 weeks	Inpatients with post-stroke dysphagia	Shaker exercise 5 times/week	Usual dysphagia rehab	PAS, hyoid movement	Upward hyoid movement improved and liquid aspiration decreased.
Gao J et al, 2017	RCT	90	10.5 weeks	Inpatients with post-stroke dysphagia	CTAR 30 repetitions x3 sets/day, 7 days/week for 42 days	Usual dysphagia rehab	Swallowing function	Swallowing function improved significantly with CTAR and head-lift training.

Note: Rehabilitation is abbreviated as rehab where used.

Supplementary Table 10-1. Structured abstracts (CQ7)

Author, year	Study design	N	Intervention period	Participants	Intervention	Control	Main outcomes	Findings
Turra GS et al, 2021	RCT	32	Up to 10 days	Patients with dysphagia hospitalized in acute care	Indirect swallowing training combined with stepwise oral intake training and diet modification	SHAM stimulation program (placebo therapy)	Dysphagia function	FOIS score improved, swallowing-function score improved, transition to oral intake increased, and oral intake started earlier.
Zhu X et al, 2022	RCT	66	3 months	Patients with laryngeal cancer after surgery and radiotherapy	Usual dysphagia rehab plus nutrition intervention	Usual dysphagia rehab only	Swallowing function, PG-SGA nutritional assessment, QLQ-C30 QoL	Swallowing function, nutritional status, and QoL improved significantly in the intervention group.
Kikutani T et al, 2006	RCT	14	4 months	Malnourished older facility residents	Oral function training plus nutrition intervention	Nutrition intervention only	Serum albumin	The nutrition-only group did not improve significantly; the oral function training plus nutritional supplementation group increased significantly.

Note: Rehabilitation is abbreviated as rehab where used.

Supplementary Table 11-1. Structured abstracts (CQ8)

Author, year	Study design	N	Intervention period	Participants	Intervention	Control	Main outcomes	Findings
Seirup SB et al, 2023	RCT	10	Not described	Community-dwelling patients with dysphagia whose tube-feeding intake was <50% and energy intake was below estimated requirements	Individualized nutrition counseling including diet texture modification based on swallowing assessment, supported by a dietitian	Diet-texture recommendation only	Energy intake by 3-day dietary record	Individualized nutrition counseling did not significantly affect energy intake or other outcomes, but improvement trends were observed.
Zhu X et al, 2022	RCT	66	3 months	Patients with laryngeal cancer after surgery and radiotherapy	Usual dysphagia rehab plus nutrition intervention	Usual dysphagia rehab only	Swallowing function, PG-SGA nutrition assessment, QLQ-C30 QoL	Swallowing function, nutritional status, and QoL improved significantly in the intervention group.
Reyes-Torres CA et al, 2019	RCT	40	12 weeks	Patients aged >=65 years with dysphagia	Texture- and viscosity-modified diet and thickened fluids	Usual diet with equivalent calories	Energy/protein intake, body weight, grip strength, body composition (phase angle)	Energy and protein intake, body weight, and grip strength increased significantly, and phase angle improved.
Yan XL et al, 2022	RCT	173	1 week	Hospitalized acute cerebral infarction patients with dysphagia	Dysphagia diet or IOE plus TPF selected according to swallowing and patient status	ONS and family-prepared diet or NG tube plus TPF according to swallowing status	Swallowing function; serum albumin, total protein, hemoglobin, body composition	Swallowing-function improvement was higher in the intervention group, and nutritional indices such as serum albumin and total protein improved.
Zheng T et al, 2015	RCT	146	3 weeks	Acute stroke patients with dysphagia	Nasogastric tube feeding	Family nutrition management	Nutritional status, nosocomial infection, mortality	Enteral nutrition was associated with better nutritional status at 3 weeks and lower infection and mortality; no significant difference remained at 90 days, suggesting short-term benefit only.
Silander E et al, 2012	RCT	134	2 years	Patients with advanced head and neck cancer	Early enteral nutrition by prophylactic PEG	Usual nutrition management	Body weight, presence of dysphagia, energy intake	Prophylactic PEG did not produce significant differences in nutritional intake or weight change.
Dennis MS et al, 2005	RCT	859	-	Stroke patients with dysphagia	Early tube feeding within 7 days after admission	Usual tube-feeding management	Death; poor outcome	Early tube feeding reduced mortality risk but may increase survival with severe disability.

Author, year	Study design	N	Intervention period	Participants	Intervention	Control	Main outcomes	Findings
Germain I et al, 2006	RCT	17	12 weeks	Facility residents aged >=65 years with dysphagia, BMI <24 or >=7.5% weight loss in 3 months	Texture-modified minced or puree diet, thickened fluids as needed, supplements, individualized menu cycle	Usual menu	Macro- and micronutrient intake, body weight, BMI	Body weight increased, and intake of energy, protein, fat, saturated and monounsaturated fatty acids, potassium, magnesium, calcium, phosphorus, zinc, vitamin B2, and vitamin D increased.

Note: Rehabilitation is abbreviated as rehab where used.

Supplementary Table 12-1. Structured abstracts (FRQ1)

Author, year	Study design	N	Participants	Intervention	Control	Main outcomes	Findings
Liu HY et al, 2014	Secondary analysis of an RCT (24-month follow-up)	252	Older adults with proximal femoral fracture who had malnutrition or risk of malnutrition at discharge	Comprehensive care including depression management, fall prevention, nutrition management, and 1-year home rehab	Two control groups: interdisciplinary care with 4-month home rehab; usual care without home intervention	Nutritional-status recovery rate	Comprehensive care significantly promoted recovery of nutritional status.
Pison CM et al, 2011	Prospective RCT	122	Patients with malnutrition and chronic respiratory failure	Endurance cycling 3-5 times/week, strengthening, oral testosterone undecanoate and ONS for 90 days	Home health education only using a pulmonary rehab booklet for 90 days	6MWD; secondary outcomes: BMI, FFMI, strength, exercise tolerance, QoL, 15-month survival	Multimodal nutrition rehab did not significantly improve 6MWT, but improved body composition, strength, and exercise tolerance; among adherent patients, survival improved and women had QoL improvement.

Note: Rehabilitation is abbreviated as rehab where used.

Abbreviations: CRF, chronic respiratory failure; ONS, oral nutritional supplements; BMI, body mass index; FFMI, fat-free mass index; QoL, quality of life; 6MWT, 6-minute walk test. Effect sizes and statistical significance are based on the original articles.

Supplementary Table 14-1. Structured abstracts (CQ9)

Author, year	Study design	N	Participants	Intervention	Control	Main outcomes	Findings
Baumgartner A et al, 2021	Randomized controlled trial	881	Hospitalized patients with NRS2002 score >=3	Individualized nutrition management and dietary counseling by registered dietitians	Standard hospital diet management	30-day mortality, 180-day mortality, BI, QoL (EQ-5D)	30-day and 180-day mortality decreased; BI and QoL indices improved.
Otsuki I et al, 2020	Randomized controlled trial	94	Hospitalized acute stroke patients aged >=65 years with MUST score >=2 or albumin <=3.0 g/dL	Dietitians calculated requirements using the Harris-Benedict equation and provided diet	Dietitians calculated requirements using 20-30 kcal/kg and provided diet	Total FIM, motor FIM, cognitive FIM, upper arm and thigh circumference	Total FIM and motor FIM improved.
Zhou C et al, 2023	Randomized controlled trial	90	Hospitalized patients aged >=65 years with heart failure	Patient education using food exchange method, individualized nutrition management, and post-discharge interviews	Usual patient education and telephone follow-up after discharge	Body composition, 6MWD, psychosomatic function	Body composition, 6MWD, and psychosomatic function improved.
Andersson J et al, 2017	Randomized controlled trial	100	Adults in rehab facilities with NRS2002 score >=3	Individualized nutrition management by dietitian	Usual nutrition management	QoL (EQ-5D), appetite (DRAQ)	No change was observed in QoL or appetite indices.
Feldblum I et al, 2010	Randomized controlled trial	168	Acute medical ward inpatients aged >=65 years with MNA-SF score <10	Individualized nutrition management based on nutritional status and NSI	Usual nutrition management	180-day mortality, BI, GDS, MMSE	180-day mortality decreased; BI, GDS, and MMSE did not change.
Persson M et al, 2007	Randomized controlled trial	54	Older inpatients with MNA-SF score <=10	Individualized nutrition counseling by dietitian and prescription of nutritional supplements	Brief written dietary advice	Grip strength, MMSE, ADL (Katz Index), QoL (SF-36)	ADL improved; grip strength, MMSE, and QoL did not change.

Note: Rehabilitation is abbreviated as rehab where used.

Abbreviations: BI, Barthel Index; QoL, quality of life; EQ-5D, EuroQol 5-Dimension; FIM, Functional Independence Measure; DRAQ, Disease-Related Appetite Questionnaire; NSI, Nutrition Screening Initiative; GDS, Geriatric Depression Scale; MMSE, Mini-Mental State Examination; SF-36, Short Form-36 Health Survey.

Supplementary Table 15-1. Structured abstracts (CQ10)

Author, year	Design	N	Participants	Intervention	Control	Outcomes	Findings
Zhou et al, 2023	Randomized controlled trial	90	Older patients with chronic heart failure	Individualized nutrition prescription and dietary intervention training based on dietitian assessment	General nutrition education and dietary guidance by cardiac rehab nurse	SMM, AMC, bilateral lower-limb muscle mass, 6MWD, METs	The intervention group had significantly higher body protein and skeletal muscle mass and significantly lower body fat percentage than controls.
Lin et al, 2021	Randomized controlled trial	56	Older adults with sarcopenia	Dietitian counseling plus protein supplement	Guideline-based high-protein food recommendations	Appendicular skeletal muscle index, grip strength, gait speed	After 12 weeks, grip strength improved in the control group, gait speed improved in the intervention group, and AMMI improved in both groups.
Hsieh et al, 2019	Randomized controlled trial	319	Community-dwelling older adults	1) Exercise prescription; 2) individualized dishware set and supplements by dietitian; 3) combination of 1 and 2	Usual medical care	Frailty score, grip strength, gait speed, upper/lower body flexibility	All three intervention groups had significantly greater change in grip strength than controls. Gait speed and upper/lower body flexibility also improved significantly in multiple intervention groups.
Yang et al, 2019	Randomized controlled trial	82	Older patients with malnutrition and pneumonia	Dietitian support, individualized nutrition plan, and post-discharge dietary guidance	Standard oral nutritional supplements	AMC, AMA	No difference in anthropometric measures between intervention and control groups.
Rodriguez-Manas et al, 2019	Randomized controlled trial	964	Older adults with type 2 diabetes	Resistance training, nutrition education intervention, and diabetes-treatment optimization training	Usual medical care	SPPB, BI, Lawton scale, EQ-5D-5L	The intervention group had significantly greater 26-week change in SPPB than controls.

Note: Rehabilitation is abbreviated as rehab where used.

Abbreviations: AMC, arm muscle circumference; AMA, arm muscle area; BI, Barthel Index; SMM, skeletal muscle mass; SPPB, Short Physical Performance Battery.

Supplementary Table 16-1. Structured abstracts (CQ11)

Author, year	Study design	N	Participants	Intervention	Control	Main outcomes	Findings
Shiraishi et al, 2019	Retrospective cohort study	1,056	Older inpatients in convalescent rehab wards	Professional oral management by dental hygienist at least twice weekly	Usual care	FIM score, home discharge rate, length of stay, mortality	All outcomes were significantly better in the intervention group.
Yoshimi et al, 2021	Prospective observational study	100 (50 per group)	Older pneumonia patients with dysphagia	Oral management by dental professionals	No intervention	Oral intake (FOIS), length of stay	Proportion able to take oral intake increased and length of stay decreased.
Fujita et al, 2023	Prospective single-center study	214	Acute stroke patients	Dental oral care within 72 hours plus early mobilization rehab	Usual care	Aspiration pneumonia, death/reintervention	Pneumonia and reintervention rates were significantly lower in the intervention group.
Sakai et al, 2024	Prospective observational study	98	Inpatients from acute to convalescent phases after stroke	Dental intervention for oral care and OHAT score assessment	No intervention	OHAT score, possibility of oral intake	OHAT score improved; improvement was larger in the oral-intake group.
Shiraishi et al, 2019 (separate study)	Prospective cohort study	Not specified (same data series)	Inpatients in convalescent rehab wards	Oral status assessment by ROAG score at admission	None (predictor analysis)	FIM, home discharge rate, length of stay, mortality	ROAG score was independently associated with all outcomes.

Note: Rehabilitation is abbreviated as rehab where used.

Supplementary Table 17-1. Structured abstracts (CQ12)

Author, year	Study design	N	Intervention period	Participants	Intervention	Control	Main outcomes	Findings
Willoch et al, 2012	RCT	77	During hospitalization	General-hospital rehab ward; mean age 73.5 +/- 12.2 vs 76.8 +/- 11.7	Pharmacist participation in the rehab team	Multidisciplinary team without pharmacist	Types and frequency of DRP	DRPs at discharge and after discharge were significantly fewer in the intervention group.
Marra C et al, 2012	RCT	139	-	Patients aged >=50 years with knee pain	Pharmacist interview, patient education, medication review, referral to physiotherapist-guided exercise, and communication of interview record to physician	OA education pamphlet	Quality-of-care pass rate for OA, function (WOMAC, LEFS), QoL (PAT-5D, HUI3)	Quality-of-care pass rate was significantly higher in the intervention group. WOMAC global, pain, and function scores improved significantly at 3 and 6 months; LEFS improved at 6 months. Pain-related HUI3 and PAT-5D domains improved.
Vasterad et al, 2021	RCT	70	4 weeks	Adult COPD patients receiving respiratory rehab	Patient education and pharmaceutical care	Standard care	QoL (WHOQOL-Bref)	QoL scores improved significantly in the intervention group overall and across physical, psychological, socioeconomic, and environmental domains (all P<0.0001).
Horita et al, 2018	RCT	103	6 months	Community-pharmacy patients with chronic disease (hypertension, diabetes, dyslipidemia); older adults	Medication counseling plus frailty-prevention exercise leaflet, home exercise encouragement, and repeated follow-up at pharmacy visits	Medication counseling only	Muscle mass, five-times sit-to-stand time, body weight, body fat, BMI, estimated bone mass	Muscle mass tended to increase but not significantly. Other continuous outcomes did not differ; the proportion with faster five-times sit-to-stand improved significantly (65.2% vs 29.2%, P=0.00563).
Ee C et al, 2019	RCT	200	During hospitalization	Rehab hospital; mean age 72.8 +/- 10.7 vs 68.6 +/- 10.0	Deprescribing process by pharmacist and physician	Usual physician assessment and medication adjustment without pharmacist suggestions	1-month medication cost, change in number of drugs, potential adverse effects of deprescribing	Costs and reductions in medication number did not differ. Acid suppressant deprescribing was most common; analgesics were restarted in 2/7 cases because of pain recurrence.
Petkova VB, 2009	RCT	86	4 months	Patients with arthritis	Education on causes/exacerbating factors, pain management, physical activity, self-management/prevention, pharmacotherapy, and adverse effects	Usual treatment	Pain self-monitoring, adherence, visits, adverse drug events, satisfaction, pain-related QoL	The intervention group showed significant improvements in pain, adherence, unplanned medical assistance, GP visits, adverse events, and satisfaction; QoL did not differ between groups, but several domains improved within the intervention group.

Author, year	Study design	N	Intervention period	Participants	Intervention	Control	Main outcomes	Findings
Anchah L et al, 2017	RCT	112	12 months	Patients with acute coronary syndrome receiving cardiac rehab	Phase I inpatient pharmacist counseling and pharmaceutical care; Phase II outpatient education on disease, medication, diet, lifestyle, and exercise intensity	Usual care	SF-36	Mean change from baseline to 12 months was higher in intervention groups, with phase I highest for physical functioning, role physical, vitality, and social functioning, and phase II higher for physical functioning, bodily pain, general health, and mental health.
Casper et al, 2019	RCT	40	3 months	Patients with acute coronary syndrome receiving cardiac rehab	Pharmacist services in addition to standard cardiologist care: admission assessment, DRP identification/management, education, adherence follow-up	Standard cardiologist care	DRP, adherence, patient knowledge, SF-36, heart rate, blood pressure, LDL, total cholesterol, fasting glucose	Intervention reduced cardiovascular risk markers, improved LDL target achievement, knowledge, adherence, and all SF-36 domains, and reduced total DRPs.
Henriksen BT et al, 2023	Before-after study	108	Admission to 6 weeks post-discharge	Patients aged >=18 years hospitalized with femoral fracture; mean age 84	Pharmacist pathway including medication reconciliation/review at admission, during stay, rehab admission, and 3-6 weeks after discharge, plus medication information in discharge summary	Usual care without pharmacist support	Quality of medication information in discharge summary, PIM at discharge, guideline-concordant pharmacotherapy, readmission/death at 30 and 90 days	Discharge summary quality was higher; PIM at discharge was lower; prophylactic laxatives with opioids and osteoporosis pharmacotherapy were more frequent; readmission or mortality did not differ.
Hohman et al, 2009	Before-after study	255	12 months	Patients with TIA or ischemic stroke	Enhanced care in community pharmacy: medication review, counseling, identification and resolution of medication-related problems	Standard care	QoL (SF-36), adherence to secondary prevention medications, satisfaction	QoL did not change significantly in the intervention group but declined in controls. Guideline-based secondary prevention adherence did not differ; satisfaction with individualized pharmacist counseling was higher.
Freyer et al, 2018	Before-after study	313	During hospitalizatio n	Rehab hospital; median age 80 vs 82 years	Dual medication intervention: medication review and physician communication during stay; improved discharge summaries for medication-related information	Standard treatment	Number of patients with DRP at discharge, discharge summary quality	Patients with DRP at discharge decreased significantly. Discharge summary quality improved for active ingredients, brand names, explanations of medication changes, visualization of discharge medications, and recommended duration for short-term drugs.

Note: Rehabilitation is abbreviated as rehab where used.

Abbreviations: BMI, Body Mass Index; CADE, Comprehensive Assessment of Disability; CI, confidence interval; DRP, drug-related problem; HUI3, Health Utilities Index Mark 3; LEFS, Lower Extremities Function Scale; LDL, low-density lipoprotein cholesterol; OA, osteoarthritis; PAT-5D, Paper Adaptive Test-5D; PIM, potentially inappropriate medications; SF-36, MOS Short-Form 36-Item Health Survey; TIA, transient ischemic attack; WOMAC, Western Ontario and McMaster Universities Osteoarthritis Index.

Supplementary Table 18-1. Structured abstracts (BQ5)

Author, year	Study design	N	Intervention period	Participants	Intervention	Control	Main outcomes	Findings
Martone AM et al, 2017	Cohort study	394	Feb-May 2014	Italy; patients aged >=65 years admitted to an acute geriatric medical ward (79.6 +/- 6.4 years)	Evaluation at discharge	Before treatment/admission baseline	Sarcopenia (EWGSOP1)	Among 394 patients without sarcopenia at admission, 58 (14.7%) developed new sarcopenia at discharge. Incident cases were older and had lower BMI, lower SMI, ADL disability, cognitive impairment, and longer bed rest. ADL disability and bed rest were independent factors; higher BMI and admission SMI were protective.
Malafarina V et al, 2019	Cohort study	187	Jan 2012-Dec 2015	Spain; patients aged >=65 years admitted for rehab after hip fracture surgery (85.2 +/- 6.3 years)	Evaluation at discharge	Baseline at admission	Sarcopenia (EWGSOP)	Among 97 patients without sarcopenia at admission, 54 developed sarcopenia during hospitalization; incidence was higher in men. Related factors were low BMI, low grip strength, and low SMI. Sarcopenia at discharge and low grip strength were associated with mortality.
Yuenyongchaiwat K et al, 2020	Cohort study	153	2018	Thailand; patients aged 35-80 years admitted for open heart surgery (mean age 61.1 +/- 11.5 years)	Before discharge	Baseline at admission	Postoperative sarcopenia (AWGS) incidence	Preoperative sarcopenia was present in 43/160 (26.9%) and new postoperative sarcopenia developed in 32 (20.9%). Longer mechanical ventilation and length of stay, age >=55 years, low BMI (<23 kg/m2), and diabetes were associated factors.
Welch C et al, 2022	Cohort study	81	May 2019-Apr 2021	UK; adults aged >=70 years hospitalized for elective colorectal surgery, emergency abdominal surgery, or acute infection (mean 79.2 years)	Day 7 (+/-2) after admission/surgery; week 13 (+/-1)	Baseline at admission	Post-admission/postoperative sarcopenia incidence	Among 25 without baseline sarcopenia, 5 (20.0%) developed sarcopenia by day 7 and 2 (8.0%) showed possible sarcopenia. BATT and gait speed decreased by day 7 and tended to improve by week 13, with wide individual variation.
Nguyen M et al, 2022	Cohort study	79	Jun 2012-Apr 2017	Canada; adults aged >=18 years with cirrhosis admitted for liver transplantation and perioperative CT within 90 days (mean 54 +/- 11 years)	3 months after liver transplantation	Baseline at admission	Post-liver-transplant sarcopenia by L3 CT	Sarcopenia prevalence increased from 46% before to 62% after transplantation. Post-transplant sarcopenia was associated with longer hospital stay, more 1-year complications, and more infections. Post-transplant SMI was independently associated with pretransplant renal function.
Bhanji RA et al, 2019	Cohort study	293	2002-2006	USA; adults aged >=18 years undergoing liver transplantation (51.95 +/- 11.01 years)	Postoperative assessment (median 7.2 months)	Baseline at admission	Sarcopenia prevalence measured by CT muscle mass before and after liver transplantation	Pretransplant sarcopenia prevalence was 50.0%; after transplant, it increased to 60.9%, with 25.5% new onset and 74.5% persistent sarcopenia. Muscle mass decreased in many patients; male sex and non-NASH predicted post-transplant sarcopenia.
Welch C et al, 2019	Cohort study	7	Aug-Oct 2017	UK; patients aged >=65 years scheduled for colorectal surgery (mean 74.7 +/- 4.1 years)	One week after colorectal surgery or at discharge	Baseline at admission	Sarcopenia (EWGSOP)	Baseline sarcopenia prevalence was 28.6%, increasing to 75.0% at discharge. Median muscle thickness decreased from 4.17 to 3.47 cm (P=0.028), and gait speed from 0.89 to 0.83 m/s (P=0.043); grip strength did not change significantly.

Author, year	Study design	N	Intervention period	Participants	Intervention	Control	Main outcomes	Findings
Awad S et al, 2012	Cohort study	47	Not specified	UK; patients with esophagogastric cancer who underwent CT before and after neoadjuvant chemotherapy (63 +/- 12 years)	After chemotherapy	Baseline before treatment	Post-chemotherapy sarcopenia by L3 CT	Sarcopenia prevalence increased significantly from 27 patients (57.4%) before NAC to 37 (78.7%) after NAC (P=0.046). Estimated FFM decreased by 2.9 +/- 4.7 kg and FM by 1.3 +/- 3.2 kg. NAC was associated with increased sarcopenia prevalence.
Levy D et al, 2022	Cohort study	139	Mar-Jun 2020	France; survivors of COVID-19 requiring ICU and/or respiratory support; median age 62 years (range 29-82)	3 and 6 months after discharge	Baseline at admission	Post-discharge sarcopenia (EWGSOP2) incidence	At 3 months after discharge, 22 patients (16%) had sarcopenia and 5 (4%) severe sarcopenia; all had severe COVID-19. Sarcopenia was associated with longer hospital and ICU stay, more tracheostomy, and greater need for post-discharge rehab; by 6 months, two-thirds recovered but 6 (4%) had persistent sarcopenia.

Note: Rehabilitation is abbreviated as rehab where used.

Abbreviations: BMI, Body Mass Index; SMI, Skeletal Muscle Index; ADL, Activities of Daily Living; PA, Phase Angle; TNF-alpha, Tumor Necrosis Factor-alpha; OR, odds ratio; CT, computed tomography; ICU, intensive care unit; LT, liver transplantation; GFR, glomerular filtration rate; NASH, non-alcoholic steatohepatitis; hsCRP, high-sensitivity C-reactive protein; DHEA-S, dehydroepiandrosterone sulfate; AWGS, Asian Working Group for Sarcopenia; EWGSOP, European Working Group on Sarcopenia in Older People; OHS, open heart surgery; DM, diabetes mellitus; NAC, neoadjuvant chemotherapy; OGC, oesophagogastric cancer; FFM, fat-free mass; FM, fat mass; BATT, Bristol Activities of Daily Living Scale; IQR, interquartile range; SD, standard deviation.

Supplementary Table 20-1. Structured abstracts (FRQ3)

Author, year	Study design	N	Intervention period	Participants	Intervention	Control	Main outcomes	Findings
Bettger et al, 2020	RCT	287 (intervention 143 / control 144)	12 weeks (main evaluations at 6 and 12 weeks; medical costs at 12 months)	Adults after total knee arthroplasty	Home-based virtual physical therapy using VERA system	Usual care (in-person rehab)	Medical cost (12 weeks)	Medical costs were significantly lower in the intervention group; function and safety were generally equivalent.
DE Lima AP et al, 2022	RCT	49 (intervention 23 / control 26)	12 weeks	Adults aged >=18 years requiring cardiac rehab in a middle-income country	Exercise therapy, structured education, and remote adherence monitoring	Conventional cardiac rehab	Program adherence	Home-based, remotely monitored cardiac rehab had adherence and effectiveness equivalent to conventional rehab and lower service costs.
Kraal et al, 2014	RCT	55 (center 26 / home 29; analyzed 25 / 25)	12 weeks	Low- to moderate-risk cardiac rehab patients	Home-based cardiac rehab	Center-based cardiac rehab	Change in VO2max	Home-based CR may be an alternative to facility-based CR.
Lai B et al, 2020	RCT	20 (10 / 10)	8 weeks (24 sessions total)	Adults with Parkinson disease (Hoehn and Yahr 1-3)	One-to-one home exercise training with remote coaching	Self-managed home exercise	Number of completed sessions and attendance (adherence)	Remote coaching improved adherence compared with self-management.
Maddison R et al, 2019	RCT	162 (intervention 82 / control 80)	12 weeks	Adults diagnosed with coronary heart disease within 6 months	Remotely monitored exercise-based cardiac rehab (REMOTE-CR)	Center-based cardiac rehab	VO2max	REMOTE-CR was noninferior to facility-based programs and was cost-effective.
Matchar DB et al, 2022	RCT	266 (intervention 177 / control 89)	3 months after discharge (12 weeks)	Adults discharged after stroke for whom outpatient rehab continuation was recommended in Singapore	Education plus free transportation (T) or free transportation plus zero copayment (T&S)	Education only	Outpatient rehab participation (>=1 session), total sessions, time to dropout	Reducing transportation and cost barriers may improve persistence in outpatient rehab participation.
Meziere et al, 2021	RCT	27 (intervention 14 / control 13)	3 months (twice weekly within home-helper visits)	Community-dwelling older adults receiving home-care services	Exercise training using T4H app plus IT support/equipment	Usual home-care support without exercise intervention	Composite of no injurious falls plus improved TUG or BI	Feasibility and engagement were high, but between-group difference in fall-prevention effect was not significant.

Author, year	Study design	N	Intervention period	Participants	Intervention	Control	Main outcomes	Findings
Takeda et al, 2022	Pilot RCT	54 (intervention 28 / control 26 at final evaluation)	12 weeks	Community-dwelling older adults requiring care and using day care	Home exercise instruction and buddy-style mutual support	Usual care	Rehab continuation and exercise adherence	Adherence improved significantly, while between-group differences in physical function were generally not observed.

Note: Rehabilitation is abbreviated as rehab where used.

Abbreviations: TKA, total knee arthroplasty; CR, cardiac rehabilitation; CHD, coronary heart disease; VO2max, maximal oxygen consumption; TUG, Timed Up and Go; BI, Barthel Index; VERA, Virtual Exercise Rehabilitation Assistant; T/T&S, Transportation/Transportation and Sessions; T4H, Tech for Health (exercise app).
